# Supplementary material for: Inhibition of lung microbiota-derived proapoptotic peptides ameliorates acute exacerbation of pulmonary fibrosis
Source: Nat Commun. 2022 Mar 23;13:1558. doi: 10.1038/s41467-022-29064-3 (PMC8943153; doi:10.1038/s41467-022-29064-3)

Supplementary Information

Inhibition of Lung Microbiota-derived Proapoptotic Peptides Ameliorate Acute Exacerbation of Pulmonary Fibrosis

Corina N. D’Alessandro-Gabazza et al.

```
S. nepalensis CNDG_00351          MKKTILASSLAVALGVTGYAATSDNNQAHASEQNIDKAHLAELALNGSAELDQQPLHAGA
S. haemolyticus_1bp_00353         MKKKVIASSLAVALGVTGYALTTDNS-AHASESTTNYAQLANLAQNNPSELNAHPVQAGA
S. haemolyticus_12bp_00350        MKKKVIASSLAVALGVTGYALTTDNS-AHASESTTNYAQLANLAQNNPSELNAHPVQAGA
S. haemolyticus_7bp_00350         MKKKVIASSLAVALGVTGYALTTDNS-AHASESTTNYAQLANLAQNNPSELNAHPVQAGA

S. nepalensis CNDG_00351          YNYNFVLDGNEFIFTSDGNTWSWGYHAAGTQASSSNTTQDVSSEVSVNTNEKSASEVRSQ
S. haemolyticus_1bp_00353         YNITFVKDGFKYNFTSDGQSWSWNYTYVGG-ADTVATTQAAPAAQSTDYSASYSNEASTQ
S. haemolyticus_12bp_00350        YNITFVKDGFKYNFTSDGQSWSWNYTYVGG-ADTVATTQAAPAAQSTDYSASYSNEASTQ
S. haemolyticus_7bp_00350         YNITFVKDGFKYNFTSDGQSWSWNYTYVGG-ADTVATTQAAPAAQSTDYSASYSNEASTQ

S. nepalensis CNDG_00351          QSYATPVTVAAPKASASTNVRTTQTSVAPK--AYNVAQTSAASTGGSVKAQFLAAGGSEA
S. haemolyticus_1bp_00353         -------SVSSNQQASNTNVEAVS---APKTTSYSASTSSSASTGGSVKAQFLANGGTEA
S. haemolyticus_12bp_00350        -------SVSSNQQASNTNVEAVS---APKTTSYSASTSSSASTGGSVKAQFLANGGTEA
S. haemolyticus_7bp_00350         -------SVSSNQQASNTNVEAVS---APKTTSYSASTSSSASTGGSVKAQFLANGGTEA

S. nepalensis CNDG_00351          MWNSIVMPESSGNPNAVNPAGYRGLGQTKESWGTGSVADQTKGMLNYAKQRYGSEEAALA
S. haemolyticus_1bp_00353         AWNAIVMPESGGNPNAVNPAGYRGLGQTMESWGTGSVASQTKGMINYANSRYGSLDAAIA
S. haemolyticus_12bp_00350        AWNAIVMPESGGNPNAVNPAGYRGLGQTMESWGTGSVASQTKGMINYANSRYGSLDAAIA
S. haemolyticus_7bp_00350         AWNAIVMPESGGNPNAVNPAGYRGLGQTMESWGTGSVASQTKGMINYANSRYGSLDAAIA

S. nepalensis CNDG_00351          FRASHGWW
S. haemolyticus_1bp_00353         FRANNGWW
S. haemolyticus_12bp_00350        FRANNGWW
S. haemolyticus_7bp_00350         FRANNGWW
```

Supplementary Fig. 1. An alignment of the putative transglycosylase containing the corisin peptide in *Staphylococcus nepalensis* strain CNDG (CNDG) with the homologs in three strains of *Staphylococcus haemolyticus* isolated from the same lung fibrotic tissue in a mouse. The corisin peptide is bolded and shaded in blue, where amino acids are conserved. Outside of the corisin peptide sequence, the conserved amino acids are shaded in black, with similar amino acids shaded in grey. *Staphylococcus haemolyticus* 1bp_00353,12bp_00350, 7bp_00350 are *S. haemolyticus* strain 1 protein number 353, *S. haemolyticus* strain 12 protein number 350, and *S. haemolyticus* strain 7 protein number 350, respectively. The protein of *S. nepalensis* strain CNDG is encoded at open reading frame (ORF) position 351 in the genome, counting from the DnaA encoding gene (ORF00001). Amino acids with similar properties are grouped as LIMV, AG, YWF, DEQN, KRH, and ST. The alignment was carried out with Multiple Sequence Comparison by Log-Expectation (MUSCLE at https://www.ebi.ac.uk/Tools/msa/muscle/).

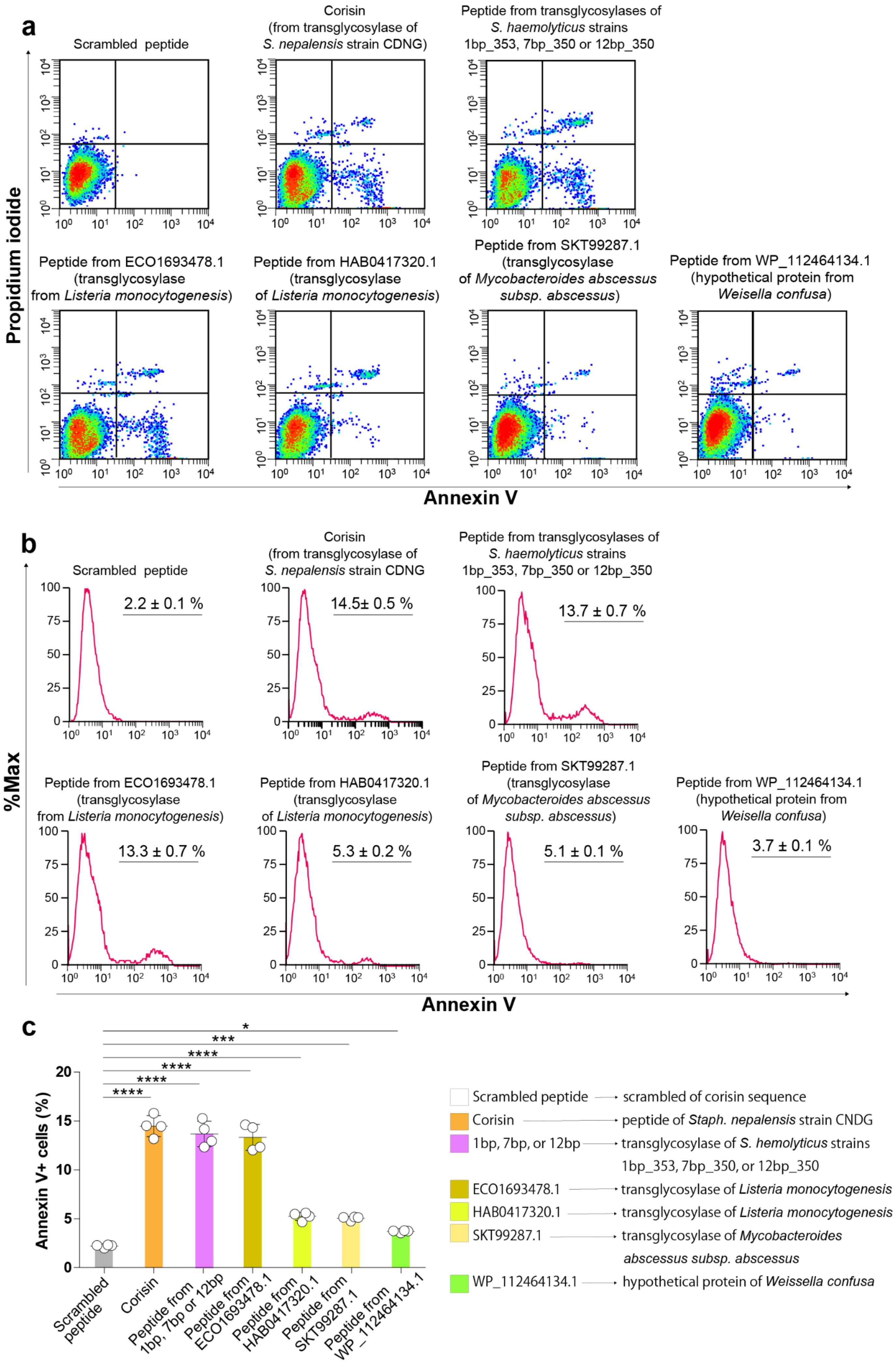
a
Scrambled peptide
Corisin (from transglycosylase of S. nepalensis strain CDNG)
Peptide from transglycosylases of S. haemolyticus strains 1bp_353, 7bp_350 or 12bp_350
Peptide from ECO1693478.1 (transglycosylase from Listeria monocytogenesis)
Peptide from HAB0417320.1 (transglycosylase of Listeria monocytogenesis)
Peptide from SKT99287.1 (transglycosylase of Mycobacteroides abscessus subsp. abscessus)
Peptide from WP_112464134.1 (hypothetical protein from Weisella confusa)
Propidium iodide
Annexin V
b
2.2 ± 0.1 %
14.5± 0.5 %
13.7 ± 0.7 %
13.3 ± 0.7 %
5.3 ± 0.2 %
5.1 ± 0.1 %
3.7 ± 0.1 %
%Max
Annexin V
c
Annexin V+ cells (%)
Scrambled peptide
Corisin
Peptide from 1bp, 7bp or 12bp
Peptide from ECO1693478.1
Peptide from HAB0417320.1
Peptide from SKT99287.1
Peptide from WP_112464134.1
Scrambled peptide → scrambled of corisin sequence
Corisin → peptide of Staph. nepalensis strain CNDG
1bp, 7bp, or 12bp → transglycosylase of S. hemolyticus strains 1bp_353, 7bp_350, or 12bp_350
ECO1693478.1 → transglycosylase of Listeria monocytogenesis
HAB0417320.1 → transglycosylase of Listeria monocytogenesis
SKT99287.1 → transglycosylase of Mycobacteroides abscessus subsp. abscessus
WP_112464134.1 → hypothetical protein of Weissella confusa

Supplementary Fig. 2. The synthetic corisin-like peptides of transglycosylases from several pathogens induce apoptosis of alveolar epithelial cells. a, **b** A549 alveolar epithelial cells were cultured in the presence of 10 μg/ml of corisin-like peptides prepared by a company for 48h to evaluate apoptosis. A549 cells treated with synthetic corisin peptide of *S. nepalensis* or its scrambled equivalent were the positive and negative controls, respectively. **b**, **c** The percentage of apoptotic cells was determined by flow cytometry and quantified. N=4 in each group. Data are expressed as the mean ± S.D. Statistical analysis was performed by ANOVA with a post hoc Newman-Keuls test. *$p<0.05$; ***$p<0.001$; ****$p<0.0001$. S., *Staphylococcus*. The source data underlying **c** are provided in the Source Data file.

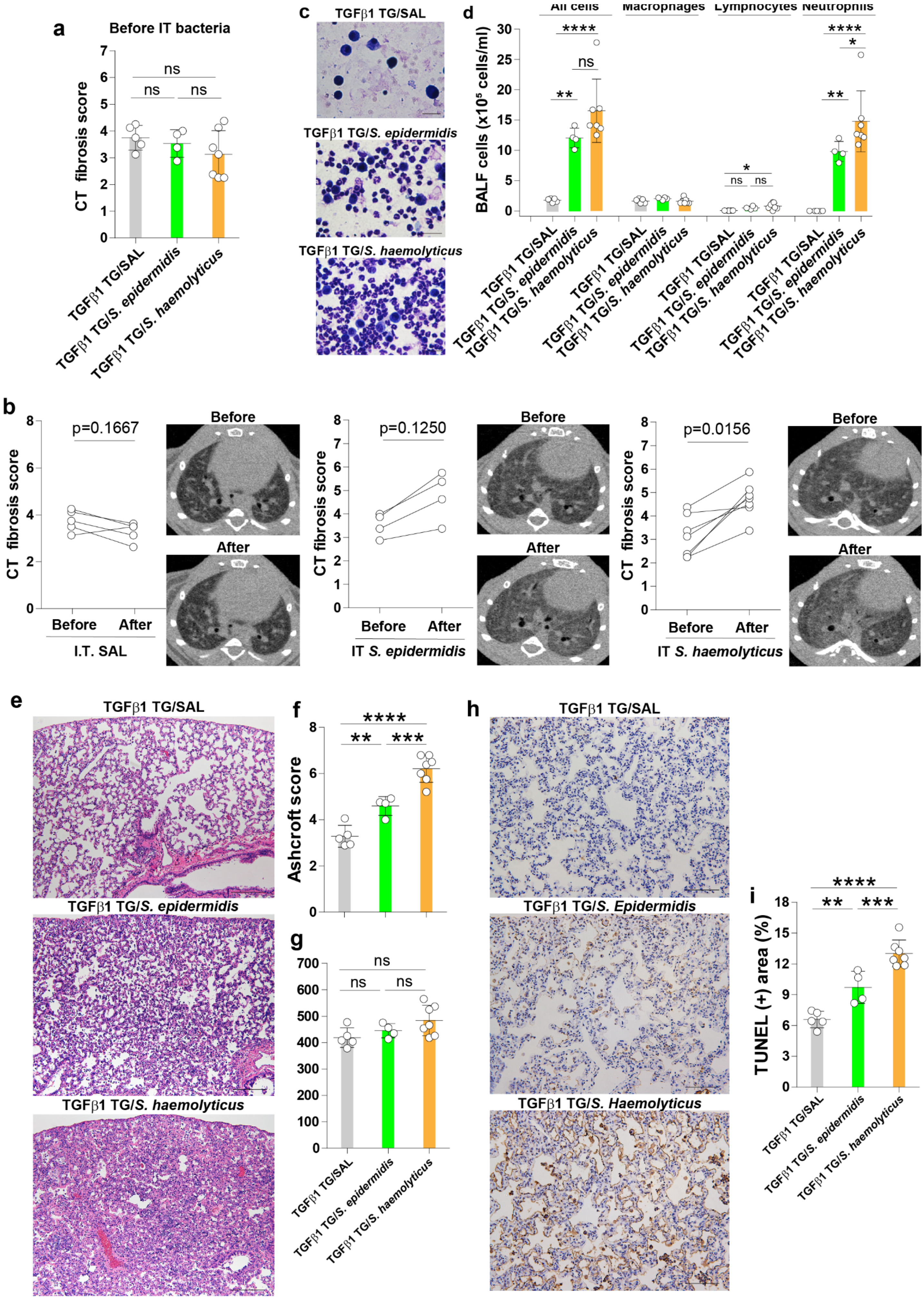

a
Before IT bacteria
CT fibrosis score
ns
TGFβ1 TG/SAL
TGFβ1 TG/S. epidermidis
TGFβ1 TG/S. haemolyticus
c
TGFβ1 TG/SAL
TGFβ1 TG/S. epidermidis
TGFβ1 TG/S. haemolyticus
d
All cells
Macrophages
Lymphocytes
Neutrophils
BALF cells (x10⁵ cells/ml)
b
p=0.1667
p=0.1250
p=0.0156
Before
After
I.T. SAL
IT S. epidermidis
IT S. haemolyticus
e
TGFβ1 TG/SAL
TGFβ1 TG/S. epidermidis
TGFβ1 TG/S. haemolyticus
f
Ashcroft score
g
h
TGFβ1 TG/SAL
TGFβ1 TG/S. Epidermidis
TGFβ1 TG/S. Haemolyticus
i
TUNEL (+) area (%)

Supplementary Fig. 3. Intra-pulmonary Instillation of the corisin-containing *Staphylococcus haemolyticus* strain 12 induces acute exacerbation of pulmonary fibrosis in germ-free hTGFβ1 TG mice. a Germ-free transforming growth factor (TGF)β1 transgenic (TG) mice with matched lung fibrosis based on computed tomography (CT) score received intra-tracheal instillation of saline (n=5), *Staphylococcus epidermidis* ATCC14990 (n=4) or *Staphylococcus haemolyticus* strain 12 (n=7) as described under methods. Data are expressed as the mean ± S.D. Statistical analysis was performed by ANOVA with a post hoc Newman-Keuls test. ns, not significant. **b** CT was performed before and after intra-tracheal instillation of SAL, *S. epidermidis*, or *S. haemolyticus*. Exacerbation of lung fibrosis was scored and compared. Statistical analysis by two-tailed paired Wilcoxon signed rank test. Representative CT findings before and after treatment in each group are shown. **c**, **d** The number of cells in bronchoalveolar lavage fluid (BALF) was counted and then stained with Giemsa on the second day after intra-tracheal instillation of saline or each bacterium. Scale bars indicate 20 µm. N=5 in TGFβ1 TG/SAL, n=4 in TGFβ1 TG/*S. epidemidis* and n=7 in TGFβ1 TG/*S. haemolyticus* groups. Bars indicate the means ± S.D. Representative microphotograph of stained BALF cells in each group is shown. Statistical analysis by ANOVA with a post hoc Newman-Keuls test. *p<0.05; **p<0.01; ****p<0.0001. **e**, **f** Lung specimens from each group was stained with hematoxylin and eosin, and the pathological findings were scored using the Ashcroft fibrosis score as described under Methods. Representative lung microphotograph of each group is shown. N=5 in TGFβ1 TG/SAL, n=4 in TGFβ1 TG/*S. epidemidis* and n=7 in TGFβ1 TG/*S. haemolyticus* groups. Scale bars indicate 200 µm. Bars indicate the means ± S.D. Statistical analysis by ANOVA with a post hoc Newman-Keuls test. **p<0.01; ***p<0.001; ****p<0.0001. **g** Lung hydroxyproline content was measured using a colorimetric assay as described under Methods. N=5 in TGFβ1 TG/SAL, n=4 in TGFβ1 TG/*S. epidemidis* and n=7 in TGFβ1 TG/*S. haemolyticus* groups. Bars indicate the means ± S.D. Statistical analysis by ANOVA with a post hoc Newman-Keuls test. ns, not significant. **h, i** DNA fragmentation was evaluated by staining with terminal deoxynucleotidyltransferase dUTP Nick-End Labeling (TUNEL) and then quantified using the image WinRoof software. Representative lung microphotograph of each group is shown. Scale bars indicate 100 µm. N=5 in TGFβ1 TG/SAL, n=4 in TGFβ1 TG/*S. epidemidis* and n=7 in TGFβ1 TG/*S. haemolyticus* groups. Bars indicate the means ± S.D. Statistical analysis by ANOVA with a post hoc Newman-Keuls test. **p<0.01; ****p<0.0001. The source data underlying **a**, **b**, **d, f, g, and i** are provided in the Source Data file.

```
W. confusa (WP_112464134.1)      MKKMSKTAILTALALASFGATTVIIQSDFMPNAQVSANVANGYSNGLYYTNGSLANGYIN     60
S. nepal. CNDG-00351             ---MKKTILASSLAVA-LGVTGYAATSDNNQAHASEQNIDKA----------HLA-----     41
L. monocyt. (HAB0417320.1)       ---MKKSILASSLAVA-LGVTGYSLATDGNEAQASEQNVDYA----------HLA-----     41
Mycob. absc. (SKR69498.1)        ---MKKTFIASTLALT-LGATGYAVSGHE-AHASETTNVDQA----------HLV-----     40
Mycob. absc. (SKT99287.1)        ---MKKTVIASSLAVT-LGLTGYALTNDH-SAHASEQTTNYS----------HLA-----     40
S. hemol_00350                   ---MKKKVIASSLAVA-LGVTGYALTTDN-SAHASESTTNYA----------QLA-----     40
L. monocyt. (ECO1693478.1)       ---MKKTVIASSLAVA-LGVTGYALTTDN-SAHASESTTNYA----------QLA-----     40

W. confusa (WP_112464134.1)      DGQNWYLFKNGQKLSEIQQYMGGYYYFDKTTHLRTDNAFRDEWGNTYYFGGDGRAVTGLQ     120
S. nepal. CNDG-00351             -----ELALNGSAELDQQPLHAGAYNY-----------------------------NF     65
L. monocyt. (HAB0417320.1)       -----DLAQNHPSELNAAPIQEGAYDI-----------------------------HF     65
Mycob. absc. (SKR69498.1)        -----DLAHNHPEQLNAAPVQEGAYDI-----------------------------HF     64
Mycob. absc. (SKT99287.1)        -----DLAQNNPSELNAHPVQAGAYDI-----------------------------SF     64
S. hemol_00350                   -----NLAQNNPSELNAHPVQAGAYNI-----------------------------TF     64
L. monocyt. (ECO1693478.1)       -----SLAQNNPSELNAHPVQAGAYNI-----------------------------TF     64

W. confusa (WP_112464134.1)      TINGNKYYFGDDGTYTLRKSQWLTIGGAKYYATADGSFASDVTKIGNTYYYFDPSTKALS     80
S. nepal. CNDG-00351             VLDGNEFIFTSDGNTWS--WGYHAAGTQASSSNTTQD-----VSSEVSVNTNEKSASEVR     118
L. monocyt. (HAB0417320.1)       VYNGNAYNFTSDGHSWE--WQWYYVGSASNDVADVST-----AASTVSYE---NTAADVQ     115
Mycob. absc. (SKR69498.1)        VSGGFEYNFTSDGTNFS--WNYQEAGSTSAQT---SNTAVQSADYTTSYN-QEAGTQSVS     118
Mycob. absc. (SKT99287.1)        VKDGFKYNFTSNGNTWS--WNYTYTGGADTAQST--------TDYTESY--NQASTQSVS     112
S. hemol_00350                   VKDGFKYNFTSDGQSWS--WNYTYVGGADTVATTQAAPAAQSTDYSASYS-NEASTQSVS     121
L. monocyt. (ECO1693478.1)       VKDGFKYNFTSDGQSWS--WDYIYVGGADTVATTQAAPAAQSTDYSASYS-NEASTQSVS     121

W. confusa (WP_112464134.1)      TKRNYIQARWGSWYLVGNDGTVQSGLQSWAGNYYYFDPSTYLKVTNTTININGVDWYFDG     240
Staph. nepal. CNDG-00351         SQQ----S----YA---TPVTVAAPKASAST--------NVR-TTQTS---VA----PKA     151
L. monocyt. (HAB0417320.1)       GQQ---QS---SNY---NVEAVSAPTQSESTSSY-TSSRNYS-TTQT--AAPA----TRS     158
Mycob. absc. (SKR69498.1)        SNQ---QS---SNT---NVEAVSAPTTSNNGSN-----HNYS-TKTTSYSAPS----T--     157

Mycob. absc. (SKT99287.1)        SNN---QA---STS---NVKAVSAPVQRTS-SY-----NNYS-ARTTSYSAPK----T--     150
S. hemol_00350                   SNQ---QA---SNT---NVEAVSAPKTT-------------------SYSA---------     144
L. monocyt. (ECO1693478.1)       SNQ---QA---SNT---NVEAVSAPKTT-------------------SYSA---------     144

W. confusa (WP_112464134.1)      SGVGTKKASSANQGSVYDQFIAAGGTDAMWQYIVMPESGGNPNAVSPNGYRGLGQTKQSW     300
S. nepal. CNDG-00351             YNVAQ-TSAASTGGSVKAQFLAAGGSEAMWNSIVMPESSGNPNAVNPAGYRGLGQTKESW     210
L. monocyt. (HAB0417320.1)       YSVAQTSAPASTGGSVKSQFLAAGGNEAMWNAIVLPESGGNPNAVNPAGYRGLGQTMESW     218
Mycob. absc. (SKR69498.1)        -------SSASTGGSTKAQFLANGGTEEAWNAIVMPESGGNPNAVNPAGYRGLGQTMESW     210
Mycob. abcs. (SKT99287.1)        ----TSYSTASTGGSVKAQFLANGGTEAAWNAIVMPESSGNPNASN-GQYHGLGQTNQSW     205
S. hemol_00350                   ----STSSSASTGGSVKAQFLANGGTEAAWNAIVMPESGGNPNAVNPAGYRGLGQTMESW     200
L. monocyt. (ECO1693478.1)       ----STSSSASTGGSVKAQFLANGGTEAAWNAIVMPESGGNPNAVNPAGYRGLGQTMESW     200

W. confusa (WP_112464134.1)      GTGTVAQQTAGMINYAVTRYGSIDNAIKFRQANGWW     336
S. nepal. CNDG-00351             GTGSVADQTKGMLNYAKQRYGSEEAALAFRASHGWW     246
L. monocyt. (HAB0417320.1)       GTGSVANQTKGMLNYAQQRYGSVDAAIAFRANHGWW     254
Mycob. absc. (SKR69498.1)        GTGSVASQTKGMLNYANSRYGSLSNAIAFRQSHGWW     246
Mycob. Abcs. (SKT99287.1)        GTGSVASQTQGMANYAKSRYGSWDAAIAFRNANGWW     241
S. hemol_00350                   GTGSVASQTKGMINYANSRYGSLDAAIAFRANNGWW     236
L. monocyt. (ECO1693478.1)       GTGSVASQTKGMINYANSRYGSLDAAIAFRANNGWW     236
```

Supplementary Fig. 4. An alignment of corisin-containing putative transglycosylases from diverse bacterial pathogens. The names of the organisms have been abbreviated as: *W. confusa* (*Weissella confusa*, Genbank protein accession no. WP_112464134.1), *S. nepal*. CNDG-00351 (*S. nepalensis* strain CNDG protein 00351), *L. monocyt.* (*Listeria monocytogenes*, Genbank protein accession no. HAB0417320.1), *Mycob. absc.* (*Mycobacteroides abscessus*, Genbank protein accession no. SKR69498.1), *Mycob. absc.* (*Mycobacteroides abscessus*, Genbank protein accession no. SKT99287.1), *S. hemol*_00350 (*Staphylococcus haemolyticus* 12b protein 00350), and *L. monocyt.* (*L. monocytogenes*, Genbank protein accession no. ECO1693478.1). The corisin peptide is bolded and shaded in blue, where amino acids are conserved. Outside of the corisin peptide sequence, the conserved amino acids are shaded in black, with similar amino acids shaded in grey. Amino acids with similar properties are grouped as LIMV, AG, YWF, DEQN, KRH, and ST. S., *Staphylococcus*. Alignment was carried out with Multiple Sequence Comparison by Log-Expectation (MUSCLE at https://www.ebi.ac.uk/Tools/msa/muscle/).

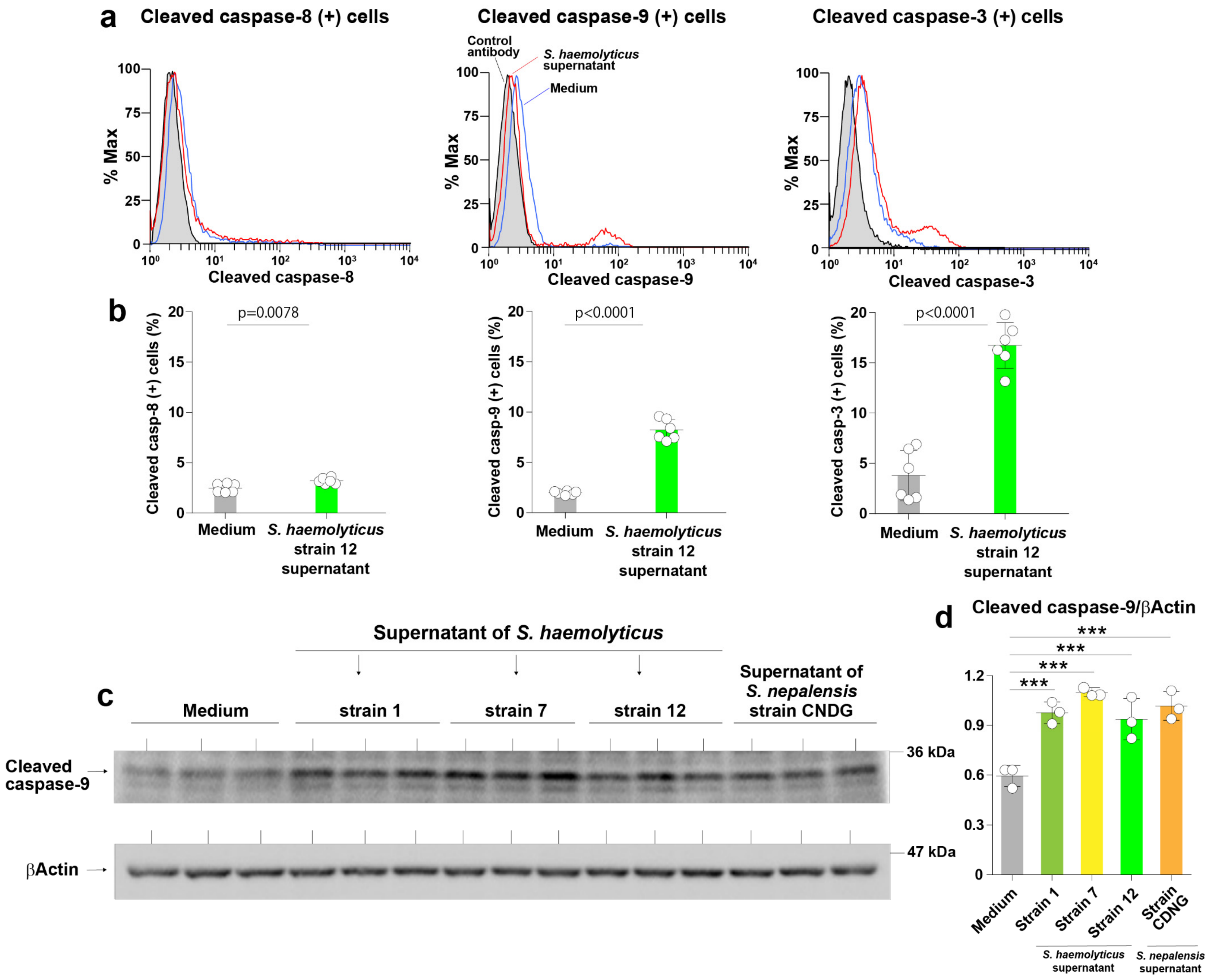
a
Cleaved caspase-8 (+) cells
Cleaved caspase-9 (+) cells
Cleaved caspase-3 (+) cells
Control antibody
S. haemolyticus supernatant
Medium
% Max
Cleaved caspase-8
Cleaved caspase-9
Cleaved caspase-3
b
p=0.0078
p<0.0001
p<0.0001
Cleaved casp-8 (+) cells (%)
Cleaved casp-9 (+) cells (%)
Cleaved casp-3 (+) cells (%)
Medium
S. haemolyticus strain 12 supernatant
c
Supernatant of S. haemolyticus
Medium
strain 1
strain 7
strain 12
Supernatant of S. nepalensis strain CNDG
Cleaved caspase-9
βActin
36 kDa
47 kDa
d
Cleaved caspase-9/βActin
***
Medium
Strain 1
Strain 7
Strain 12
Strain CDNG
S. haemolyticus supernatant
S. nepalensis supernatant

Supplementary Fig. 5. Apoptosis pathway induced by *S. haemolyticus* culture supernatant. a, b A549 alveolar epithelial cells were cultured in the presence of the (1/10 dilution) culture supernatants from *S. haemolyticus* strain 12 for 24h. The percentage of cells positive for cleaved caspase-8, cleaved caspase-9, and cleaved caspase-3 was determined by flow cytometry and quantified. N=6 in each group. Data are expressed as the mean ± S.D. Statistical analysis was performed using a two-sided unpaired t-test. **c**, **d** A549 alveolar epithelial cells were cultured in the presence of the (1/10 dilution) culture supernatants from *S. haemolyticus* strains 1, 7, 12, and from *S. nepalensis* for 24h. Cleaved caspase-9 was evaluated by Western blotting and quantified using image software. Representative blots of two experiments with similar results are shown. N=3 in each group. Data are expressed as the mean ± S.D. Statistical analysis was performed by ANOVA with a post hoc Newman-Keuls test. ***p<0.001. The source data underlying **b** and **d** are provided in the Source Data file.

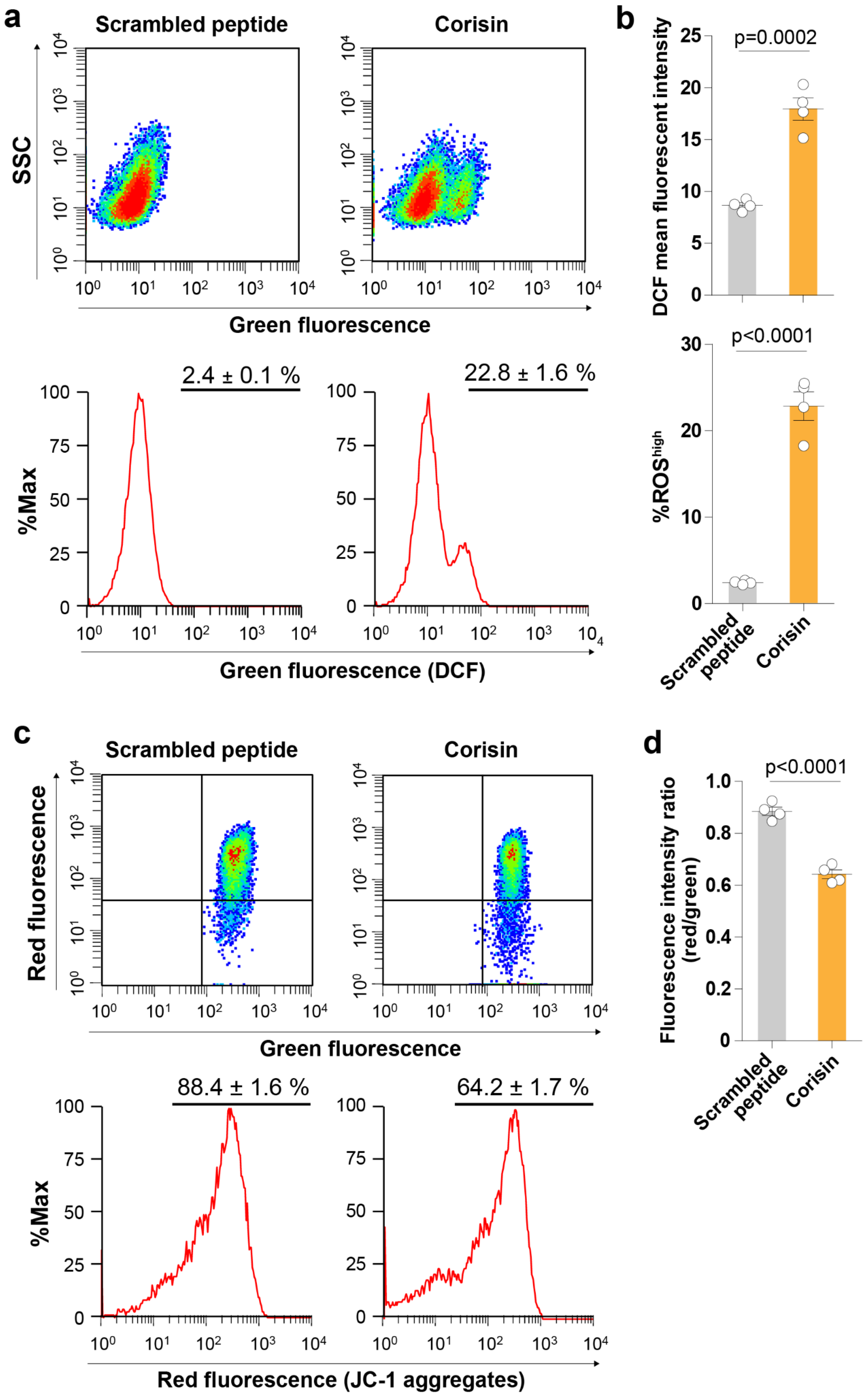
a
Scrambled peptide
Corisin
SSC
Green fluorescence
2.4 ± 0.1 %
22.8 ± 1.6 %
%Max
Green fluorescence (DCF)
b
DCF mean fluorescent intensity
p=0.0002
%ROS^high
p<0.0001
Scrambled peptide
Corisin
c
Scrambled peptide
Corisin
Red fluorescence
Green fluorescence
88.4 ± 1.6 %
64.2 ± 1.7 %
%Max
Red fluorescence (JC-1 aggregates)
d
Fluorescence intensity ratio (red/green)
p<0.0001
Scrambled peptide
Corisin

Supplementary Fig. 6. Increased generation of reactive oxygen species (ROS) and loss of mitochondrial membrane integrity after treatment with corisin. a, **b** A549 alveolar epithelial cells were treated with 5 µM corisin or scrambled peptide, and after washing, incubated with medium containing 2',7'-dichlorofluorescin diacetate before evaluating by flow cytometry. N=4 in Scrambled peptide and Corisin groups examined in two independent experiments. Data are expressed as the mean ± S.D. Statistical analysis by two-sided unpaired t-test. **c**, **d** Mitochondrial membrane integrity was evaluated using the JC-1 dye as described under Methods. N=4 in Scrambled peptide and Corisin groups examined in two independent experiments. Data are expressed as the mean ± S.D. Statistical analysis by two-sided unpaired t-test. SSC, side scatter. The source data underlying **b** and **d** are provided in the Source Data file.

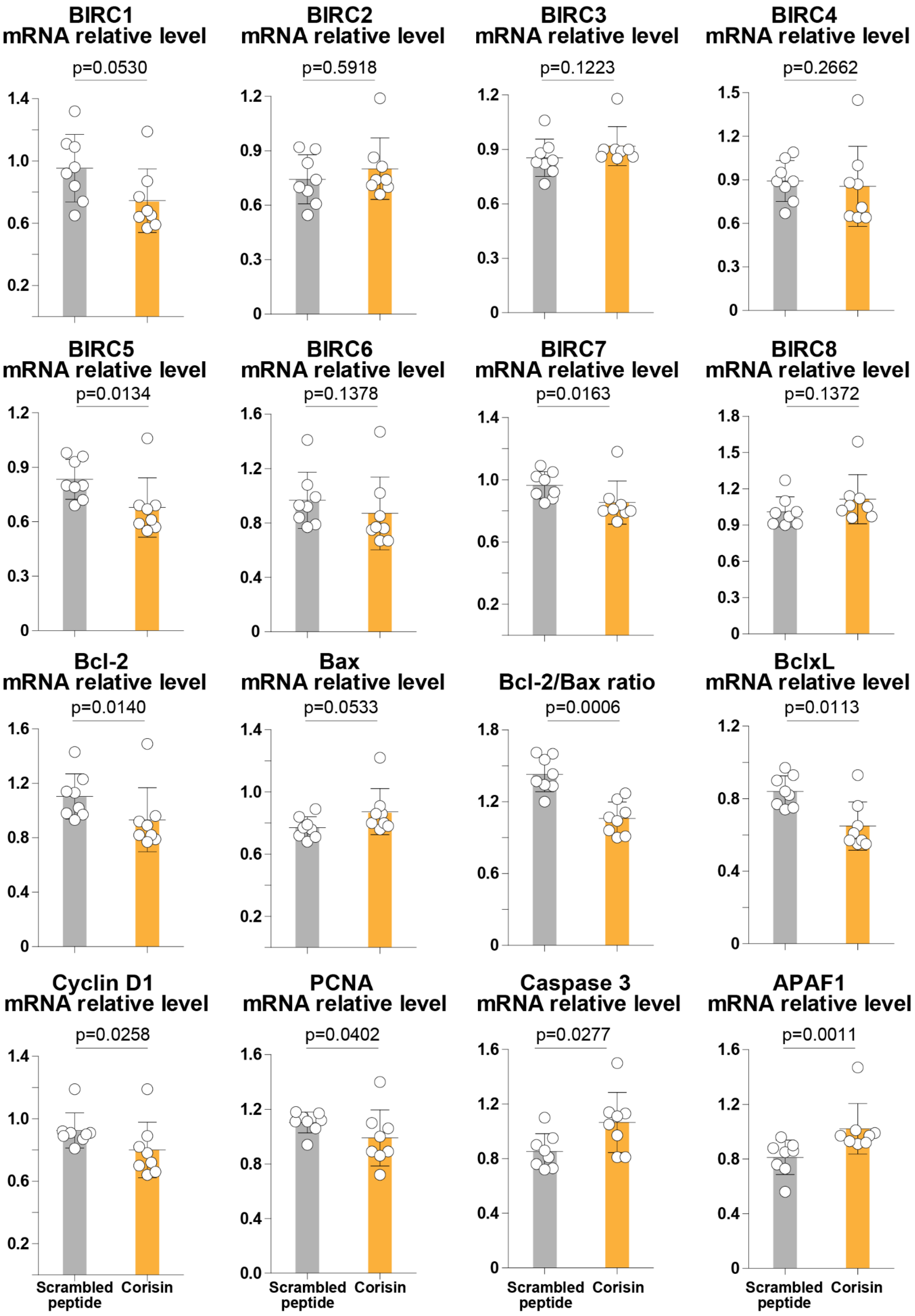

BIRC1
mRNA relative level
p=0.0530
1.4
1.0
0.6
0.2
BIRC2
mRNA relative level
p=0.5918
1.2
0.9
0.6
0.3
0
BIRC3
mRNA relative level
p=0.1223
1.2
0.9
0.6
0.3
0
BIRC4
mRNA relative level
p=0.2662
1.5
1.2
0.9
0.6
0.3
0
BIRC5
mRNA relative level
p=0.0134
1.2
0.9
0.6
0.3
0
BIRC6
mRNA relative level
p=0.1378
1.6
1.2
0.8
0.4
0
BIRC7
mRNA relative level
p=0.0163
1.4
1.0
0.6
0.2
BIRC8
mRNA relative level
p=0.1372
1.8
1.5
1.2
0.9
0.6
0.3
0
Bcl-2
mRNA relative level
p=0.0140
1.6
1.2
0.8
0.4
0
Bax
mRNA relative level
p=0.0533
1.4
1.0
0.6
0.2
Bcl-2/Bax ratio
p=0.0006
1.8
1.2
0.6
0
BclxL
mRNA relative level
p=0.0113
1.2
0.8
0.4
0
Cyclin D1
mRNA relative level
p=0.0258
1.4
1.0
0.6
0.2
PCNA
mRNA relative level
p=0.0402
1.6
1.2
0.8
0.4
0.0
Caspase 3
mRNA relative level
p=0.0277
1.6
1.2
0.8
0.4
0
APAF1
mRNA relative level
p=0.0011
1.6
1.2
0.8
0.4
0
Scrambled peptide
Corisin
Scrambled peptide
Corisin
Scrambled peptide
Corisin
Scrambled peptide
Corisin

Supplementary Fig. 7. Corisin alters the mRNA expression of proapoptotic and antiapoptotic factors. A549 alveolar epithelial cells were cultured in the presence of 50 µM corisin for 24h, total mRNA was extracted, and cDNA was prepared before amplification by RT-PCR. N=8 in each group. Data are expressed as the mean ± S.D. Statistical analysis was performed using the two-sided Mann-Whitney test. The source data underlying each panel are provided in the Source Data file.

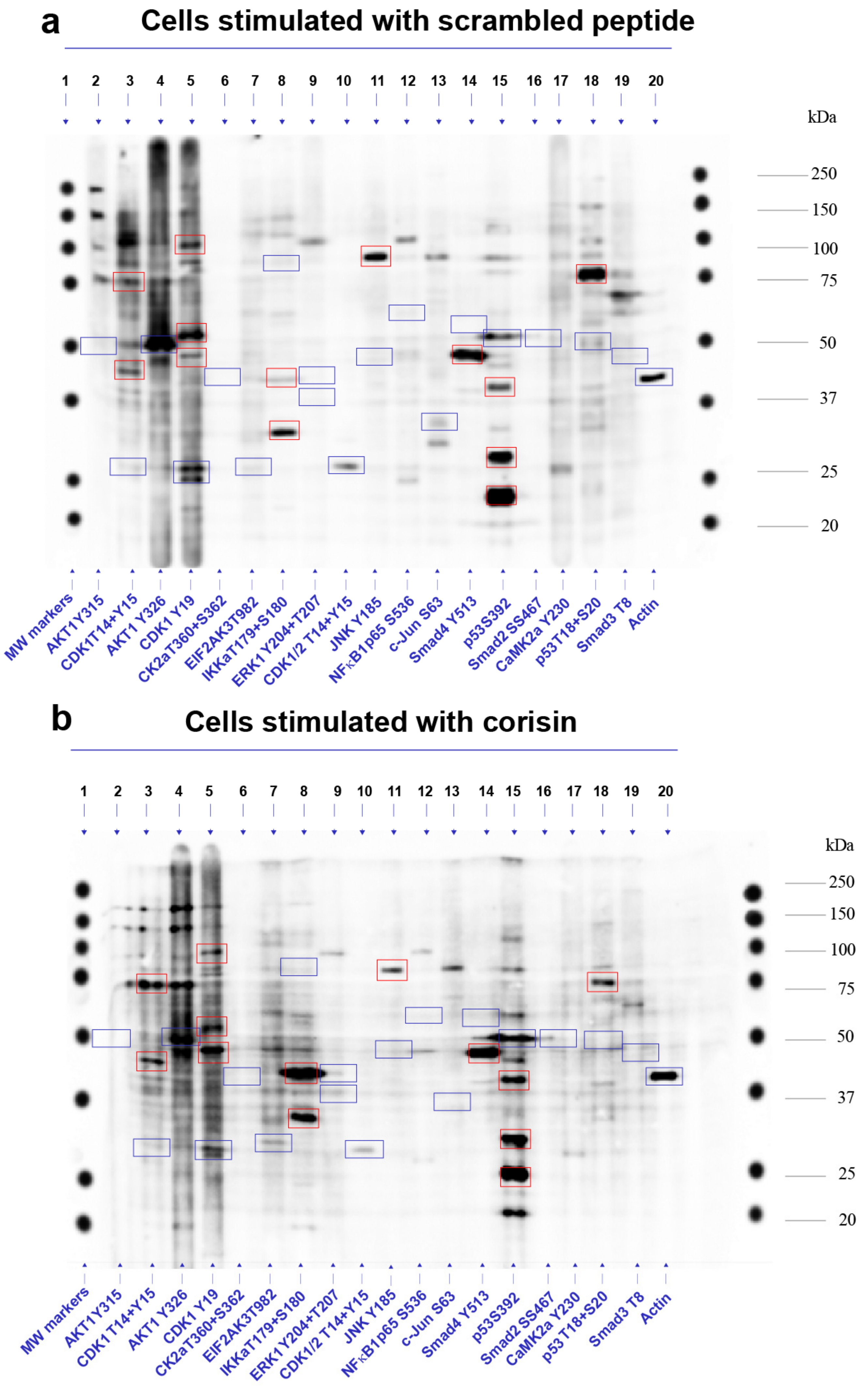

a
Cells stimulated with scrambled peptide
1 2 3 4 5 6 7 8 9 10 11 12 13 14 15 16 17 18 19 20
kDa
250
150
100
75
50
37
25
20
MW markers
AKT1Y315
CDK1T14+Y15
AKT1 Y326
CDK1 Y19
CK2aT360+S362
EIF2AK3T982
IKKaT179+S180
ERK1 Y204+T207
CDK1/2 T14+Y15
JNK Y185
NFκB1p65 S536
c-Jun S63
Smad4 Y513
p53S392
Smad2 SS467
CaMK2a Y230
p53T18+S20
Smad3 T8
Actin
b
Cells stimulated with corisin
1 2 3 4 5 6 7 8 9 10 11 12 13 14 15 16 17 18 19 20
kDa
250
150
100
75
50
37
25
20
MW markers
AKT1Y315
CDK1 T14+Y15
AKT1 Y326
CDK1 Y19
CK2aT360+S362
EIF2AK3T982
IKKaT179+S180
ERK1 Y204+T207
CDK1/2 T14+Y15
JNK Y185
NFκB1p65 S536
c-Jun S63
Smad4 Y513
p53S392
Smad2 SS467
CaMK2a Y230
p53T18+S20
Smad3 T8
Actin

Supplementary Fig. 8. Screening of phosphoproteins by immunoblotting. After overnight starvation, A549 alveolar epithelial cells were cultured in the presence of 100 μM corisin (**a**) or scrambled peptide (**b**) for 30 min before preparing cell lysate as described under Methods. Representative blots from two independent experiments with similar results are shown. Phosphorylated proteins were screened by immunoblotting at Kinexus^{TM} Corporation. Blue squares indicate target protein. The red square shows cross-reactive proteins. MW, molecular weight.

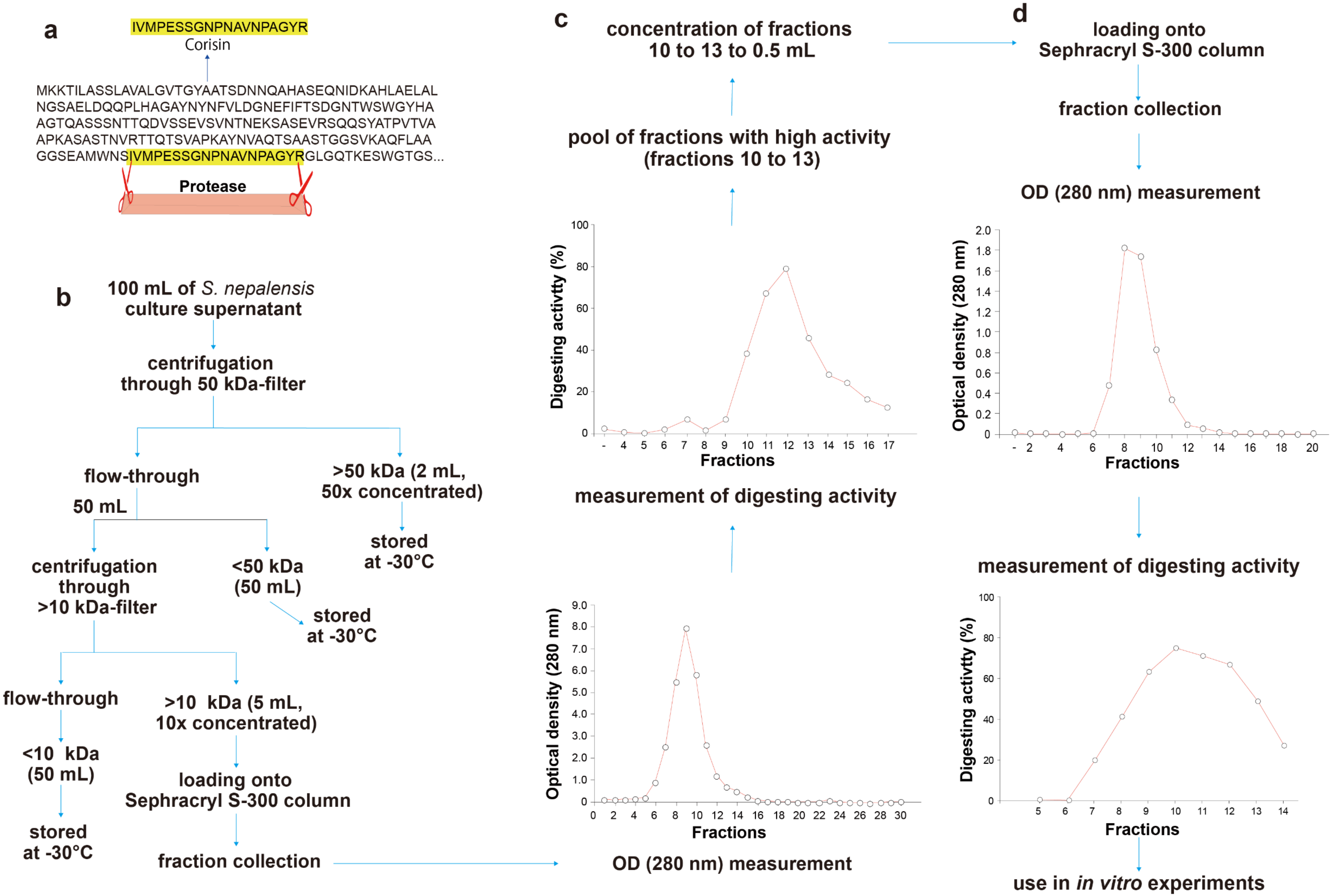

a
IVMPESSGNPNAVNPAGYR
Corisin
MKKTILASSLAVALGVTGYAATSDNNQAHASEQNIDKAHLAELAL
NGSAELDQQPLHAGAYNYNFVLDGNEFIFTSDGNTWSWGYHA
AGTQASSSNTTQDVSSEVSVNTNEKSASEVRSQQSYATPVTVA
APKASASTNVRTTQTSVAPKAYNVAQTSAASTGGSVKAQFLAA
GGSEAMWNSIVMPESSGNPNAVNPAGYRGLGQTKESWGTGS...
Protease
b
100 mL of S. nepalensis culture supernatant
centrifugation through 50 kDa-filter
flow-through
50 mL
>50 kDa (2 mL, 50x concentrated)
stored at -30°C
centrifugation through >10 kDa-filter
<50 kDa (50 mL)
stored at -30°C
flow-through
>10 kDa (5 mL, 10x concentrated)
<10 kDa (50 mL)
stored at -30°C
loading onto Sephracryl S-300 column
fraction collection
c
OD (280 nm) measurement
Optical density (280 nm)
Fractions
measurement of digesting activity
Digesting activty (%)
Fractions
pool of fractions with high activity (fractions 10 to 13)
concentration of fractions 10 to 13 to 0.5 mL
d
loading onto Sephracryl S-300 column
fraction collection
OD (280 nm) measurement
Optical density (280 nm)
Fractions
measurement of digesting activity
Digesting activty (%)
Fractions
use in in vitro experiments

Supplementary Fig. 9. Degradation of corisin-containing transglycosylase by the culture supernatant from *S. nepalensis* strain CNDG and preparation of peptidase fractions. a We hypothesized that corisin or corisin-like peptides are cleaved from transglycosylase by a protease secreted by the bacterium into the culture supernatant. **b** *S. nepalensis* culture supernatant was fractionated using a >50 kDa filter and the resulting flow-through concentrated fraction using >10 kDa filters. The >10 kDa-concentrated sample was loaded onto a Sephacryl S-300 column and separated into several fractions. The proteolytic products were resolved by sodium dodecyl sulfate-polyacrylamide gel electrophoresis and silver staining. **c** Fraction numbers 10 to 13 were pooled and concentrated to 0.5 ml. **d** The concentrated sample was loaded onto a Sephacryl S-300 column, separated in several fractions, absorbance measured at 280 nm, and the proteolytic activity of each fraction on recombinant transglycosylase 351 assessed. S., *Staphylococcus.*

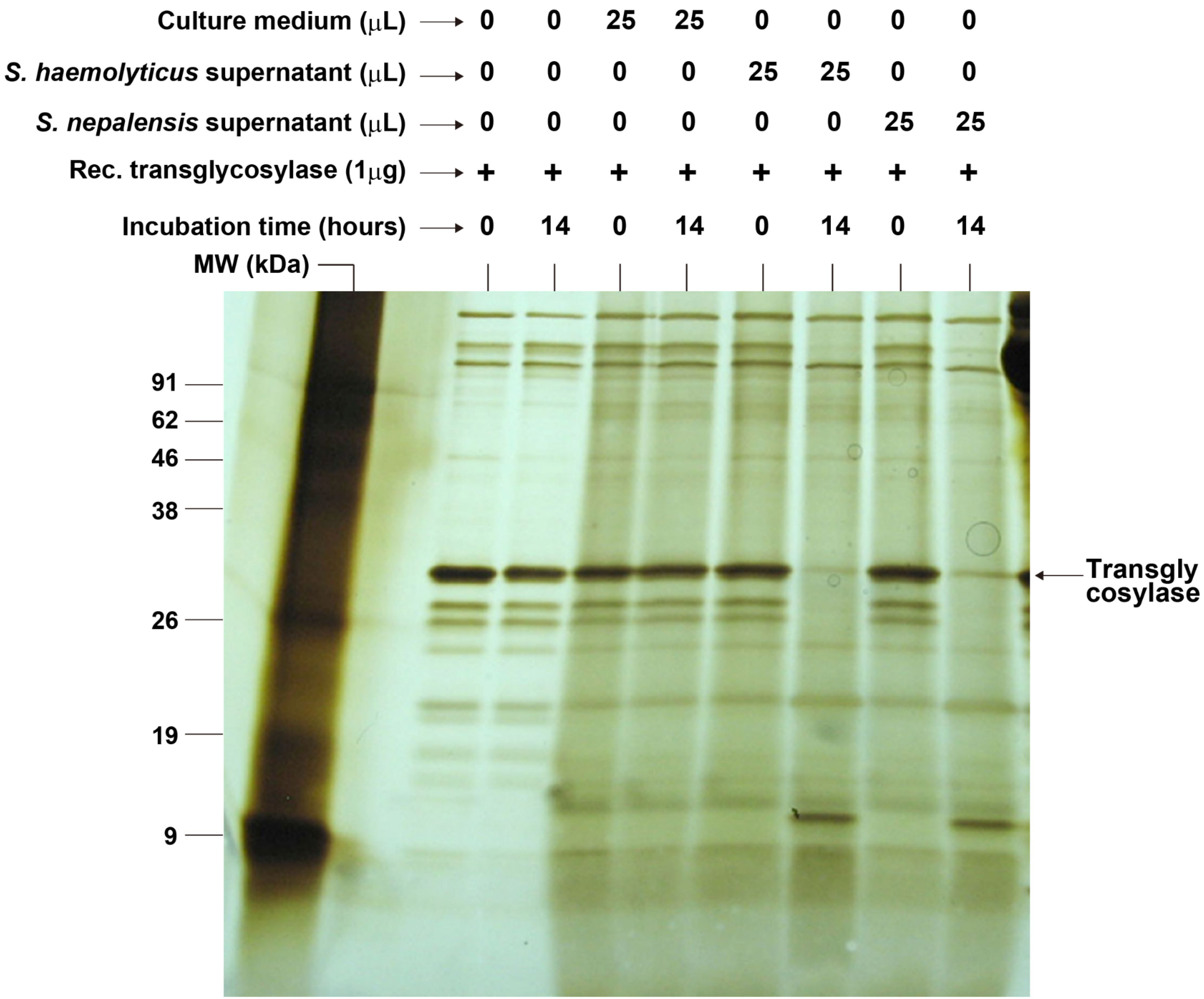

Culture medium (μL) 0 0 25 25 0 0 0 0
S. haemolyticus supernatant (μL) 0 0 0 0 25 25 0 0
S. nepalensis supernatant (μL) 0 0 0 0 0 0 25 25
Rec. transglycosylase (1μg) + + + + + + + +
Incubation time (hours) 0 14 0 14 0 14 0 14
MW (kDa)
91
62
46
38
26
19
9
Transgly cosylase

Supplementary Fig. 10. The culture supernatant from *Staphylococcus haemolyticus* degrades transglycosylase. A sodium dodecyl sulfate-polyacrylamide gel stained with silver staining after completing the electrophoresis of a reaction mixture containing digestion buffer, recombinant transglycosylase from *Staphylococcus nepalensis,* and culture supernatant (>10 kDa fraction) of *Staphylococcus haemolyticus* strain 12 incubated at 37ºC for 14h. A representative image from two independent experiments with similar results are shown. The supernatant (>10 kDa fraction) of *Staphylococcus nepalensis* was used as the control.

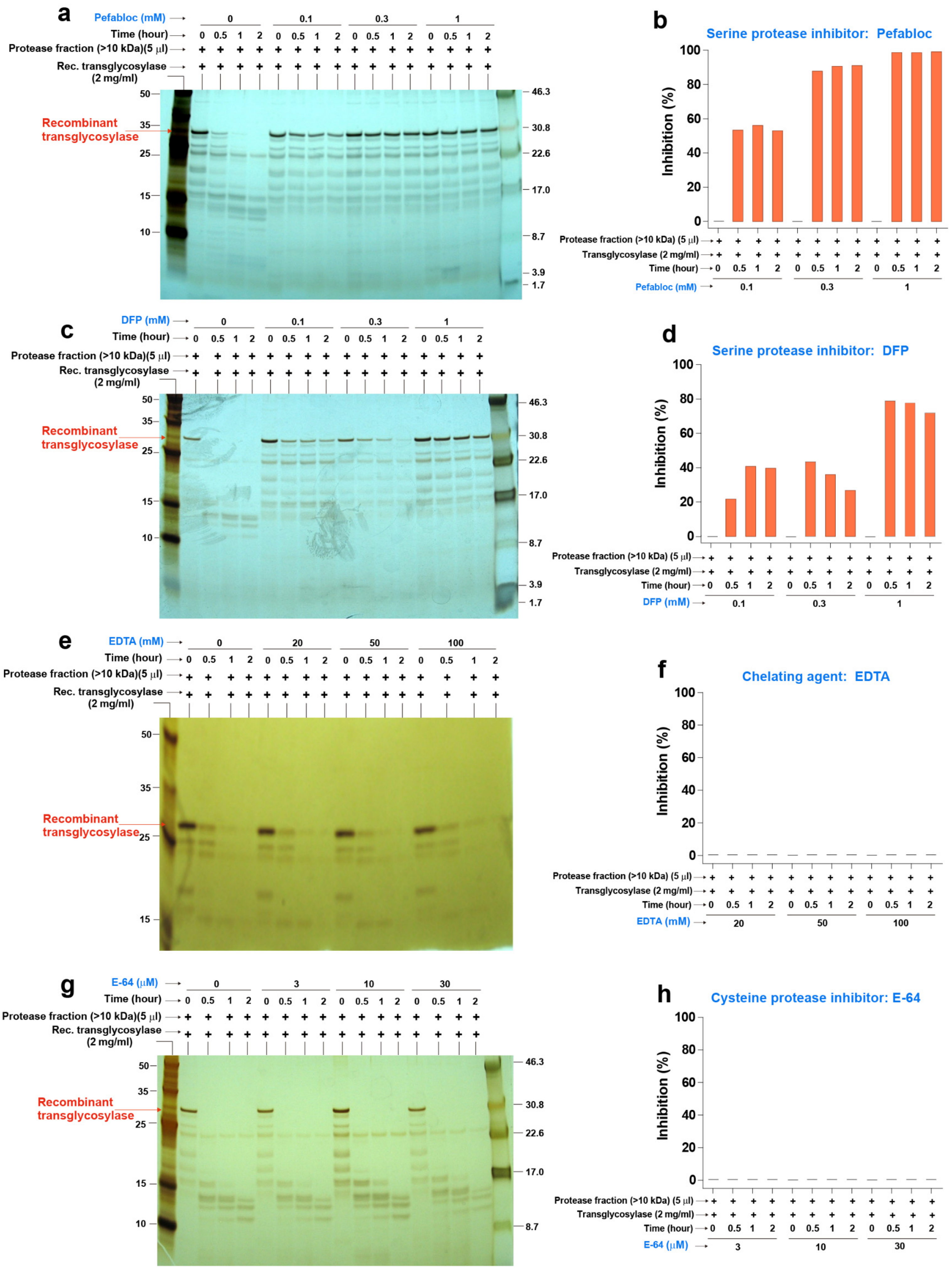

a
Pefabloc (mM)
0 0.1 0.3 1
Time (hour)
0 0.5 1 2 0 0.5 1 2 0 0.5 1 2 0 0.5 1 2
Protease fraction (>10 kDa)(5 μl)
Rec. transglycosylase (2 mg/ml)
Recombinant transglycosylase
50 35 25 15 10
46.3 30.8 22.6 17.0 8.7 3.9 1.7
b
Serine protease inhibitor: Pefabloc
Inhibition (%)
0 20 40 60 80 100
Protease fraction (>10 kDa) (5 μl)
Transglycosylase (2 mg/ml)
Time (hour)
Pefabloc (mM)
0.1 0.3 1
c
DFP (mM)
0 0.1 0.3 1
Time (hour)
Protease fraction (>10 kDa)(5 μl)
Rec. transglycosylase (2 mg/ml)
Recombinant transglycosylase
d
Serine protease inhibitor: DFP
Inhibition (%)
DFP (mM)
e
EDTA (mM)
0 20 50 100
Time (hour)
Protease fraction (>10 kDa)(5 μl)
Rec. transglycosylase (2 mg/ml)
Recombinant transglycosylase
f
Chelating agent: EDTA
Inhibition (%)
EDTA (mM)
20 50 100
g
E-64 (μM)
0 3 10 30
Time (hour)
Protease fraction (>10 kDa)(5 μl)
Rec. transglycosylase (2 mg/ml)
Recombinant transglycosylase
h
Cysteine protease inhibitor: E-64
Inhibition (%)
E-64 (μM)
3 10 30

Supplementary Fig. 11. A bacteria-derived putative serine protease cleaves transglycosylase. Reaction mixtures containing recombinant transglycosylase (2 mg/ml), protease fraction (>10 kDa) prepared from *Staphylococcus nepalensis* culture supernatant, varying concentrations (0.1, 0.3, 1 mM) of the serine protease inhibitor pefabloc SC (**a**, **b**) or diisopropyl fluorophosphate (DFP; **c**, **d**), the chelating agent ethylenediaminetetraacetic acid (EDTA; **e**, **f**) or the cysteine protease inhibitor E-64 (**g, h**) in a digestion buffer. Each mixture was incubated for 0.5, 1, and 2h before running on a sodium dodecyl sulfate-polyacrylamide gel electrophoresis and performing silver staining. Representative images from two independent experiments with similar results are shown. Rec, recombinant. The source data underlying **b**, **d**, **f**, **h** are provided in the Source Data file.

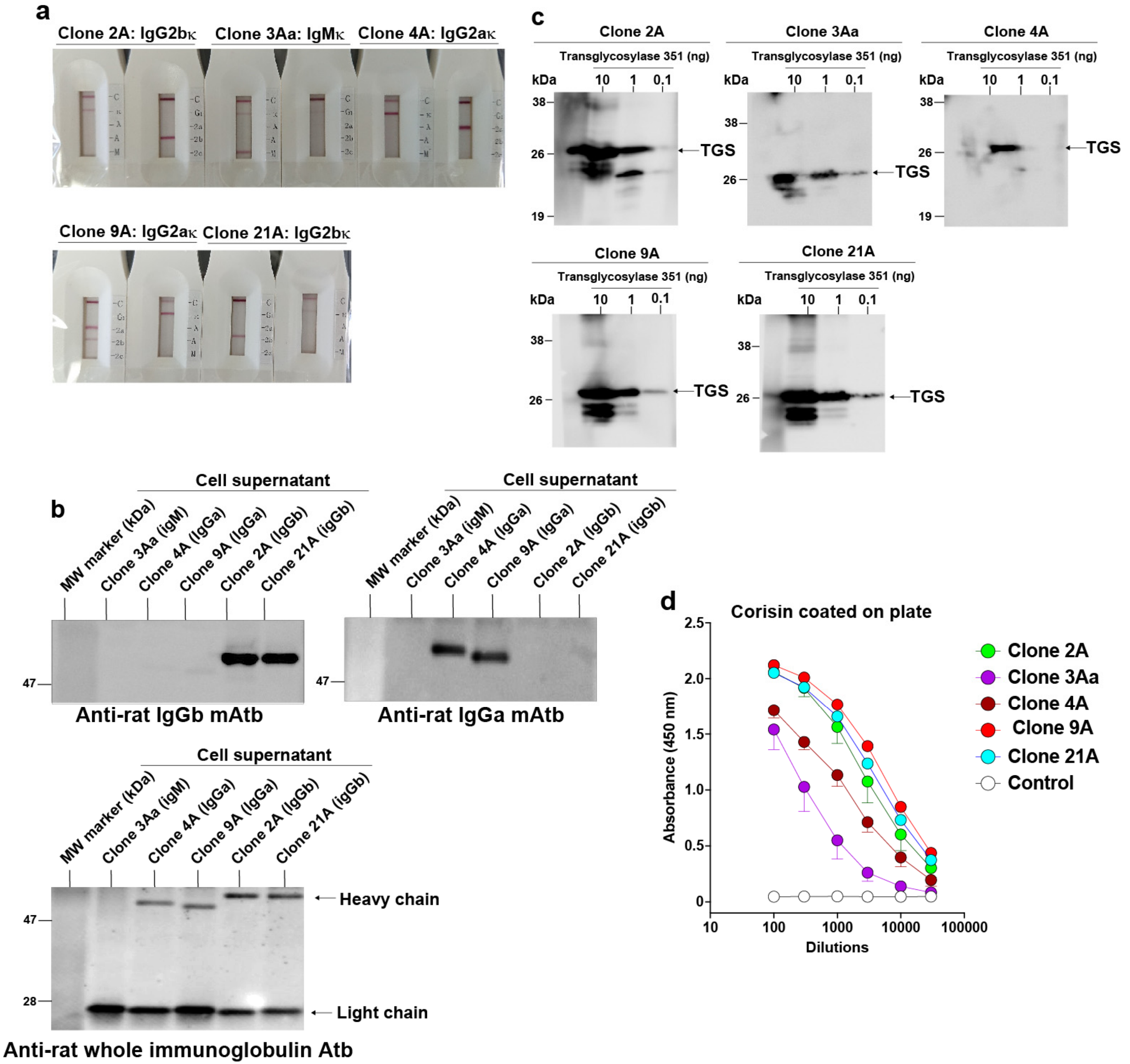
a
Clone 2A: IgG2bκ
Clone 3Aa: IgMκ
Clone 4A: IgG2aκ
Clone 9A: IgG2aκ
Clone 21A: IgG2bκ
b
Cell supernatant
MW marker (kDa)
Clone 3Aa (igM)
Clone 4A (IgGa)
Clone 9A (IgGa)
Clone 2A (IgGb)
Clone 21A (igGb)
47
Anti-rat IgGb mAtb
Anti-rat IgGa mAtb
Heavy chain
Light chain
28
Anti-rat whole immunoglobulin Atb
c
Clone 2A
Clone 3Aa
Clone 4A
Clone 9A
Clone 21A
Transglycosylase 351 (ng)
kDa
10
1
0.1
38
26
19
TGS
d
Corisin coated on plate
Absorbance (450 nm)
Dilutions
Clone 2A
Clone 3Aa
Clone 4A
Clone 9A
Clone 21A
Control

Supplementary Fig. 12. Different isotypes of the anticorisin monoclonal antibody bind to the corisin peptide and corisin sequence on the transglycosylase 351. a Determination of the isotype of each monoclonal antibody was performed using Rapid Monoclonal Antibody Isotyping Kit as described under Methods. **b** Western blotting of each clone supernatant using anti-rat IgGa or anti-rat IgGb monoclonal antibody (mAtb) or anti-rat IgM, IgG polyclonal antibody. Representative blots from three independent experiments with similar results are shown. **c** Varying amounts of the recombinant transglycosylase 351 were electrophoresed on a polyacrylamide gel, and Western blotting was performed using 1:1000 dilution of the supernatant of hybridomas producing each clone of anticorisin monoclonal antibody. The smaller bands under 26 kDa molecular weight markers are degraded products of the transglycosylase 351, which is known to degrade during recombinant protein purification. Representative blots from two independent experiments with similar results are shown. **d** The corisin peptide was coated on a microplate, and binding of each clone of the anticorisin mono-clonal antibody was evaluated using varying dilutions of the supernatant of the hybridomas. N=3 in each group. Data are expressed as the mean ± S.D. TGS, transglycosylase. The source data underlying **d** are provided in the Source Data file.

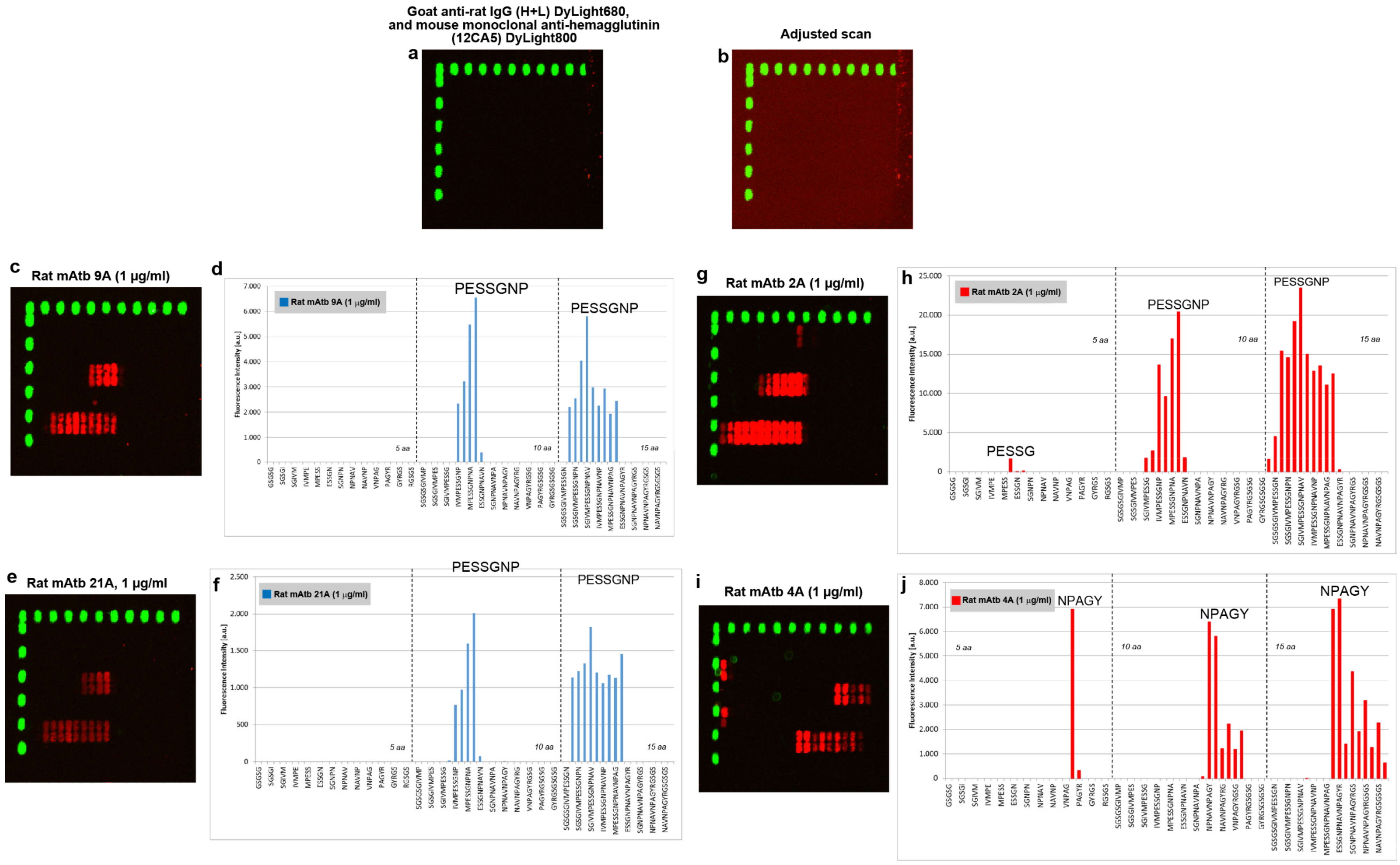
Goat anti-rat IgG (H+L) DyLight680, and mouse monoclonal anti-hemagglutinin (12CA5) DyLight800
a
Adjusted scan
b
c
Rat mAtb 9A (1 µg/ml)
d
Rat mAtb 9A (1 µg/ml)
PESSGNP
PESSGNP
Fluorescence Intensity [a.u.]
5 aa
10 aa
15 aa
g
Rat mAtb 2A (1 µg/ml)
h
Rat mAtb 2A (1 µg/ml)
PESSG
PESSGNP
PESSGNP
Fluorescence Intensity [a.u.]
5 aa
10 aa
15 aa
e
Rat mAtb 21A, 1 µg/ml
f
Rat mAtb 21A (1 µg/ml)
PESSGNP
PESSGNP
Fluorescence Intensity [a.u.]
5 aa
10 aa
15 aa
i
Rat mAtb 4A (1 µg/ml)
j
Rat mAtb 4A (1 µg/ml)
NPAGY
NPAGY
NPAGY
Fluorescence Intensity [a.u.]
5 aa
10 aa
15 aa

Supplementary Fig. 13. Rat anticorisin monoclonal antibodies bind to a consensus motif PESSGNP or NPAGY in corisin. Mapping was performed at PEPperPRINT Inc. (Heidelberg; Germany). Pre-staining of a peptide microarray copy was done with the secondary (goat anti-rat IgG [H+L] DyLight680) and control (mouse monoclonal anti-HA [12CA5] DyLight800) antibodies as described under Methods. The control staining gave rise to the expected well-defined hemagglutinin control spot pattern and validated the overall peptide microarray integrity and assay quality (**a**, **b**). Subsequent incubation of other peptide microarray copies with the rat monoclonal antibodies 9A (**c, d**), 21A (**e**, **f**), 2A (**g**, **h**), and 4A (**i**, **j**) at a concentration of 1 µg/ml in incubation buffer was followed by staining with secondary and control antibodies as well as read-out at scanning intensities of 7/7 (red/green) using the LI-COR Odyssey Imaging System. The source data underlying **d**, **f**, **h**, and **j** are available in https://zenodo.org/record/5803063#.YcWY-WDP2ck.

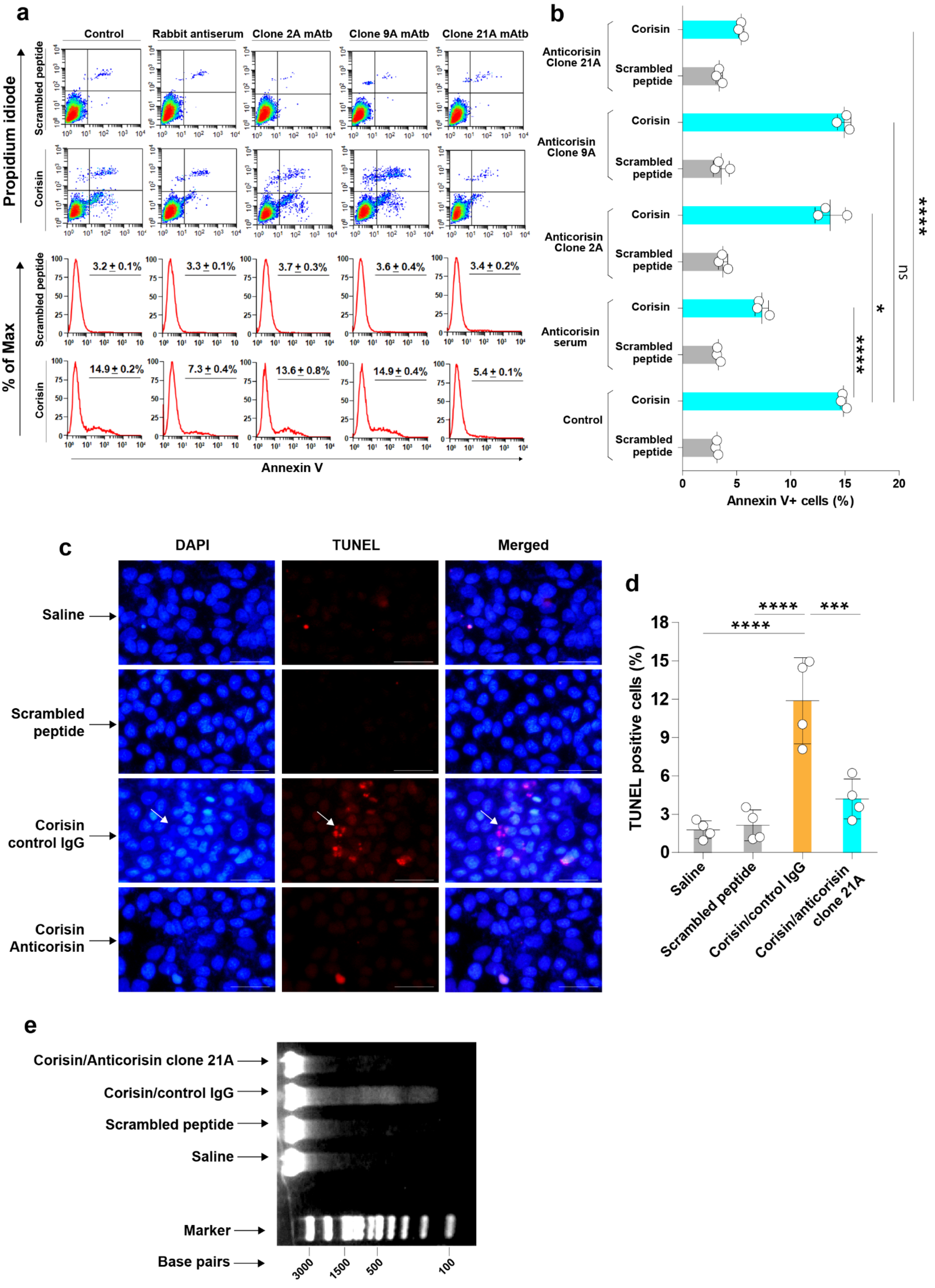
a
Control
Rabbit antiserum
Clone 2A mAtb
Clone 9A mAtb
Clone 21A mAtb
Propidium idiode
Scrambled peptide
Corisin
% of Max
3.2 ± 0.1%
3.3 ± 0.1%
3.7 ± 0.3%
3.6 ± 0.4%
3.4 ± 0.2%
14.9 ± 0.2%
7.3 ± 0.4%
13.6 ± 0.8%
14.9 ± 0.4%
5.4 ± 0.1%
Annexin V
b
Anticorisin Clone 21A
Anticorisin Clone 9A
Anticorisin Clone 2A
Anticorisin serum
Control
Corisin
Scrambled peptide
****
ns
*
****
Annexin V+ cells (%)
c
DAPI
TUNEL
Merged
Saline
Scrambled peptide
Corisin control IgG
Corisin Anticorisin
d
TUNEL positive cells (%)
****
****
***
Saline
Scrambled peptide
Corisin/control IgG
Corisin/anticorisin clone 21A
e
Corisin/Anticorisin clone 21A
Corisin/control IgG
Scrambled peptide
Saline
Marker
Base pairs
3000
1500
500
100

Supplementary Fig. 14. The anticorisin monoclonal antibody clone 21A shows potent neutralizing activity on corisin-induced apoptosis of human alveolar epithelial cells. a A549 alveolar epithelial cells were cultured in the presence of corisin (10 μg/ml) or scrambled peptide (10 μg/ml) after pretreating with each clone (hybridoma supernatant at 1:100 dilution) of the anticorisin monoclonal antibody. A549 cells pretreated with rat anticorisin serum were used as positive controls, and cells without pretreatment were used as the negative control. **b** The percentage of apoptotic cells was determined by flow cytometry and quantified. N=3 in each group. Data are expressed as the mean ± S.D. Statistical analysis was performed ANOVA with a post hoc Newman-Keuls test. *p<0.05; ****p<0.0001. **c**, **d** A549 cells were cultured in the presence of saline, scrambled peptide (10 μg/ml), corisin (10 μg/ml) with control IgG (20 μg/ml) or corisin with anticorisin mAtb clone 21A (20 μg/ml) for 72 hours and DNA fragmentation was evaluated by staining with terminal deoxynucleotidyltransferase dUTP Nick-End Labeling (TUNEL) using a commercial kit following the manufacturer's instructions and the number of TUNEL positive cells was counted. Representative lung microphotograph of each group is shown. Arrows show TUNEL positive cells. Representative images from two independent experiments with similar results are shown. Scale bars indicate 50 µm. Bars indicate the means ± S.D. Statistical analysis by ANOVA with a post hoc Newman-Keuls test. ***p<0.001; ****p<0.0001. **e** A549 cells were cultured in the presence of saline, scrambled peptide (10 μg/ml), corisin (10 μg/ml) with control IgG or corisin with anticorisin mAtb clone 21A (20 μg/ml) for 72 hours and DNA laddering was evaluated. DNA was extracted using a commercial kit following the manufacturer's instructions. A representative image from three independent experiments with similar results are shown. The source data underlying **b**, and **d** are provided in the Source Data file.

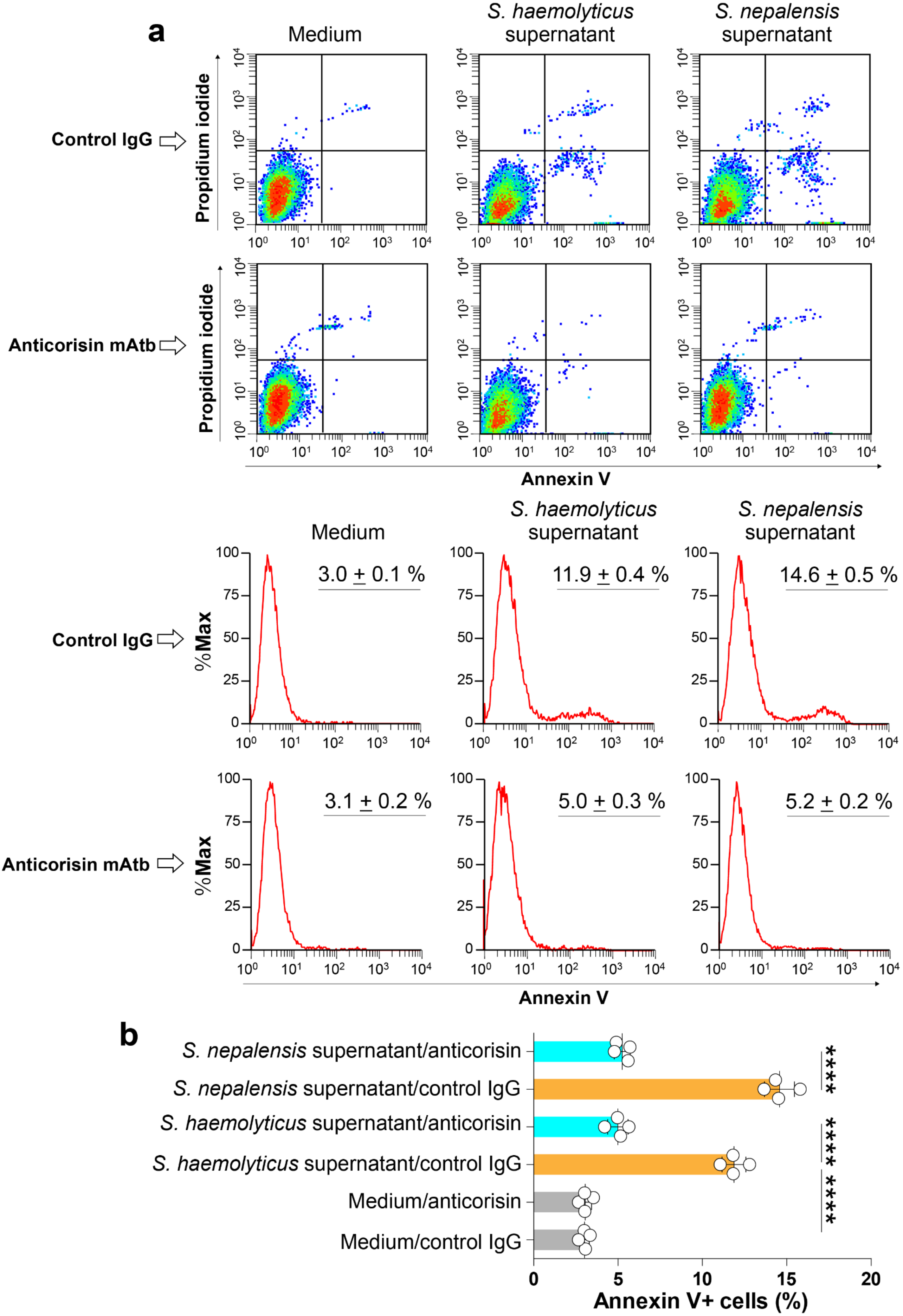

a
Medium
S. haemolyticus supernatant
S. nepalensis supernatant
Control IgG
Anticorisin mAtb
Propidium iodide
Annexin V
%Max
3.0 ± 0.1 %
11.9 ± 0.4 %
14.6 ± 0.5 %
3.1 ± 0.2 %
5.0 ± 0.3 %
5.2 ± 0.2 %
b
S. nepalensis supernatant/anticorisin
S. nepalensis supernatant/control IgG
S. haemolyticus supernatant/anticorisin
S. haemolyticus supernatant/control IgG
Medium/anticorisin
Medium/control IgG
****
****
****
Annexin V+ cells (%)

Supplementary Fig. 15. The anticorisin monoclonal antibody clone 21A neutralizes the apoptotic activity of the culture supernatant from *Staphylococcus haemolyticus* on human alveolar epithelial cells. a A549 alveolar epithelial cells were cultured in the presence of the culture supernatant from *Staphylococcus haemolyticus* strain 12 after pretreating with the hybridoma supernatant (at 1:100 dilution) of the anticorisin monoclonal antibody clone 21A. A549 cells pretreated with the monoclonal antibody 21A or control IgG alone were the negative controls. Cells pretreated with the supernatant from *Staphylococcus nepalensis* were the positive controls. **b** The percentage of apoptotic cells was determined by flow cytometry and quantified. N=4 in each group. Data are expressed as the mean ± S.D. Statistical analysis was performed using ANOVA with a post hoc Newman-Keuls test. ****$p<0.0001$. S., *Staphylococcus*. The source data underlying **b** are provided in the Source Data file.

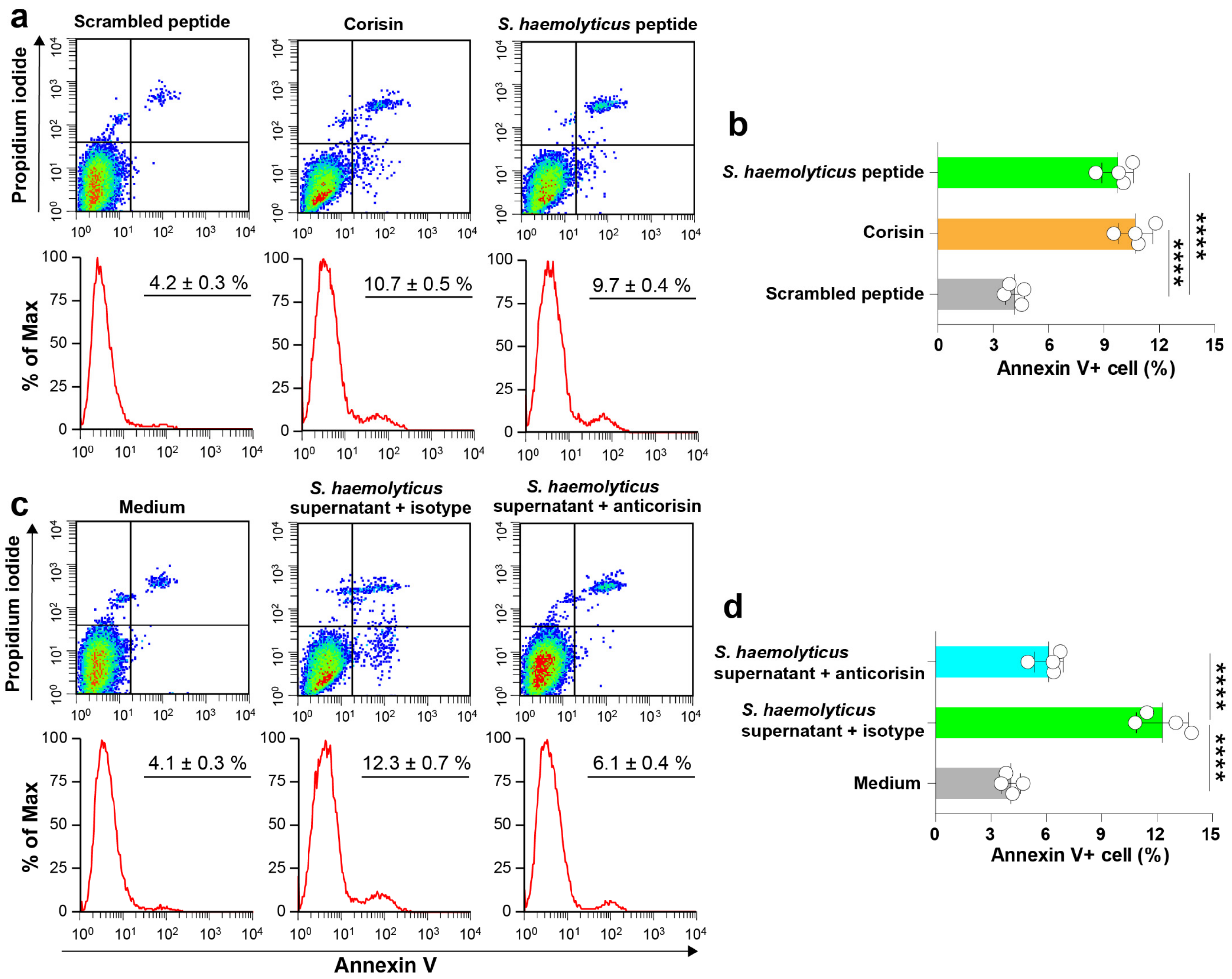

a
Scrambled peptide
Corisin
S. haemolyticus peptide
Propidium iodide
% of Max
4.2 ± 0.3 %
10.7 ± 0.5 %
9.7 ± 0.4 %
b
S. haemolyticus peptide
Corisin
Scrambled peptide
****
****
Annexin V+ cell (%)
c
Medium
S. haemolyticus supernatant + isotype
S. haemolyticus supernatant + anticorisin
4.1 ± 0.3 %
12.3 ± 0.7 %
6.1 ± 0.4 %
d
S. haemolyticus supernatant + anticorisin
S. haemolyticus supernatant + isotype
Medium
Annexin V+ cell (%)
Annexin V

Supplementary Fig. 16. The corisin-like peptide from *Staphylococcus haemolyticus* induces apoptosis and anticorisin mAtb clone 21A neutralizes the apoptotic activity of the culture supernatant's apoptotic activity in normal human bronchial epithelial cells. a, **b** Normal human bronchial epithelial (NHBE) cells were cultured in the presence of the corisin-like synthetic peptide from *Staphylococcus haemolyticus* strain 12, corisin, or scrambled peptide for 24h, and cell apoptosis was analyzed by flow cytometry. N=4 in each group. Bars indicate the mean ± S.D. Statistical analysis by ANOVA with a post hoc Newman-Keuls test. ***p<0.001. **c**, **d** NHBE cells were cultured in the presence of the culture supernatant (1/10) from *Staphylococcus haemolyticus* strain 12 after pretreating with the hybridoma supernatant (at 1:100 dilution) of the anticorisin mAtb clone 21A and cell apoptosis was evaluated by flow cytometry. NHBE cells cultured in the presence of the bacterial supernatant pretreated with the control IgG were the negative controls. N=4 in each group. Data are expressed as the mean ± S.D. Statistical analysis by ANOVA with a post hoc Newman-Keuls test. ****p<0.0001. The source data underlying **b** and **d** are provided in the Source Data file. S., *Staphylococcus*.

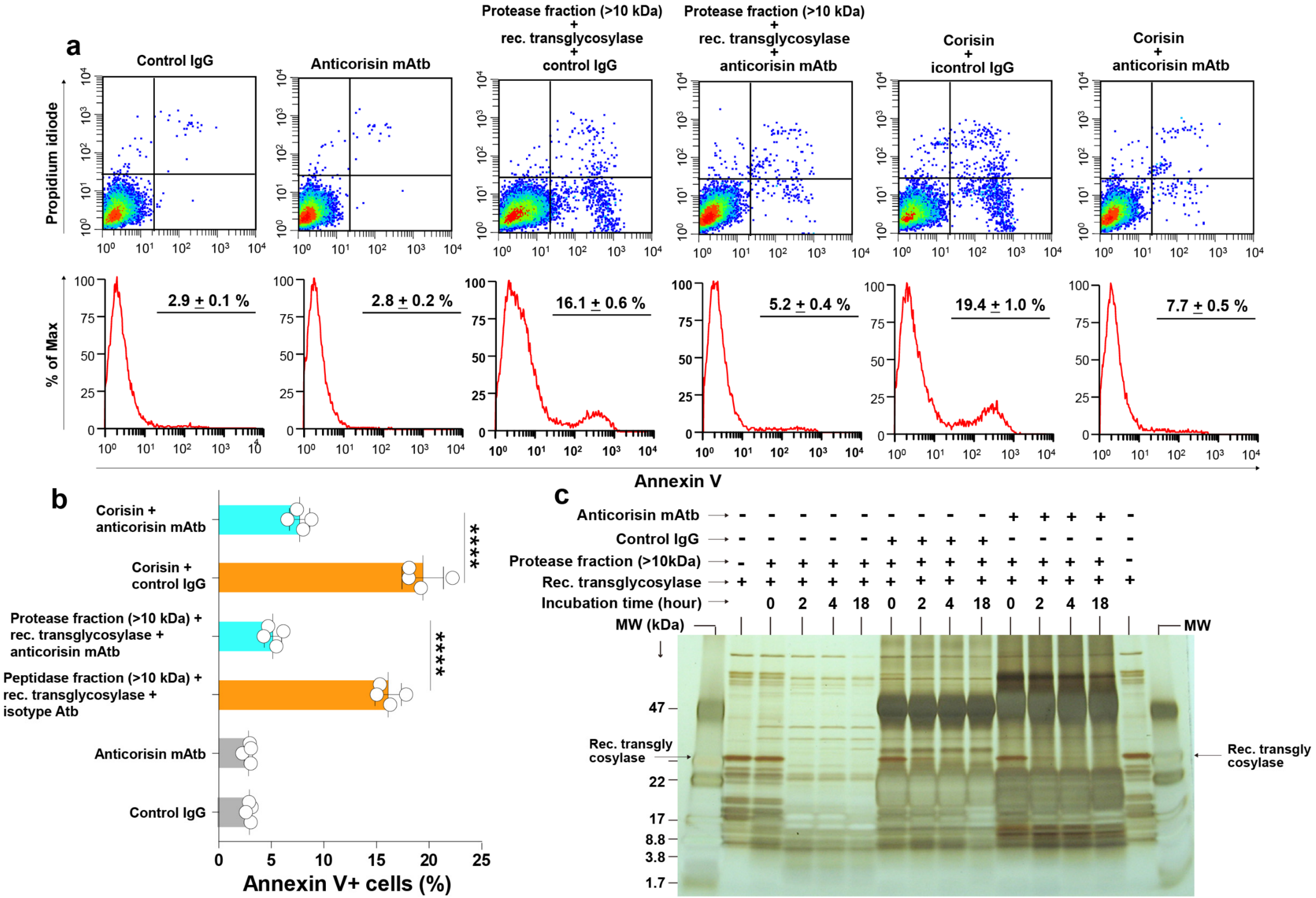
a
Control IgG
Anticorisin mAtb
Protease fraction (>10 kDa) + rec. transglycosylase + control IgG
Protease fraction (>10 kDa) + rec. transglycosylase + anticorisin mAtb
Corisin + icontrol IgG
Corisin + anticorisin mAtb
Propidium idiode
% of Max
2.9 ± 0.1 %
2.8 ± 0.2 %
16.1 ± 0.6 %
5.2 ± 0.4 %
19.4 ± 1.0 %
7.7 ± 0.5 %
Annexin V
b
Corisin + anticorisin mAtb
Corisin + control IgG
Protease fraction (>10 kDa) + rec. transglycosylase + anticorisin mAtb
Peptidase fraction (>10 kDa) + rec. transglycosylase + isotype Atb
Anticorisin mAtb
Control IgG
****
****
Annexin V+ cells (%)
c
Anticorisin mAtb
Control IgG
Protease fraction (>10kDa)
Rec. transglycosylase
Incubation time (hour)
0 2 4 18 0 2 4 18 0 2 4 18
MW (kDa)
MW
47
22
17
8.8
3.8
1.7
Rec. transgly cosylase
Rec. transgly cosylase

Supplementary Fig. 17. Anticorisin neutralizing monoclonal antibody inhibits apoptosis of lung cells induced by transglycosylase degradation products. a, **b** A reaction mixture containing recombinant transglycosylase (2 mg/ml), protease fraction (>10 kDa) *Staphylococcus nepalensis* culture supernatant, and 20 µg/ml of anticorisin mAtb clone 21A or control IgG were incubated for 2h and then added to a culture of A549 cells. After 48h of culture, apoptosis was evaluated by flow cytometry analysis. Cells treated with corisin plus anticorisin mAtb or control IgG were used as positive controls and cells treated with anticorisin mAtb or control IgG alone were used as negative controls. The percentage of apoptotic cells was determined by flow cytometry. N=4 in each group. Data are expressed as the mean ± S.D. Statistical analysis was performed using ANOVA with a post hoc Newman-Keuls test. ****p<0.0001. **c** Degradation of recombinant transglycosylase was evaluated overtime (0, 2, 4, and 18h), and then the reaction mixture was run on a sodium dodecyl sulfate-polyacrylamide gel electrophoresis and stained with silver staining. A representative image from two experiments with similar results are shown. The source data underlying **b** are provided in the Source Data file.

Supplementary Table 1. Profile of IPF patients

Clinical parameters	No of patients and mean values
No of Japanese patients	36
Sex	
Male	34
Female	2
Age (years-old)	66.00 ± 8.43
Lung function test	
VC (L)	2.53 ± 0.67
VC (% predicted)	76.10 ± 17.57
FVC (L)	2.51 ± 0.67
FVC (% predicted)	77.80 ± 19.53
FEV1 (L)	2.04 ± 0.49
FEV1/FVC (%)	82.38 ± 8.21

Data are the mean ± S.D. IPF, idiopathic pulmonary fibrosis; VC, vital capacity; FEV1, forced expiratory volume in one second; FVC, forced volume vital capacity; L, liters. Source data are provided in the Source Data file.

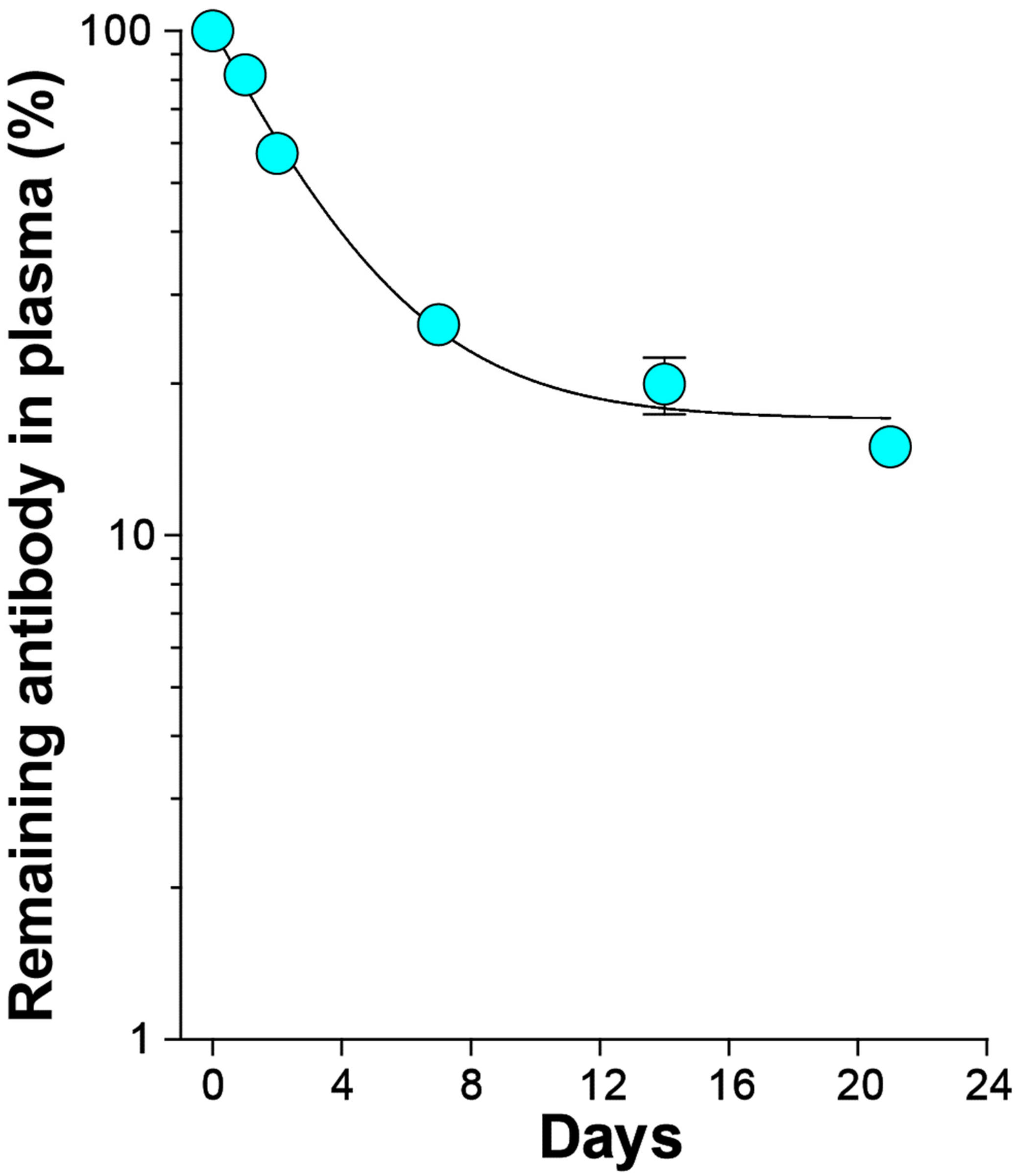

Supplementary Fig. 18. The average half-life of the anticorisin neutralizing mAtb in mouse blood. Female TGFβ1 transgenic mice (n=5) without fibrosis with the same genetic background (C57BL/6J) as the wild-type mice were used in the experiment. Mice (n=5) received an intraperitoneal injection of (20 mg/kg) anticorisin mAtb (clone 21A), and blood was sampled after 3, 6, 24, 48 168, 336, and 504 hours. Plasma was separated after centrifugation to measure the levels of anticorisin antibody using an enzyme immunoassay as described under Methods. Data are expressed as the mean ± standard error of the mean. The half-life of the anticorisin neutralizing mAtb was calculated using an exponential decay equation model available in the GraphPad Prism version 7. The source data underlying this figure are provided in the Source Data file.

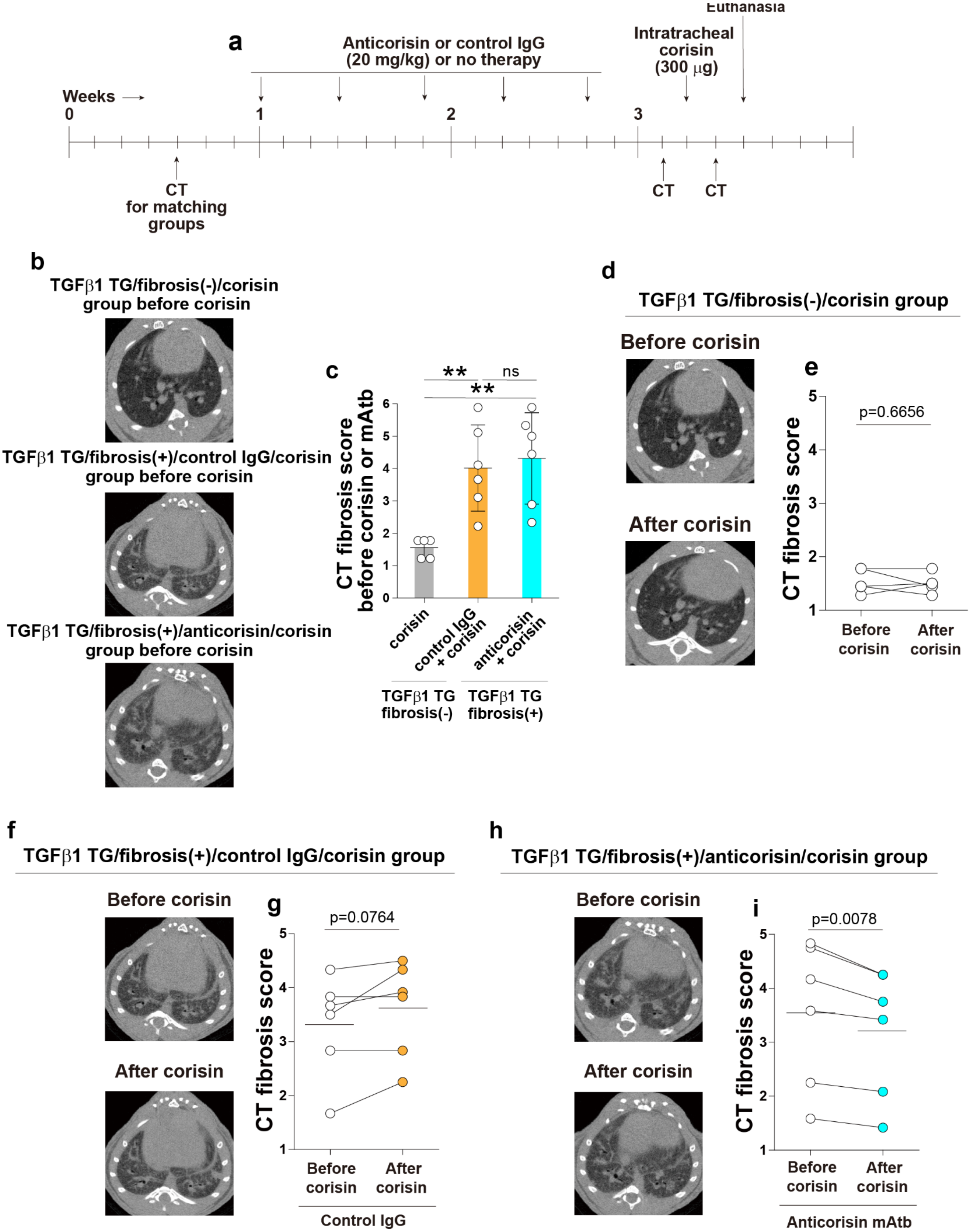

a
Anticorisin or control IgG (20 mg/kg) or no therapy
Intratracheal corisin (300 μg)
Euthanasia
Weeks
0
1
2
3
CT for matching groups
CT
CT
b
TGFβ1 TG/fibrosis(-)/corisin group before corisin
TGFβ1 TG/fibrosis(+)/control IgG/corisin group before corisin
TGFβ1 TG/fibrosis(+)/anticorisin/corisin group before corisin
c
CT fibrosis score before corisin or mAtb
**
ns
**
corisin
control IgG + corisin
anticorisin + corisin
TGFβ1 TG fibrosis(-)
TGFβ1 TG fibrosis(+)
d
TGFβ1 TG/fibrosis(-)/corisin group
Before corisin
After corisin
e
p=0.6656
CT fibrosis score
Before corisin
After corisin
f
TGFβ1 TG/fibrosis(+)/control IgG/corisin group
Before corisin
After corisin
g
p=0.0764
CT fibrosis score
Before corisin
After corisin
Control IgG
h
TGFβ1 TG/fibrosis(+)/anticorisin/corisin group
Before corisin
After corisin
i
p=0.0078
CT fibrosis score
Before corisin
After corisin
Anticorisin mAtb

Supplementary Fig. 19. Monoclonal anticorisin antibody prevents deterioration of lung radiological findings in TGFβ1 TG mice. a, **b**, **c** Computed tomography (CT) was performed before starting treatment with an anticorisin monoclonal antibody (mAtb) or control IgG. The radiological findings of lung fibrosis were evaluated using a CT fibrosis score as described under Methods, and the TGFβ1 TG mice were randomly allocated into two groups with a matched grade of lung fibrosis and one group without lung fibrosis. A group of TGFβ1 TG mice (n=6) with lung fibrosis received an intraperitoneal injection of anticorisin mAtb (TGFβ1 TG/fibrosis(+)/anticorisin/corisin) and another group (n=6) with lung fibrosis received control IgG (TGFβ1 TG/fibrosis(+)/control IgG/corisin) five times every two days before intratracheal instillation of corisin. The group of TGFβ1 TG mice without fibrosis (n=5) (TGFβ1 TG/fibrosis(-)/corisin) received only intratracheal corisin. Representative CT finding in each group is shown. Data are expressed as the mean ± S.D. Statistical analysis was performed using ANOVA with a post hoc Newman-Keuls test. **p<0.01. ns, not significant. **d**, **e**, **f**, **g**, **h**, **i** Computed tomography (CT) was performed before and after the administration of corisin for comparative study. Representative CT findings before and after treatment in each group (n=6) are shown. Bars indicate the mean values. Statistical analysis was performed by a two-sided paired t-test. TGFβ1, transforming growth factor β1; TG, transgenic; ns, not significant. The source data underlying **c**, **e**, **g**, and **i** are provided in the Source Data file.

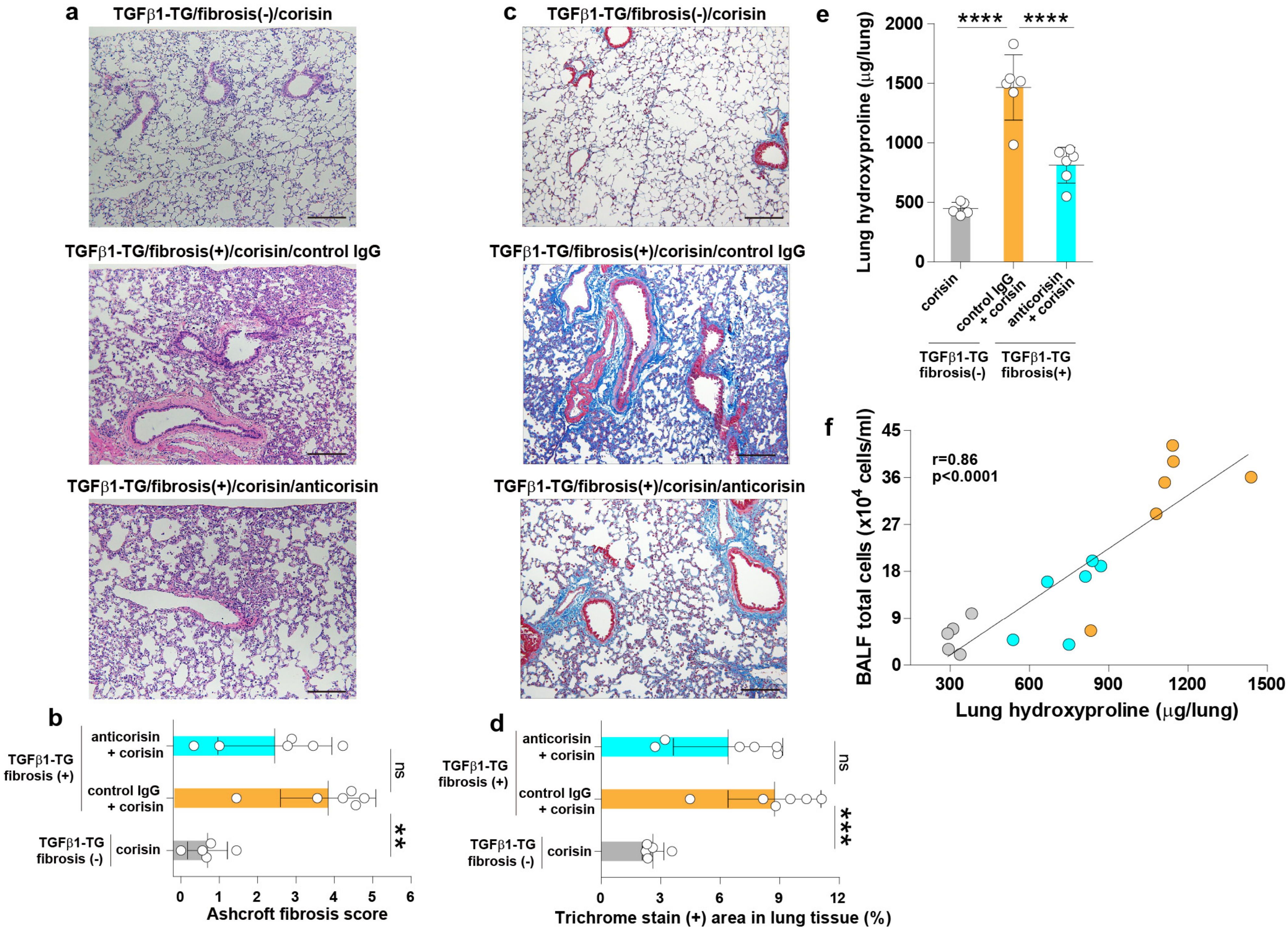
a
TGFβ1-TG/fibrosis(-)/corisin
TGFβ1-TG/fibrosis(+)/corisin/control IgG
TGFβ1-TG/fibrosis(+)/corisin/anticorisin
b
TGFβ1-TG fibrosis (+)
anticorisin + corisin
control IgG + corisin
TGFβ1-TG fibrosis (-)
corisin
ns
**
0 1 2 3 4 5 6
Ashcroft fibrosis score
c
TGFβ1-TG/fibrosis(-)/corisin
TGFβ1-TG/fibrosis(+)/corisin/control IgG
TGFβ1-TG/fibrosis(+)/corisin/anticorisin
d
TGFβ1-TG fibrosis (+)
anticorisin + corisin
control IgG + corisin
TGFβ1-TG fibrosis (-)
corisin
ns
***
0 3 6 9 12
Trichrome stain (+) area in lung tissue (%)
e
****
****
Lung hydroxyproline (μg/lung)
0 500 1000 1500 2000
corisin
control IgG + corisin
anticorisin + corisin
TGFβ1-TG fibrosis(-)
TGFβ1-TG fibrosis(+)
f
r=0.86
p<0.0001
BALF total cells (x10^4 cells/ml)
0 9 18 27 36 45
300 600 900 1200 1500
Lung hydroxyproline (μg/lung)

Supplementary Fig. 20. Monoclonal anticorisin antibody inhibits acute exacerbation of pulmonary fibrosis in TGFβ1 TG mice. TGFβ1 TG mice were randomly allocated into two groups with a matched grade of lung fibrosis and one group without lung fibrosis. A group of TGFβ1 TG mice (n=6) with lung fibrosis received an intraperitoneal injection of anticorisin monoclonal antibody (mAtb), and another group (n=6) with lung fibrosis received control IgG five times every two days before intratracheal instillation of corisin. The group of TGFβ1 TG mice without fibrosis (n=5) received only intratracheal corisin. After euthanasia by an overdose of anesthesia, bronchoalveolar lavage fluid (BALF) and the lungs were drawn from mice of each group. **a**, **b** The grade of lung fibrosis was evaluated by the Ashcroft fibrosis score as described under Methods. Representative lung microphotograph of each group is shown. Scale bars indicate 200 μm. Data are the mean ± S.D. Statistical analysis was performed by ANOVA with a post hoc Newman-Keuls test. **p<0.01. ns, not significant (p=0.1). **c**, **d** Lung collagen deposition was evaluated by Masson's trichrome staining, and the percentage of trichrome stain (+) area was measured using the WinRoof Image Processing Software. Representative lung microphotograph of each group is shown. Scale bars indicate 200 μm. Data are the mean ± S.D. Statistical analysis was performed by ANOVA with a post hoc Newman-Keuls test. ***p<0.001. ns, not significant (p=0.1). **e** The level of hydroxyproline was measured using a commercially available colorimetric assay kit following the manufacturer's instructions. Data are the mean ± S.D. Statistical analysis was performed by ANOVA with a post hoc Newman-Keuls test. ****p<0.0001. **f** Correlation between the lung hydroxyproline content and the total number of inflammatory cells described in **Figure 5b**. Statistical analysis by Spearman's correlation. TGFβ1, transforming growth factor β1; TG, transgenic. The source data underlying **b**, **d**, and **e** are provided in the Source Data file.

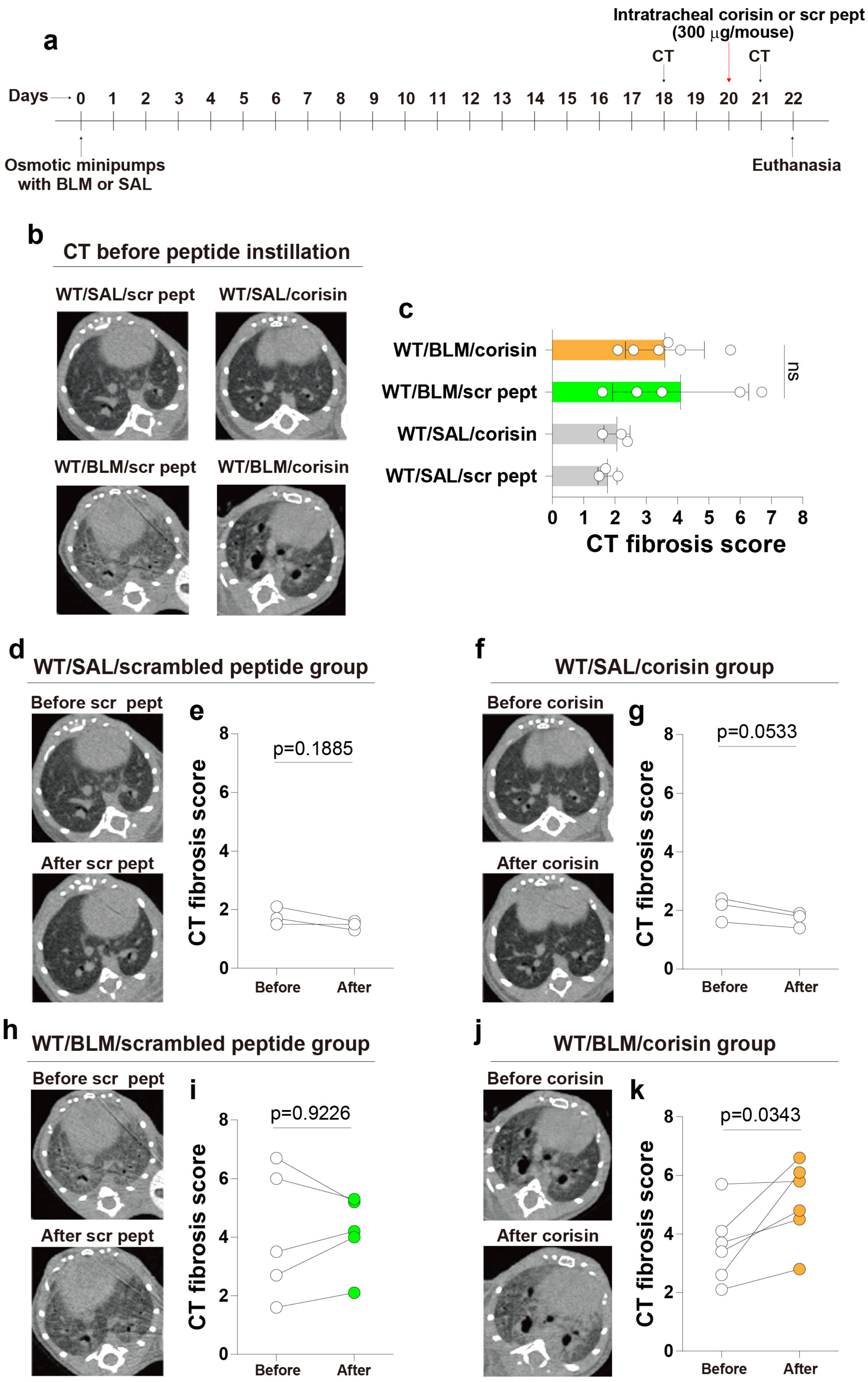

a
Intratracheal corisin or scr pept
(300 μg/mouse)
CT
CT
Days
0 1 2 3 4 5 6 7 8 9 10 11 12 13 14 15 16 17 18 19 20 21 22
Osmotic minipumps
with BLM or SAL
Euthanasia
b
CT before peptide instillation
WT/SAL/scr pept
WT/SAL/corisin
WT/BLM/scr pept
WT/BLM/corisin
c
WT/BLM/corisin
WT/BLM/scr pept
WT/SAL/corisin
WT/SAL/scr pept
ns
CT fibrosis score
d
WT/SAL/scrambled peptide group
Before scr pept
After scr pept
e
p=0.1885
CT fibrosis score
Before
After
f
WT/SAL/corisin group
Before corisin
After corisin
g
p=0.0533
CT fibrosis score
Before
After
h
WT/BLM/scrambled peptide group
Before scr pept
After scr pept
i
p=0.9226
CT fibrosis score
Before
After
j
WT/BLM/corisin group
Before corisin
After corisin
k
p=0.0343
CT fibrosis score
Before
After

Supplementary Fig. 21. Deterioration of lung radiological findings in mice with bleomycin-induced pulmonary fibrosis after intratracheal instillation of corisin. a Wild-type (WT) received bleomycin (BLM) or saline (SAL) on day 0 through an osmotic minipump implanted subcutaneously on the mouse back. Mice received intratracheal instillation (75 µl) of corisin or scrambled peptide (scr pept) (300 µg/mouse) on day 20 after pump implantation. Computed tomography (CT) was performed before and after the intratracheal administration of corisin or scrambled peptide before mouse euthanasia. Mice receiving SAL through osmotic minipumps and intra-tracheal corisin or scrambled peptide were used as controls. Mice were sacrificed on day 22 after BLM or SAL pump implantation. **b**, **c** Matched CT fibrosis score between groups receiving intratracheal instillation of corisin or scrambled peptide. The radiological findings of lung fibrosis were evaluated using a CT fibrosis score as described under Methods. The number of mice: n=5 in WT/BLM/scr pept, n=6 in WT/BLM/corisin groups, and n=3 in WT/SAL/scr pept and WT/SAL/corisin groups. Representative CT finding in each group is shown. Data are the mean ± S.D. Statistical analysis by ANOVA with a post hoc Newman-Keuls test. ns, not significant. **d, e, f, g h, c, j, k** The radiological findings of lung fibrosis were scored as described under Methods, and the scores before and after corisin or scrambled peptide instillation were compared in each group. The number of mice: n=5 in WT/BLM/scr pept, n=6 in WT/BLM/corisin groups, and n=3 in WT/SAL/scr pept and WT/SAL/corisin groups. Representative CT findings before and after intratracheal instillation in each group are shown. Statistical analysis by two-sided paired t-test. The source data underlying **c**, **e**, **g**, **i**, and **k** are provided in the Source Data file.

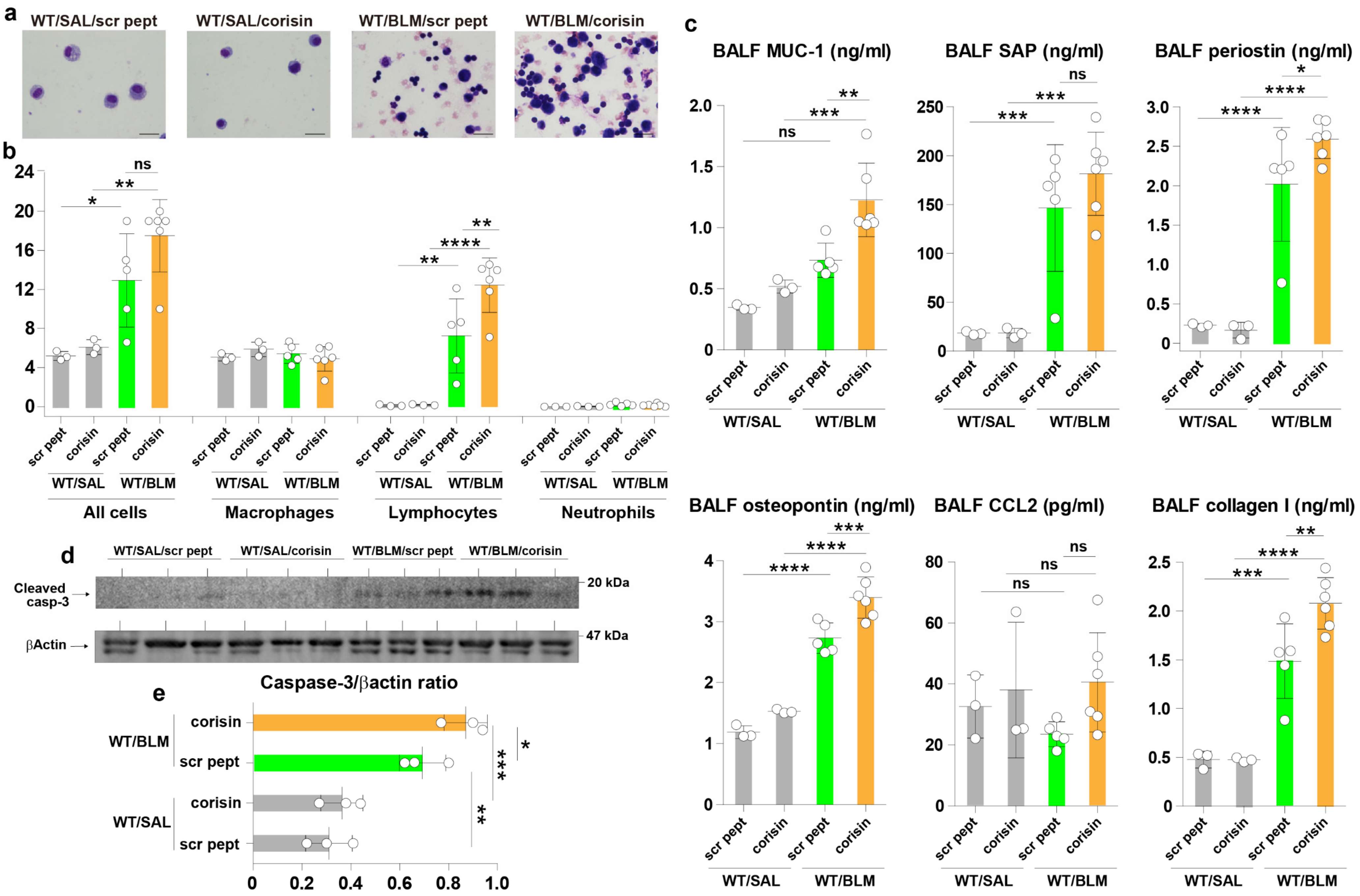

a
WT/SAL/scr pept
WT/SAL/corisin
WT/BLM/scr pept
WT/BLM/corisin
b
All cells
Macrophages
Lymphocytes
Neutrophils
scr pept
corisin
WT/SAL
WT/BLM
c
BALF MUC-1 (ng/ml)
BALF SAP (ng/ml)
BALF periostin (ng/ml)
BALF osteopontin (ng/ml)
BALF CCL2 (pg/ml)
BALF collagen I (ng/ml)
d
Cleaved casp-3
βActin
20 kDa
47 kDa
e
Caspase-3/βactin ratio

Supplementary Fig. 22. Acute exacerbation of pulmonary fibrosis in mice with bleomycin-induced pulmonary fibrosis after intratracheal instillation of corisin. Wild-type (WT) mice received bleomycin (BLM) or saline (SAL) by osmotic minipumps on day 0 and then intratracheal instillation (75 µl) of corisin or scrambled peptide (300 µg/mouse) on day 20 after pump implantation. Bronchoalveolar lavage fluid (BALF) was collected as described under Methods. **a**, **b** The total cell count in BALF was determined using a nucleocounter, and the differential cell count was performed after Giemsa staining using the WinRoof image software. The number of mice: n=3 in WT/SAL/scr pept and WT/SAL/corisin groups, n=5 in WT/BLM/scr pept, and n=6 in WT/BLM/corisin group. The figure shows the representative microphotograph of stained BALF cells from each treatment group. Scale bars indicate 20 µm. Data are the mean ± S.D. Statistical analysis by ANOVA with a post hoc Newman-Keuls test. $*p<0.05$; $**p<0.01$; $****p<0.0001$. ns, not significant. scr pept, scrambled peptide. **c** The BALF levels of serum amyloid P component (SAP), MUC-1, chemokine (C-C motif) ligand 2 (CCL2), periostin, collagen I, and osteopontin were measured by enzyme immunoassays following the manufacturer's instructions. The number of mice: n=3 in WT/SAL/scr pept and WT/SAL/corisin groups, n=5 in WT/BLM/scr pept, and n=6 in WT/BLM/corisin group. Data are the mean ± S.D. Statistical analysis by ANOVA with a post hoc Newman-Keuls test. $*p<0.05$; $**p<0.01$; $***p<0.001$; $****p<0.0001$. ns, not significant. **d**, **e** Cleavage of caspase-3 was evaluated by Western blotting and quantified. N=3 in each group. Representative blots of two experiments are shown. Bars indicate the mean ± S.D. Statistical analysis by ANOVA with a post hoc Newman-Keuls test. $*p<0.05$; $**p<0.01$; $***p<0.001$. The source data underlying **b**, **c**, and **e** are provided in the Source Data file

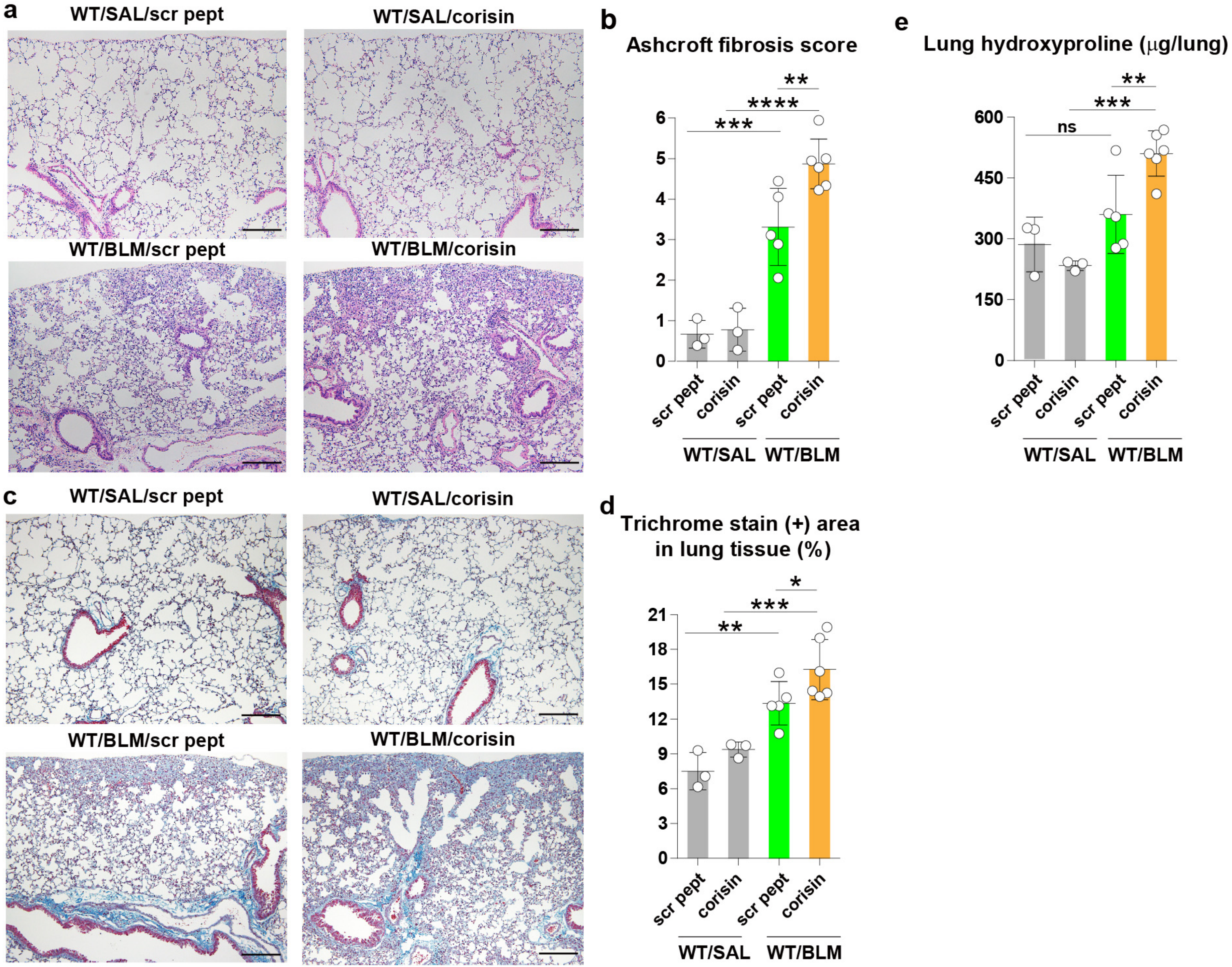
a
WT/SAL/scr pept
WT/SAL/corisin
WT/BLM/scr pept
WT/BLM/corisin
b
Ashcroft fibrosis score
0
1
2
3
4
5
6
***
****
**
scr pept
corisin
scr pept
corisin
WT/SAL
WT/BLM
c
WT/SAL/scr pept
WT/SAL/corisin
WT/BLM/scr pept
WT/BLM/corisin
d
Trichrome stain (+) area
in lung tissue (%)
0
3
6
9
12
15
18
21
**
***
*
scr pept
corisin
scr pept
corisin
WT/SAL
WT/BLM
e
Lung hydroxyproline (μg/lung)
0
150
300
450
600
ns
***
**
scr pept
corisin
scr pept
corisin
WT/SAL
WT/BLM

Supplementary Fig. 23. Acute exacerbation of pulmonary fibrosis in mice with bleomycin-induced pulmonary fibrosis after intratracheal instillation of corisin. Wild-type (WT) mice received bleomycin (BLM) or saline (SAL) by osmotic minipumps on day 0 and then intratracheal instillation (75 µl) of corisin or scrambled peptide (scr pept) (300 µg/mouse) on day 20 after pump implantation. Bronchoalveolar lavage fluid (BALF) was collected as described under Methods. **a**, **b** The grade of lung fibrosis was also evaluated by the Ashcroft fibrosis score as described under Methods. The number of mice: n=3 in WT/SAL/scr pept and WT/SAL/corisin groups, n=5 in WT/BLM/scr pept, and n=6 in WT/BLM/corisin group. Representative lung microphotograph of each group is shown. Scale bars indicate 200 μm. Data are the mean ± S.D. Statistical analysis by ANOVA with a post hoc Newman-Keuls test. $^{**}p<0.01$; $^{***}p<0.001$; $^{****}p<0.0001$. **c**, **d** Lung collagen deposition was evaluated by Masson's trichrome staining, and the percentage of trichrome stain (+) area was measured using the WinRoof Image Processing Software. The number of mice: n=3 in WT/SAL/scr pept and WT/SAL/corisin groups, n=5 in WT/BLM/scr pept, and n=6 in WT/BLM/corisin group. Representative lung microphotograph of each group is shown. Scale bars indicate 200 μm. Data are the mean ± S.D. Statistical analysis by ANOVA with a post hoc Newman-Keuls test. $^{*}p<0.05$; $^{**}p<0.01$; $^{***}p<0.001$. **e**, The lung tissue content of hydroxyproline, a marker of collagen deposition, was measured by a colorimetric assay using a commercially available kit following the manufacturer's instructions. The number of mice: n=3 in WT/SAL/scr pept and WT/SAL/corisin groups, n=5 in WT/BLM/scr pept, and n=6 in WT/BLM/corisin group. Bars indicate the mean ± S.D. Statistical analysis by ANOVA with a post hoc Newman-Keuls test. $^{**}p<0.01$; $^{***}p<0.001$. ns, not significant. The source data underlying **b**, **d**, and **e** are provided in the Source Data file.

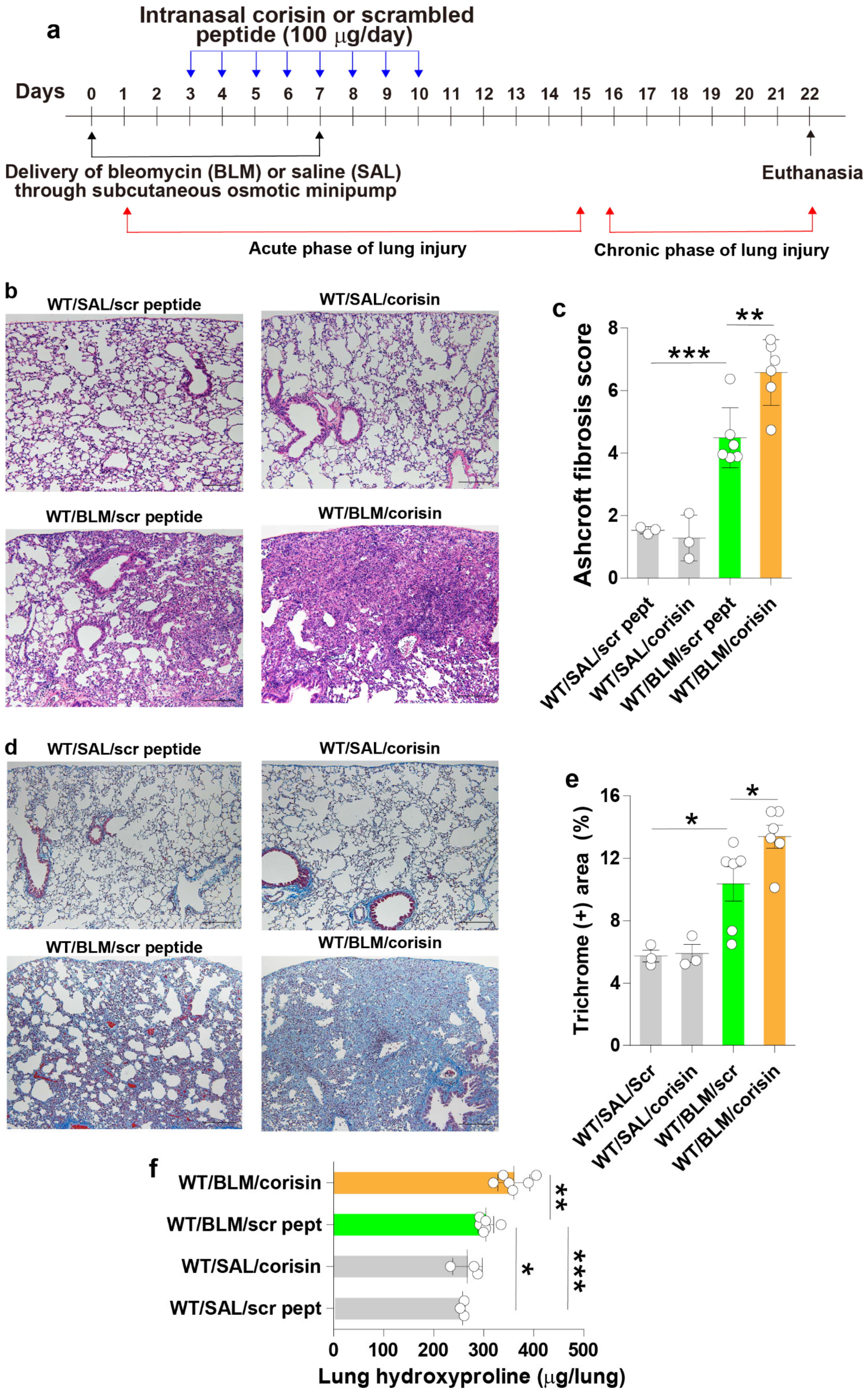

a
Intranasal corisin or scrambled peptide (100 μg/day)
Days 0 1 2 3 4 5 6 7 8 9 10 11 12 13 14 15 16 17 18 19 20 21 22
Delivery of bleomycin (BLM) or saline (SAL) through subcutaneous osmotic minipump
Euthanasia
Acute phase of lung injury
Chronic phase of lung injury
b
WT/SAL/scr peptide
WT/SAL/corisin
WT/BLM/scr peptide
WT/BLM/corisin
c
Ashcroft fibrosis score
0
2
4
6
8
***
**
WT/SAL/scr pept
WT/SAL/corisin
WT/BLM/scr pept
WT/BLM/corisin
d
WT/SAL/scr peptide
WT/SAL/corisin
WT/BLM/scr peptide
WT/BLM/corisin
e
Trichrome (+) area (%)
0
4
8
12
16
*
*
WT/SAL/Scr
WT/SAL/corisin
WT/BLM/scr
WT/BLM/corisin
f
WT/BLM/corisin
WT/BLM/scr pept
WT/SAL/corisin
WT/SAL/scr pept
**
*
***
0 100 200 300 400 500
Lung hydroxyproline (μg/lung)

Supplementary Fig. 24. Mice receiving intranasal corisin at early stages of bleomycin-induced lung injury develop advanced pulmonary fibrosis. **a**, Mice received bleomycin (BLM) infusion through osmotic mini-pump for 7 days to induce lung injury/fibrosis, and treated with intranasal corisin (100 µg) or scrambled peptide (100 µg) on days 3, 4, 5, 7, 9, 10, and 11 and euthanized on day 22 after starting BLM infusion. **b**, **c** Lung tissue samples were stained with hematoxylin & eosin, and Ashcroft fibrosis score was performed as described under Methods. The number of mice: n=3 in WT/SAL/scr pept and WT/SAL/corisin groups, n=6 in WT/BLM/scr pept, and WT/BLM/corisin groups. Representative lung microphotograph of each group is shown. Scale bars indicate 200 µm. Data are the mean ± S.D. Statistical analysis by ANOVA with a post hoc Newman-Keuls test. **p<0.01; ***p<0.001. **d**, **e** Lung tissue was stained with trichrome and the area with collagen deposition was evaluated using the WinRoof Image Processing Software. The number of mice: n=3 in WT/SAL/scr pept and WT/SAL/corisin groups, n=6 in WT/BLM/scr pept, and WT/BLM/corisin groups. Representative lung microphotograph of each group is shown. Scale bars indicate 200 µm. Data are the mean ± S.D. Statistical analysis by ANOVA with a post hoc Newman-Keuls test. *p<0.05. **f** The lung tissue content of hydroxyproline was measured by a colorimetric assay using a commercially available kit following the manufacturer's instructions. The number of mice: n=3 in WT/SAL/scr pept and WT/SAL/corisin groups, n=6 in WT/BLM/scr pept, and WT/BLM/corisin groups. Bars indicate the mean ± S.D. Statistical analysis by ANOVA with a post hoc Newman-Keuls test. *p<0.05; **p<0.01; ***p<0.001. The source data underlying **c**, **e**, and **f** are provided in the Source Data file.

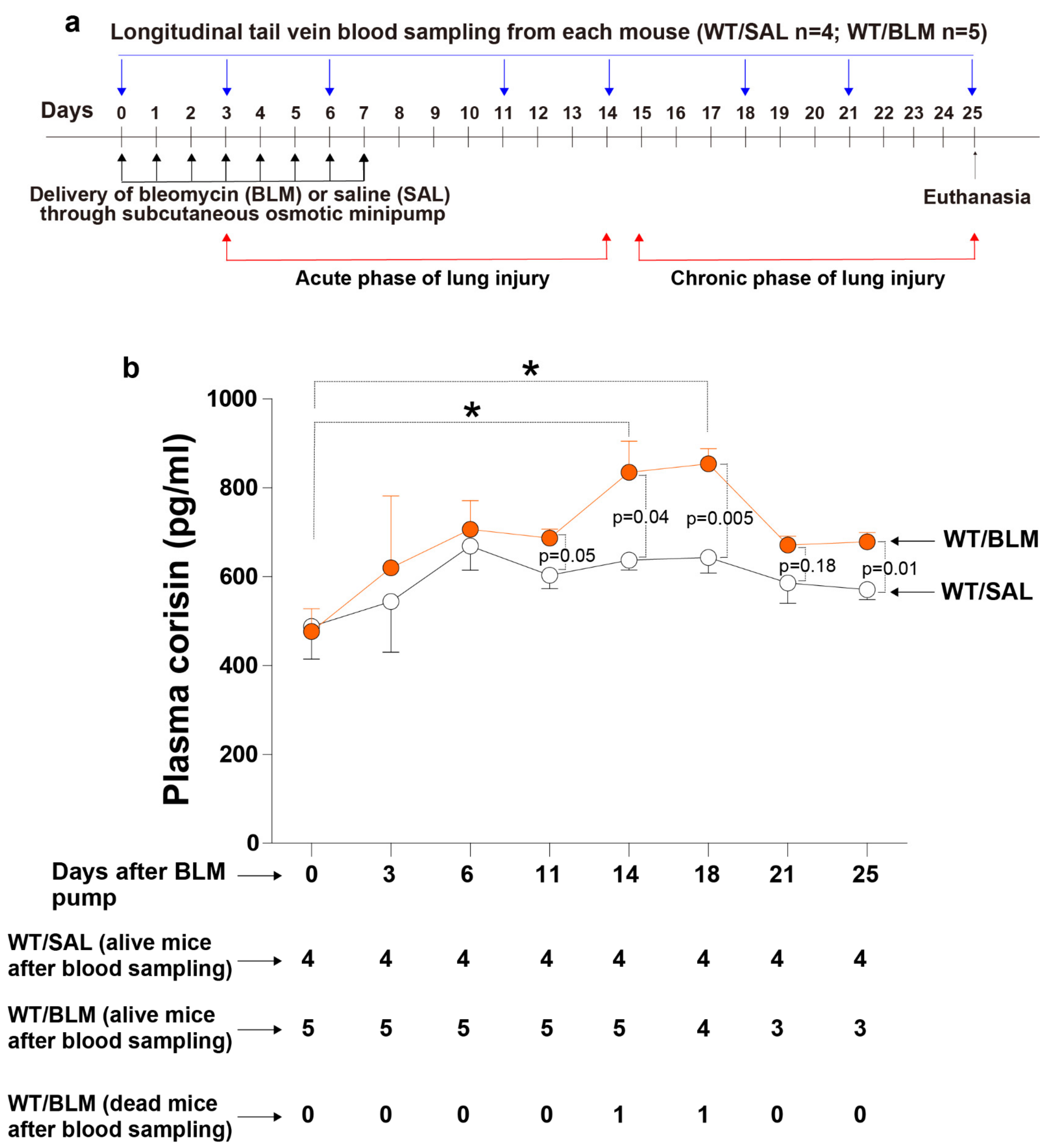

Supplementary Fig. 25. Longitudinal changes of circulating native corisin during bleomycin-induced pulmonary fibrosis. a, **b** Mice were infused BLM (n=5) or saline (n=4) through an osmotic mini-pump for 7 days, and then blood samples were longitudinally collected from the tail vein of each mouse on days 0, 3, 6, 11, 14, 18, 21, and 25. Corisin was measured in plasma by an immunoassay as described under Methods. Data are the mean ± S.E.M. Statistical difference in the concentration of corisin between days was evaluated by ANOVA with a post hoc Newman-Keuls test, and statistical difference between WT/SAL and WT/BLM groups was evaluated by two-sided unpaired t-test. *$p<0.05$. The source data underlying **b** are provided in the Source Data file.

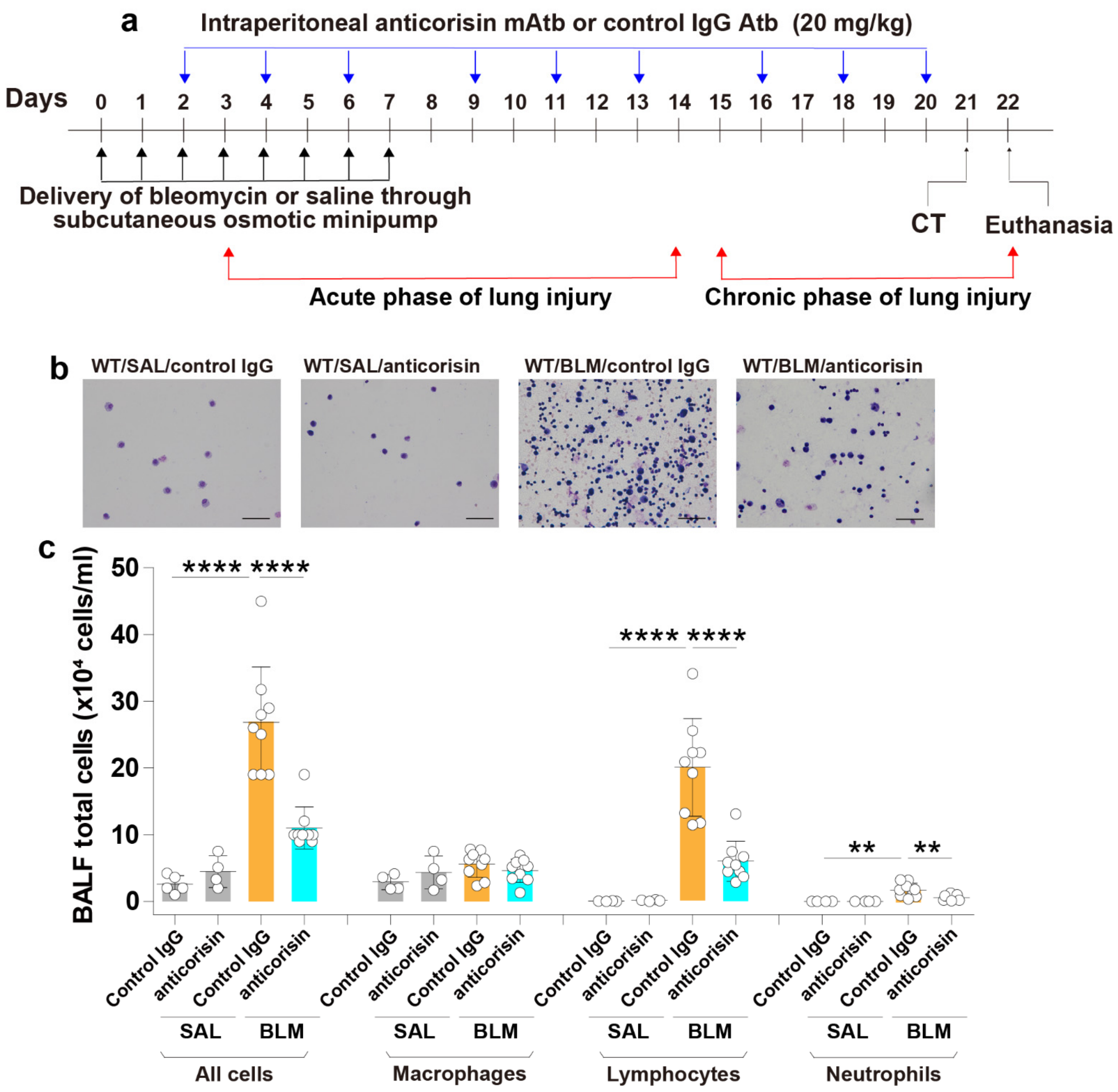

Supplementary Fig. 26. Reduced lung inflammatory cell infiltration in mice with bleomycin-induced pulmonary fibrosis treated with anticorisin monoclonal antibody. a Wild-type (WT) mice received bleomycin (BLM) by osmotic minipumps and were treated with anticorisin monoclonal antibody (mAtb) (WT/BLM/anticorisin) or control IgG (WT/BLM/control IgG) by intraperitoneal route three times a week for three weeks. WT mice receiving saline (SAL) by osmotic minipumps and treated with anticorisin mAtb (WT/SAL/anticorisin) or control IgG (WT/SAL/control IgG) by intraperitoneal route three times a week for three weeks were the control mice. Mice were sacrificed on day 22. **b**, **c** The total cell count in BALF was determined using a nucleocounter, and the differential cell count was performed after Giemsa staining using the WinRoof image software. The figure shows the representative microphotograph of stained BALF cells from each treatment group. The number of mice: n=4 in WT/SAL/control IgG and WT/SAL/anticorisin groups, n=9 in WT/BLM/anticorisin and WT/BLM/control IgG groups. Scale bars indicate 50 µm. Data are the mean ± S.D. Statistical analysis by ANOVA with a post hoc Newman-Keuls test. **p<0.01, ****p<0.0001. The source data underlying **c** are provided in the Source Data file.

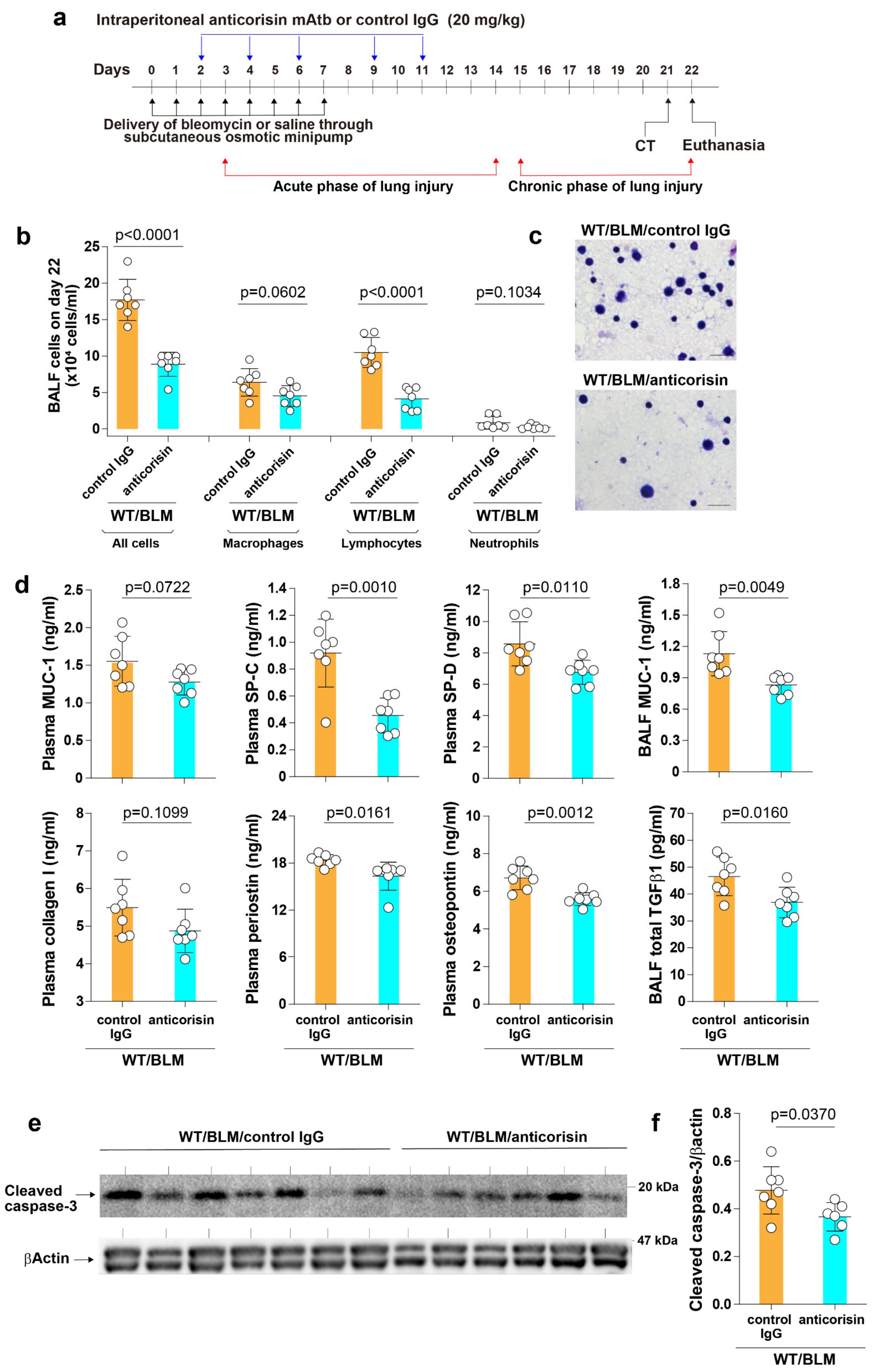

a
Intraperitoneal anticorisin mAtb or control IgG (20 mg/kg)
Days 0 1 2 3 4 5 6 7 8 9 10 11 12 13 14 15 16 17 18 19 20 21 22
Delivery of bleomycin or saline through subcutaneous osmotic minipump
CT
Euthanasia
Acute phase of lung injury
Chronic phase of lung injury
b
BALF cells on day 22 (x10⁴ cells/ml)
p<0.0001
p=0.0602
p<0.0001
p=0.1034
control IgG
anticorisin
WT/BLM
All cells
Macrophages
Lymphocytes
Neutrophils
c
WT/BLM/control IgG
WT/BLM/anticorisin
d
Plasma MUC-1 (ng/ml)
p=0.0722
Plasma SP-C (ng/ml)
p=0.0010
Plasma SP-D (ng/ml)
p=0.0110
BALF MUC-1 (ng/ml)
p=0.0049
Plasma collagen I (ng/ml)
p=0.1099
Plasma periostin (ng/ml)
p=0.0161
Plasma osteopontin (ng/ml)
p=0.0012
BALF total TGFβ1 (pg/ml)
p=0.0160
control IgG
anticorisin
WT/BLM
e
WT/BLM/control IgG
WT/BLM/anticorisin
Cleaved caspase-3
βActin
20 kDa
47 kDa
f
Cleaved caspase-3/βactin
p=0.0370
control IgG
anticorisin
WT/BLM

Supplementary Fig. 27. Reduced markers of acute lung injury and apoptosis in lung tissue in mice with bleomycin-induced pulmonary fibrosis treated with anticorisin monoclonal antibody during the acute phase of the disease. a Wild-type (WT) mice received bleomycin (BLM) by osmotic minipumps and treated with anticorisin monoclonal antibody (WT/BLM/anticorisin) or control IgG (WT/BLM/control IgG) by an intraperitoneal route on days 2, 4, 6, 9 and 11 after BLM pump implantation. N=7 in both treatment groups. **b**, **c** Bronchoalveolar lavage fluid (BALF) was collected on day 22 after the BLM pump as described under Methods. The total cell count in BALF was determined using a nucleocounter, and the differential cell count was performed after Giemsa staining using the WinRoof image software. The figure shows the representative microphotograph of stained BALF cells from each treatment group. N=7 in both treatment groups. Scale bars indicate 20 μm. Data are the mean ± S.D. Statistical analysis by two-sided unpaired t-test. **d**, The levels of MUC-1, surfactant protein C (SP-C), SP-D, collagen I, periostin, osteopontin, and total TGFβ1 were measured by commercial enzyme immunoassay kits following the manufacturer's instruction. Data are the mean ± S.D. Statistical analysis was performed by a two-sided unpaired t-test. **e**, **f** Western blotting of cleaved caspase-3 and βactin. N=7 in WT/BLM/control IgG group and n=6 in the WT/BLM/anticorisin group. Representative blots from mice of each treatment group are shown. Data are the mean ± S.D. Statistical analysis was performed by a two-sided unpaired t-test. The source data underlying **b**, **d,** and **f** are provided in the Source Data file.

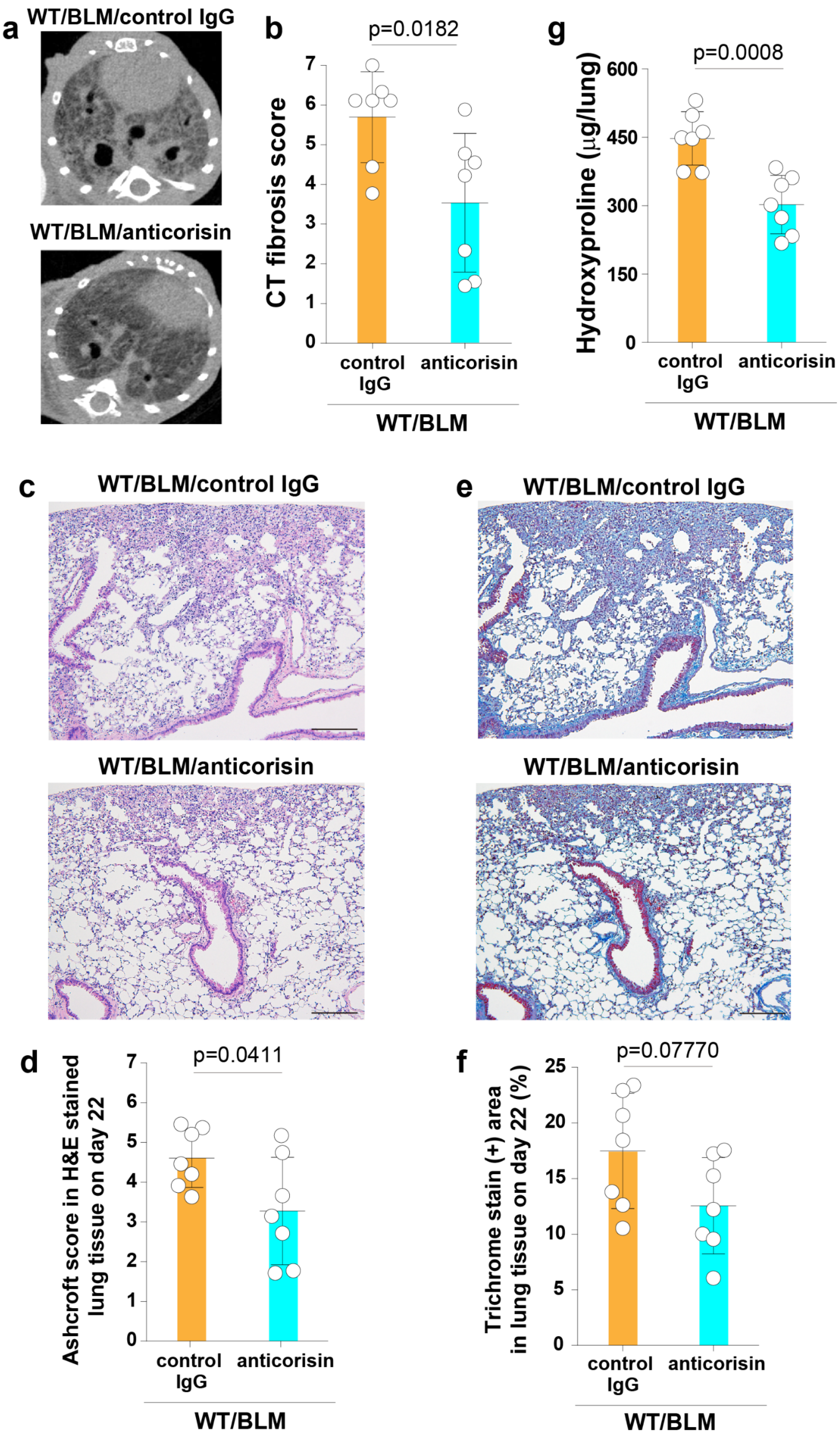
a
WT/BLM/control IgG
WT/BLM/anticorisin
b
p=0.0182
CT fibrosis score
0
1
2
3
4
5
6
7
control IgG
anticorisin
WT/BLM
g
p=0.0008
Hydroxyproline (μg/lung)
0
150
300
450
600
control IgG
anticorisin
WT/BLM
c
WT/BLM/control IgG
WT/BLM/anticorisin
e
WT/BLM/control IgG
WT/BLM/anticorisin
d
p=0.0411
Ashcroft score in H&E stained lung tissue on day 22
0
1
2
3
4
5
6
7
control IgG
anticorisin
WT/BLM
f
p=0.07770
Trichrome stain (+) area in lung tissue on day 22 (%)
0
5
10
15
20
25
control IgG
anticorisin
WT/BLM

Supplementary Fig. 28. Amelioration of lung fibrosis in mice with bleomycin-induced pulmonary fibrosis treated with anticorisin monoclonal antibody during the acute phase of the disease. Wild-type (WT) mice received bleomycin (BLM) by osmotic minipumps and were treated with anticorisin monoclonal antibody (WT/BLM/anticorisin) or control IgG (WT/BLM/control IgG) by an intraperitoneal route on days 2, 4, 6, 9 and 11 after BLM pump implantation. N=7 in both treatment groups. **a**, **b** Computed tomography (CT) was performed on day 21 (chronic phase) after the BLM pump implantation. The radiological findings of lung fibrosis were evaluated using a CT fibrosis score as described under Methods. Representative CT findings in each group are shown. Data are the mean ± S.D. Statistical analysis was performed by a two-sided unpaired t-test. **c**, **d** Experts blinded for the treatment groups scored the grade of lung fibrosis in hematoxylin & eosin (H&E)-stained lung tissue using the Ashcroft's score. Representative lung microphotograph of each group is shown. Scale bars indicate 200 μm. N=7 in both WT/BLM/control IgG and WT/BLM/anticorisin groups. Bars indicate the mean ± S.D. Statistical analysis was performed by a two-sided unpaired t-test. **e**, **f** Lung collagen deposition was evaluated by Masson's trichrome staining, and the percentage of trichrome stain (+) area was measured using the WinRoof Image Processing Software. Representative lung microphotograph of each group is shown. Scale bars indicate 200 μm. N=7 in both WT/BLM/control IgG and WT/BLM/anticorisin groups. Bars indicate the mean ± S.D. Statistical analysis was performed by a two-sided unpaired t-test. **g** The hydroxyproline content in the lungs was measured by a commercially available colorimetric assay following the manufacturer's instructions. N=7 in both WT/BLM/control IgG and WT/BLML/anticorisin groups. Bars indicate the mean ± S.D. Statistical analysis was performed by a two-sided unpaired t-test. The source data underlying **b**, **d**, **f**, and **g** are provided in the Source Data file.

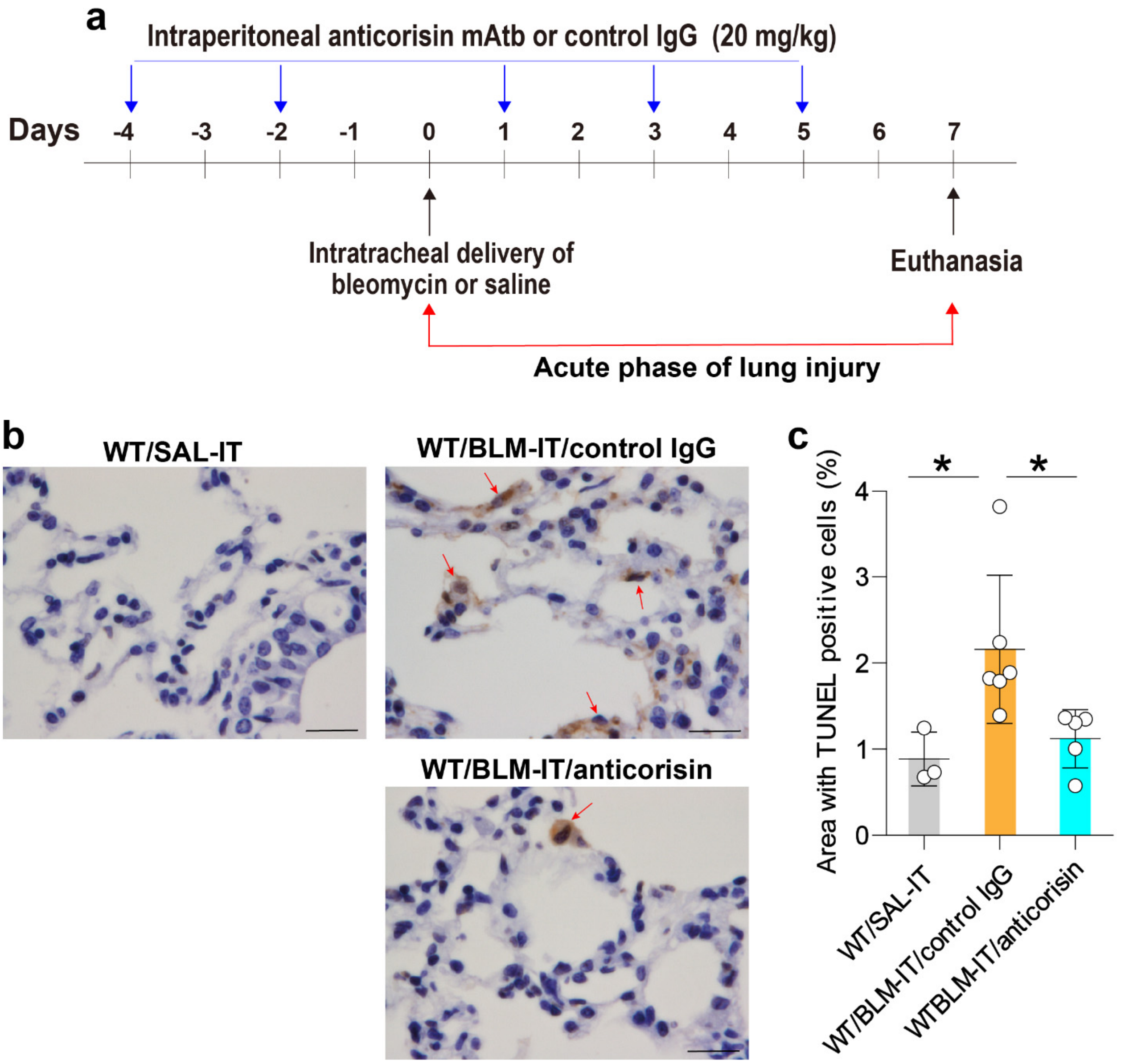

Supplementary Fig. 29. Anticorisin mAtb strongly inhibits apoptosis during the acute phase of BLM-induced lung injury. a Two groups of wild-type (WT) mice received bleomycin (BLM) by intratracheal delivery and were treated with anticorisin monoclonal antibody or control IgG. N=6 in WT/BLM/control IgG, n=5 in WT/BLM/anticorisin mAtb. Untreated WT mice receiving saline (SAL) by intratracheal delivery were the controls (n=3). **b**, **c** DNA fragmentation was evaluated by staining with terminal deoxynucleotidyltransferase dUTP Nick-End Labeling (TUNEL) and then quantified using the image WinRoof software. Representative lung microphotograph of each group is shown. Scale bars indicate 20 µm. Arrows indicate apoptotic cells. Bars indicate the means ± S.D. Statistical analysis by ANOVA with a post hoc Newman-Keuls test. *p<0.05. The source data underlying **c** are provided in the Source Data file.

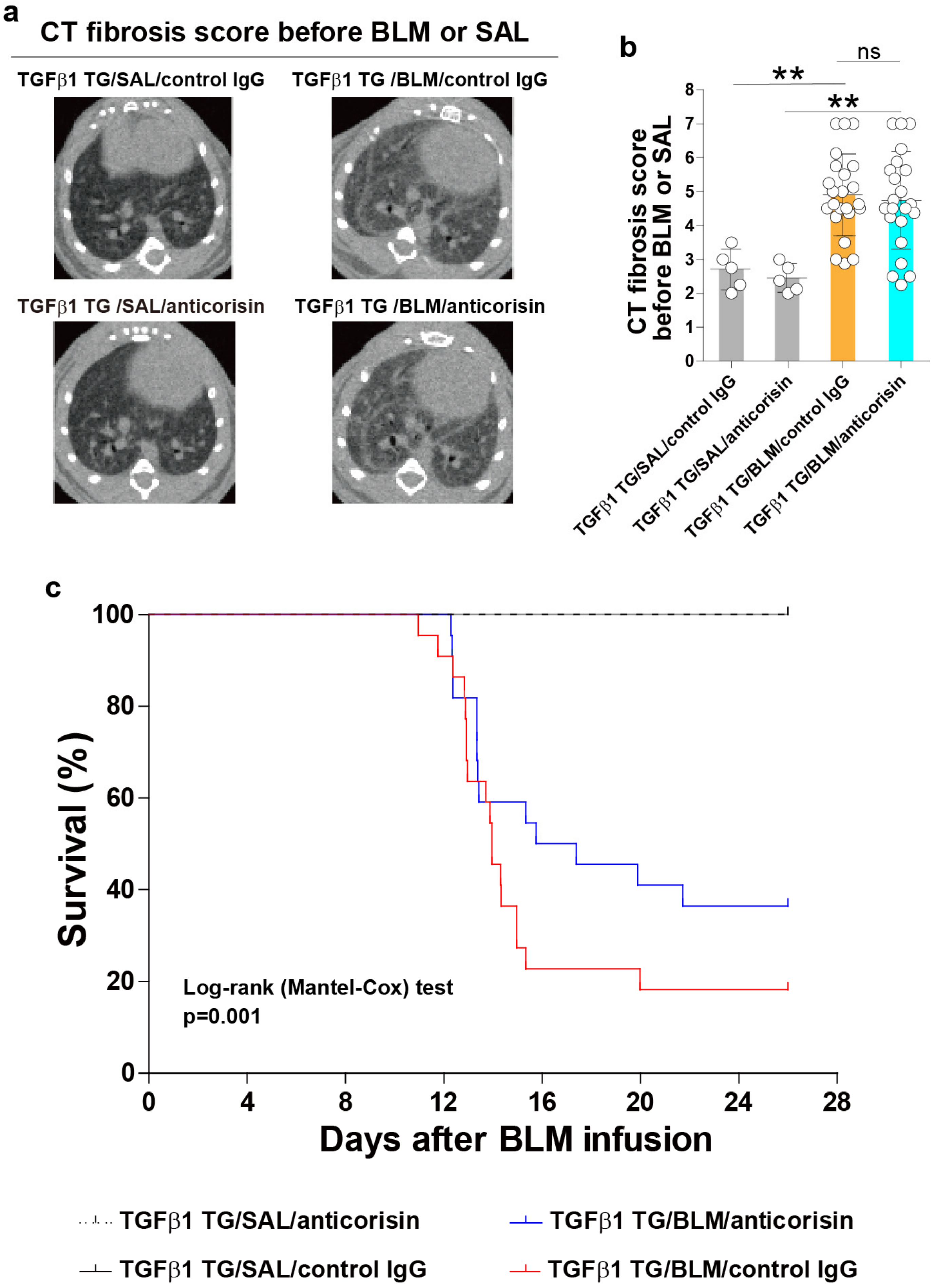
a
CT fibrosis score before BLM or SAL
TGFβ1 TG/SAL/control IgG
TGFβ1 TG /BLM/control IgG
TGFβ1 TG /SAL/anticorisin
TGFβ1 TG /BLM/anticorisin
b
ns
**
**
CT fibrosis score before BLM or SAL
0
1
2
3
4
5
6
7
8
TGFβ1 TG/SAL/control IgG
TGFβ1 TG/SAL/anticorisin
TGFβ1 TG/BLM/control IgG
TGFβ1 TG/BLM/anticorisin
c
Survival (%)
100
80
60
40
20
0
Log-rank (Mantel-Cox) test
p=0.001
0
4
8
12
16
20
24
28
Days after BLM infusion
TGFβ1 TG/SAL/anticorisin
TGFβ1 TG/BLM/anticorisin
TGFβ1 TG/SAL/control IgG
TGFβ1 TG/BLM/control IgG

Supplementary Fig. 30. Anticorisin antibody prolongs the survival of mice with acute exacerbation of pulmonary fibrosis. a, **b** Computed tomography (CT) was performed in human transforming growth factorβ1 (TGFβ1) transgenic (TG) mice. The radiological findings of lung fibrosis were evaluated using a CT fibrosis score as described under Methods. TGFβ1 TG mice with lung fibrosis were separated into two groups with matched CT fibrosis scores to receive bleomycin (BLM) through osmotic minipumps. One of these two groups received intraperitoneal injection of anticorisin mAtb (TGFβ1 TG/BLM/anticorisin), whereas the other group received intraperitoneal injection of control IgG (TGFβ1 TG/BLM/control IgG). TGFβ1 TG mice without lung fibrosis receiving saline infusion through osmotic minipumps and treated with anticorisin mAtb (TGFβ1 TG/SAL/anticorisin) or control IgG (TGFβ1 TG/SAL/control IgG) were the mouse controls. Representative CT findings of each group are shown. N=22 in both TGFβ1 TG/BLM/control IgG and TGFβ1 TG/BLM/anticorisin groups, and n=5 in TGFβ1 TG/SAL/control IgG and TGFβ1 TG/SAL/anticorisin groups. Representative CT findings in each group are shown. Bars indicate the mean ± S.D. Statistical analysis was performed by ANOVA with a post hoc Newman-Keuls test. **p<0.01. ns, not significant. **c** The survival was monitored. Statistical analysis was performed by the log-rank (Mantel-Cox) test. p=0.001. The source data underlying **b**, and **c** are provided in the Source Data file.

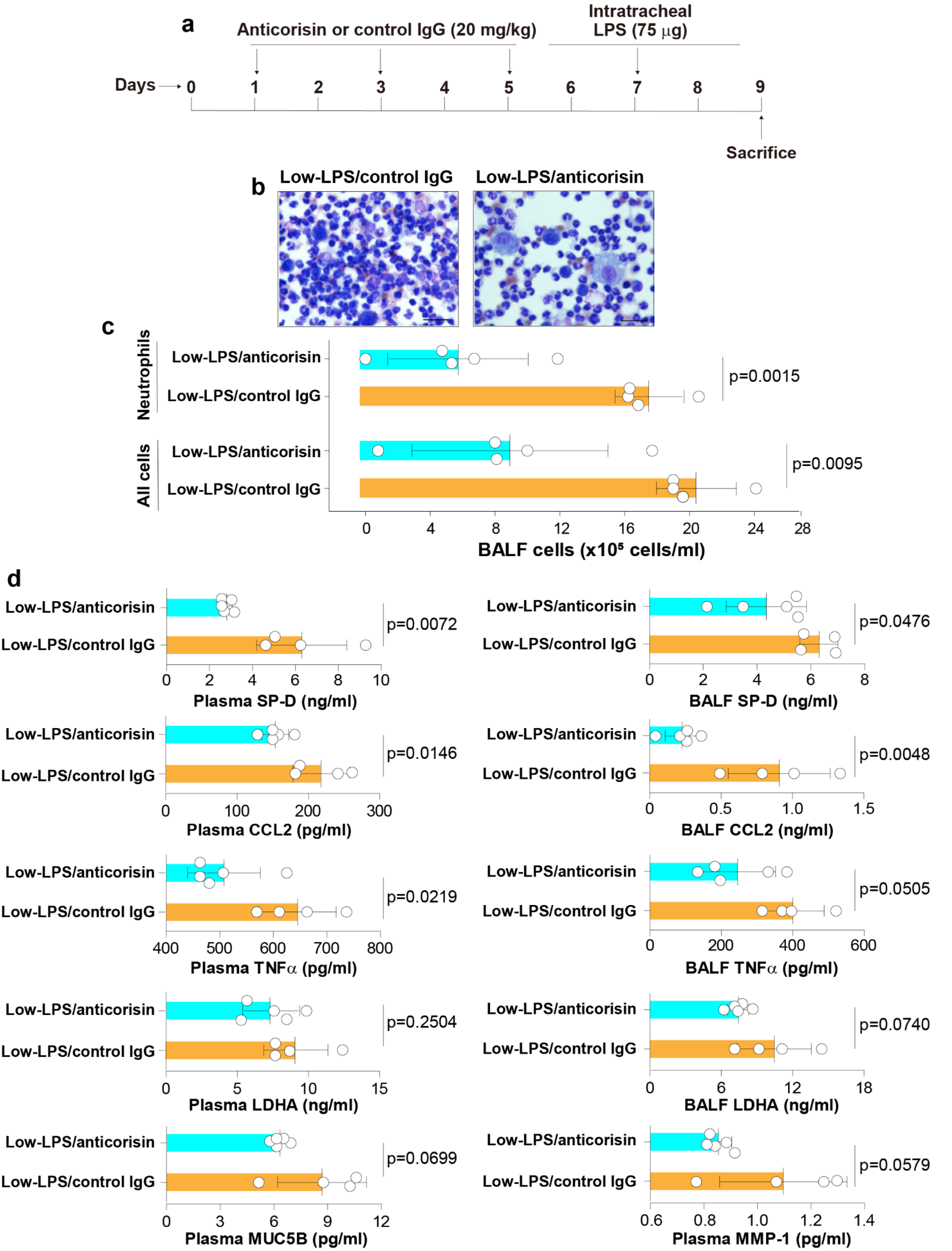

a
Anticorisin or control IgG (20 mg/kg)
Intratracheal LPS (75 μg)
Days
0 1 2 3 4 5 6 7 8 9
Sacrifice
b
Low-LPS/control IgG
Low-LPS/anticorisin
c
Neutrophils
All cells
Low-LPS/anticorisin
Low-LPS/control IgG
p=0.0015
p=0.0095
0 4 8 12 16 20 24 28
BALF cells (x10⁵ cells/ml)
d
Low-LPS/anticorisin
Low-LPS/control IgG
p=0.0072
Plasma SP-D (ng/ml)
p=0.0476
BALF SP-D (ng/ml)
p=0.0146
Plasma CCL2 (pg/ml)
p=0.0048
BALF CCL2 (ng/ml)
p=0.0219
Plasma TNFα (pg/ml)
p=0.0505
BALF TNFα (pg/ml)
p=0.2504
Plasma LDHA (ng/ml)
p=0.0740
BALF LDHA (ng/ml)
p=0.0699
Plasma MUC5B (pg/ml)
p=0.0579
Plasma MMP-1 (pg/ml)

Supplementary Fig. 31. Monoclonal anticorisin mAtb attenuates moderate lipopolysaccharide-induced acute lung injury. a Wild-type mice were treated three times with control IgG or anticorisin monoclonal antibody at a dose of 20 mg/kg by intraperitoneal route once a day every other day. Mice received intratracheal instillation of a low-dose (75 μg) of lipopolysaccharide (LPS) two days after the last treatment with antibody and sacrificed two days after LPS instillation. **b**, **c** Mice were sacrificed on day 2 after intratracheal LPS instillation, and bronchoalveolar lavage fluid (BALF) was collected. BALF cells were counted using a nucleocounter and stained with Giemsa for differential cell counting as described under Methods. Representative microphotograph of stained BALF cells in each group is shown. Scale bars indicate 20 μm. N=4 in LPS/control IgG and n=5 in LPS/anticorisin group. Data are the mean ± S.D. Statistical analysis by two-sided unpaired t-test. **p<0.01. **d** The levels of surfactant protein D (SP-D), chemokine (C-C motif) ligand 2 (CCL2), TNFα, lactate dehydrogenase A (LDHA), MUC-5B, and matrix metalloproteinase-1 (MMP-1) were measured using a commercially available immunoassay kit following the manufacturers' instruction. N=4 in LPS/control IgG and n=5 in LPS/anticorisin groups. Data are the mean ± S.D. Statistical analysis by two-sided unpaired t-test. *p<0.05, **p<0.01. ns, not significant. The source data underlying **c**, and **d** are provided in the Source Data file.

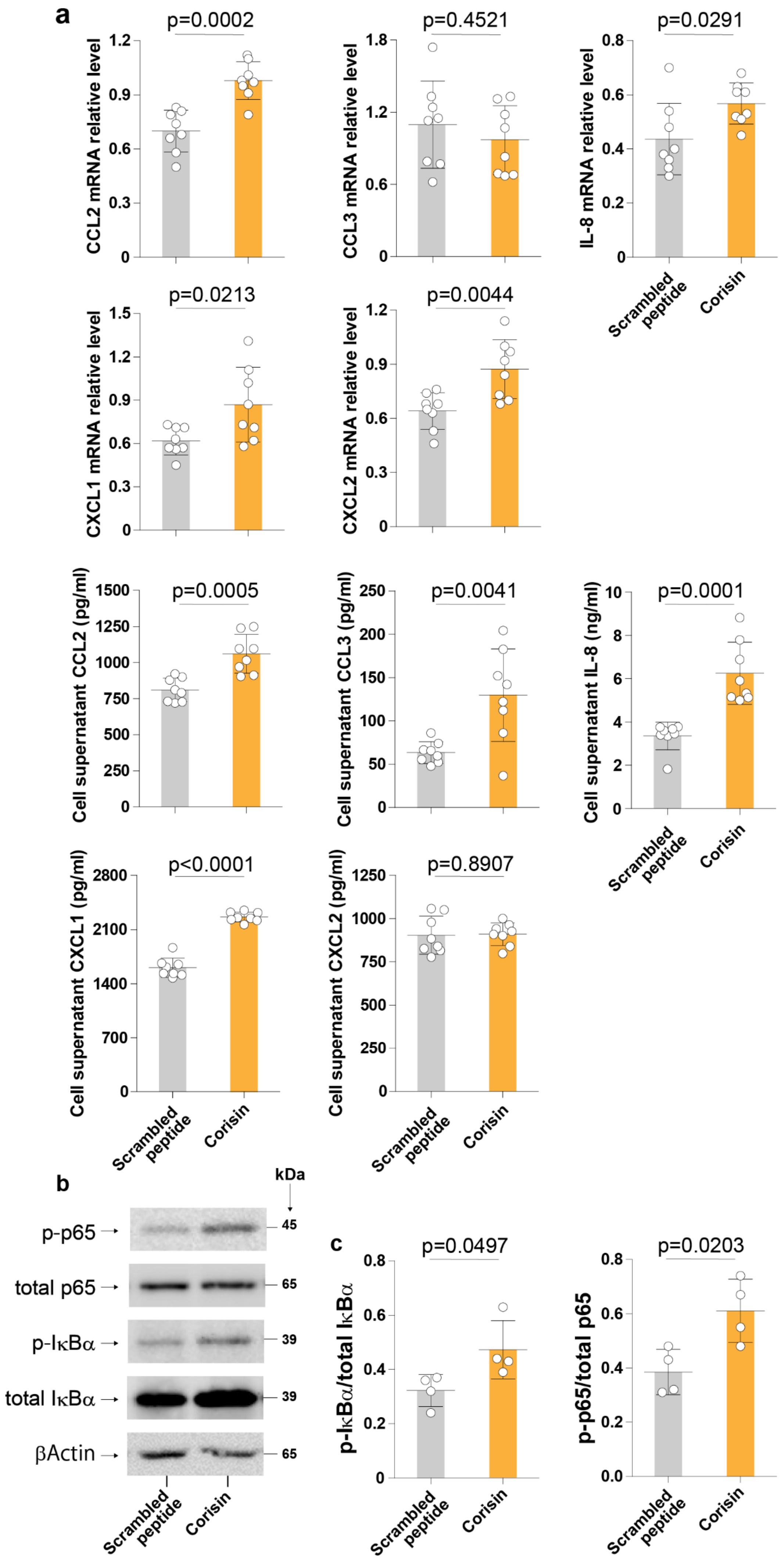

a
p=0.0002
CCL2 mRNA relative level
p=0.4521
CCL3 mRNA relative level
p=0.0291
IL-8 mRNA relative level
p=0.0213
CXCL1 mRNA relative level
p=0.0044
CXCL2 mRNA relative level
p=0.0005
Cell supernatant CCL2 (pg/ml)
p=0.0041
Cell supernatant CCL3 (pg/ml)
p=0.0001
Cell supernatant IL-8 (ng/ml)
p<0.0001
Cell supernatant CXCL1 (pg/ml)
p=0.8907
Cell supernatant CXCL2 (pg/ml)
Scrambled peptide
Corisin
b
kDa
p-p65
total p65
p-IκBα
total IκBα
βActin
45
65
39
39
65
c
p=0.0497
p-IκBα/total IκBα
p=0.0203
p-p65/total p65

Supplementary Fig. 32. Corisin increases the expression of cytokines and chemokines in alveolar epithelial cells. a A549 alveolar epithelial cells were cultured in the presence of 10 μg/ml of corisin or scrambled peptide for 24h. The cell supernatants were collected and then the total mRNA was extracted, and cDNA was prepared before amplification by RT-PCR. Chemokines were measured using commercially available immunoassay kits following the instructions of the manufacturers. N=8 in each group. Data are expressed as the mean ± S.D. Statistical analysis was performed using the two-sided unpaired t-test. **b**, **c** In a separate experiment, A549 cells were cultured and treated as described above and cell lysates were prepared to perform Western blotting as described under Methods. A representative blot of each group from two experiments is shown. N=8 in each group. Data are expressed as the mean ± S.D. Statistical analysis was performed using the two-sided unpaired t-test. The source data underlying **a**, and **c** are provided in the Source Data file.

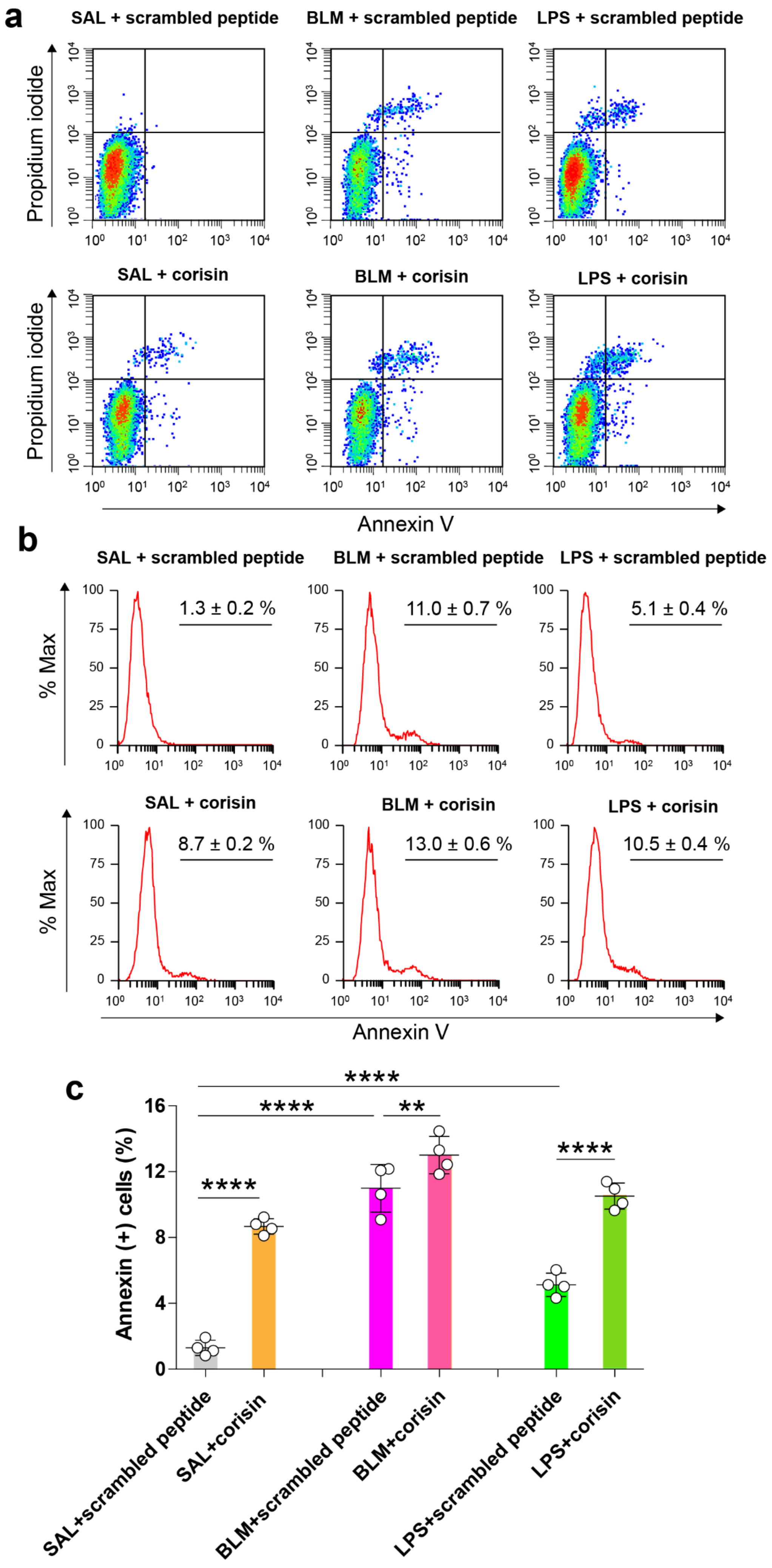

a
SAL + scrambled peptide
BLM + scrambled peptide
LPS + scrambled peptide
SAL + corisin
BLM + corisin
LPS + corisin
Propidium iodide
Annexin V
b
SAL + scrambled peptide
BLM + scrambled peptide
LPS + scrambled peptide
1.3 ± 0.2 %
11.0 ± 0.7 %
5.1 ± 0.4 %
SAL + corisin
BLM + corisin
LPS + corisin
8.7 ± 0.2 %
13.0 ± 0.6 %
10.5 ± 0.4 %
% Max
Annexin V
c
Annexin (+) cells (%)
SAL+scrambled peptide
SAL+corisin
BLM+scrambled peptide
BLM+corisin
LPS+scrambled peptide
LPS+corisin
****
**

Supplementary Fig. 33. Corisin increases the proapoptotic activities of bleomycin and lipopolysaccharide in alveolar epithelial cells. a, **b** A549 alveolar epithelial cells were serum-starved overnight and then cultured in the presence of 10 μg/ml of corisin alone or 10 μg/ml of corisin in combination with 50 μg/ml of bleomycin (BLM) or 10 μg/ml of lipopolysaccharide (LPS) to evaluate apoptosis after 24h. A549 cells treated with scrambled peptide were used as controls. **c** The percentage of apoptotic cells was determined by flow cytometry and quantified. N=4 in each group. Data are expressed as the mean ± S.D. Statistical analysis was performed by ANOVA with a post hoc Newman-Keuls test. $^{**}p<0.01$; $^{****}p<0.0001$. The source data underlying **c** are provided in the Source Data file.

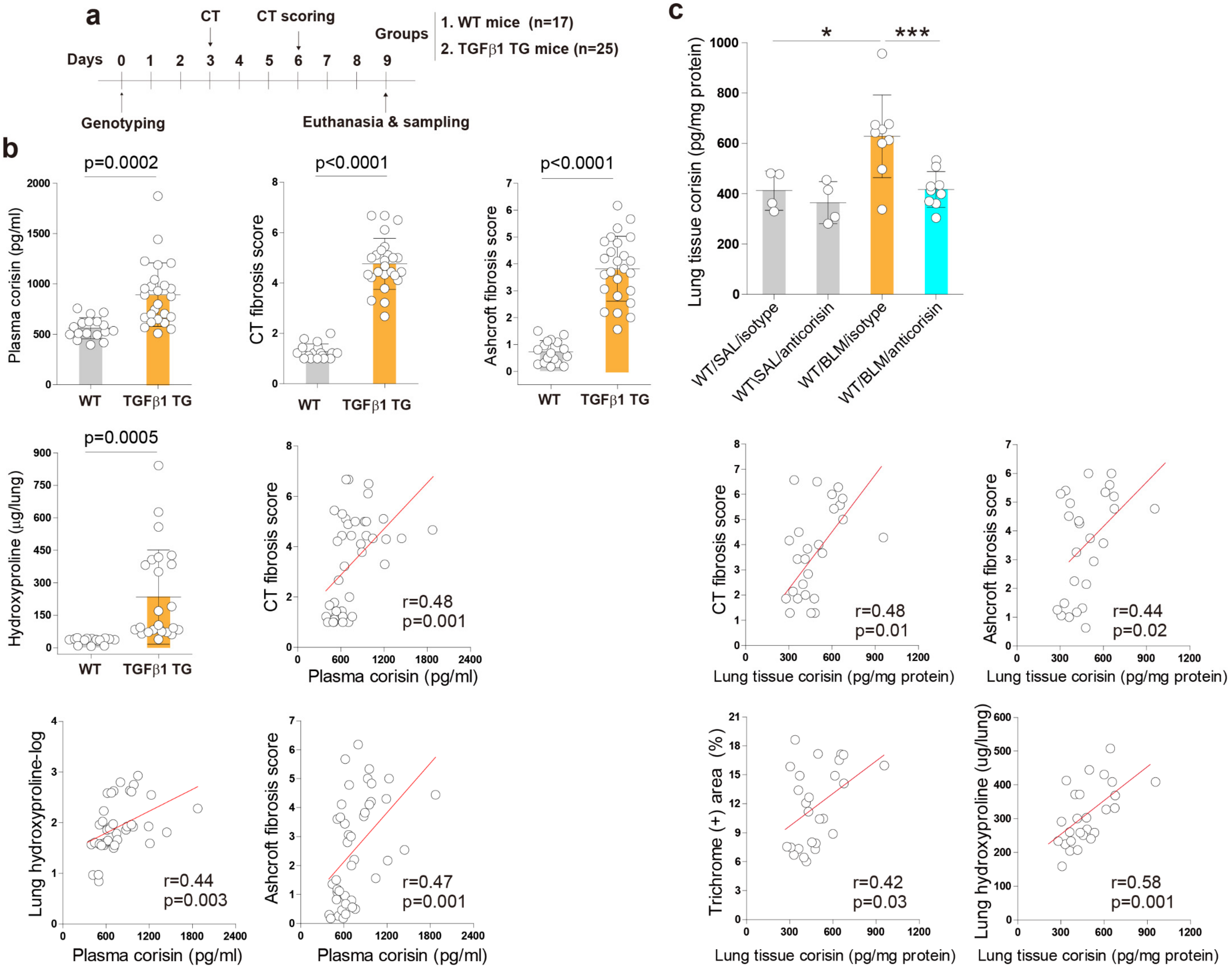
a
Days 0 1 2 3 4 5 6 7 8 9
CT
CT scoring
Genotyping
Euthanasia & sampling
Groups
1. WT mice (n=17)
2. TGFβ1 TG mice (n=25)
b
p=0.0002
Plasma corisin (pg/ml)
WT
TGFβ1 TG
p<0.0001
CT fibrosis score
p<0.0001
Ashcroft fibrosis score
p=0.0005
Hydroxyproline (μg/lung)
r=0.48
p=0.001
Plasma corisin (pg/ml)
Lung hydroxyproline-log
r=0.44
p=0.003
r=0.47
p=0.001
c
*
***
Lung tissue corisin (pg/mg protein)
WT/SAL/isotype
WT\SAL/anticorisin
WT/BLM/isotype
WT/BLM/anticorisin
r=0.48
p=0.01
r=0.44
p=0.02
Trichrome (+) area (%)
r=0.42
p=0.03
Lung hydroxyproline (ug/lung)
r=0.58
p=0.001

Supplementary Fig. 34. Increased circulating and lung tissue levels of corisin correlate with markers of pulmonary fibrosis. a Transforming growth factorβ1 (TGFβ1) transgenic (TG) mice were genotyped, computed tomography (CT) was performed and the findings of lung fibrosis were score as described under Methods. Mice were sacrificed for blood and lung tissue sampling. N=17 in the WT mice group and n=25 in the TGFβ1 TG mouse group. **b** The Ashcroft score of lung fibrosis and measurements of plasma corisin and lung hydroxyproline were performed as described under Methods. Bars indicate the mean ± S.D. Statistical difference between groups was analyzed by two-sided unpaired t-test and the strength of relationship by Pearson-product moment correlation. ***p<0.001 ****p<0.0001. **c** Samples of mouse groups from the experiment described in **Figure 6**. The Ashcroft score of lung fibrosis and measurements of plasma corisin, lung fibrosis area, and lung hydroxyproline were performed as described under Methods. Bars indicate the mean ± S.D. Statistical difference between groups was analyzed by ANOVA with a post hoc Newman-Keuls test, and the strength of relationship by Pearson-product moment correlation. *p<0.05 ***p<0.001. The source data underlying **b**, and **c** are provided in the Source Data file.

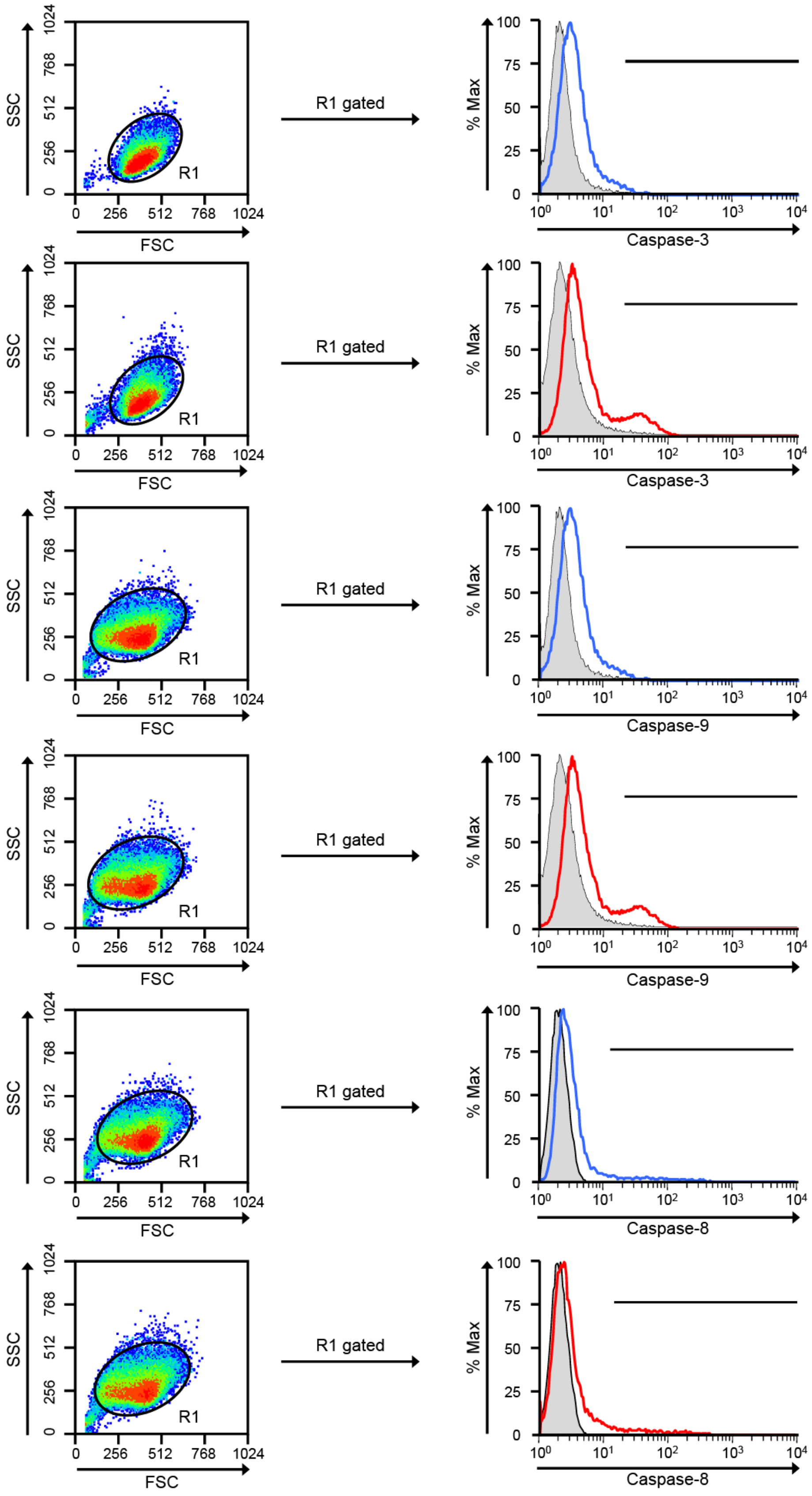

Supplementary Fig. 35. Flow cytometry gating strategy. Gating strategies used in the experiments described in Supplementary Figure 5a. Abbreviations: SSC, side scatter; FSC, forward scatter.

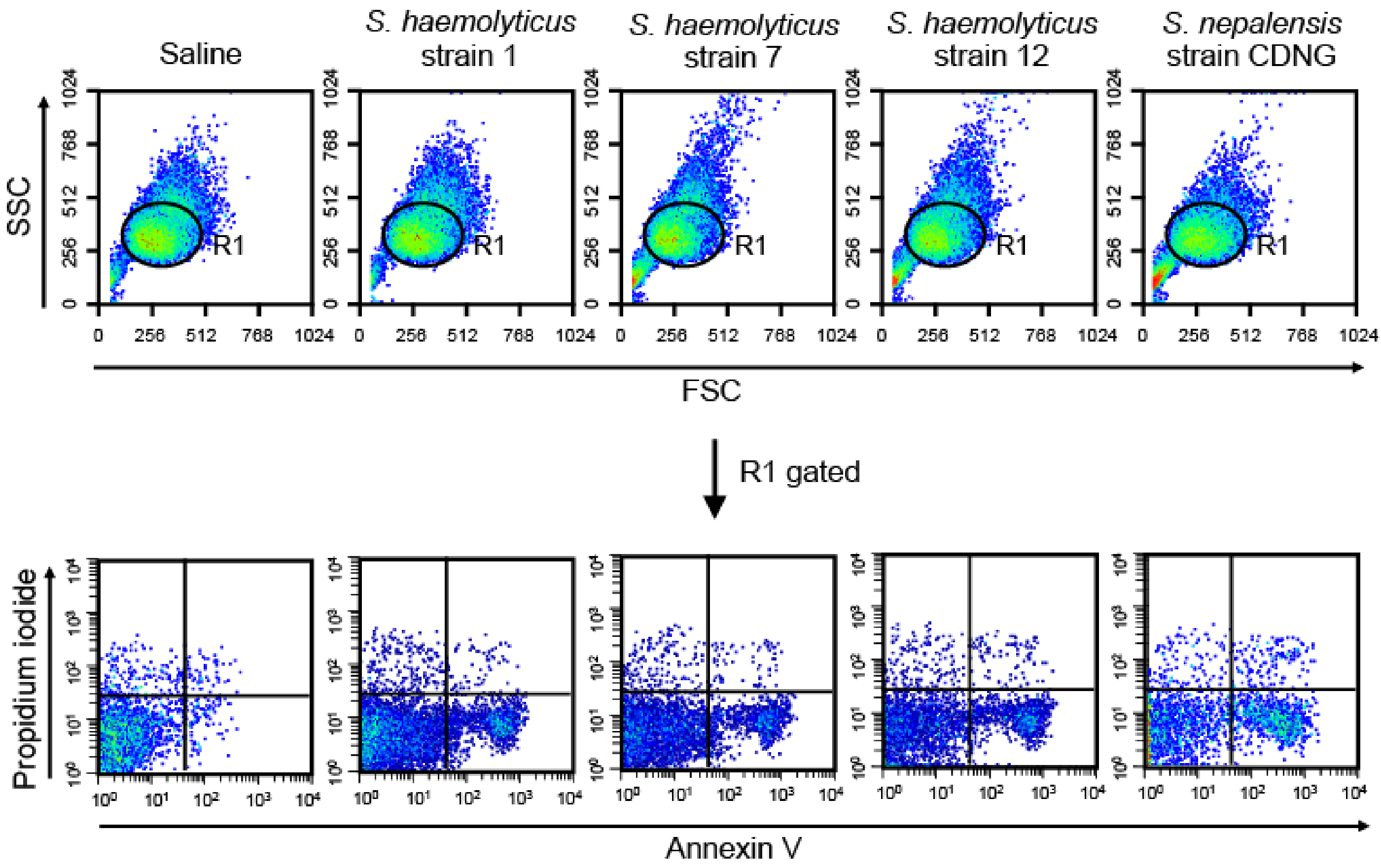

Supplementary Fig. 36. Flow cytometry gating strategy. Gating strategies used in the experiments described in Figure 1a. Abbreviations: *S.*, *Staphylococcus*; SSC, side scatter; FSC, forward scatter.

Supplementary Table 2. Primers for RT-PCR.

Gene		Sequence (5' -> 3')	Length	Tm	Reference	Location	Size
BIRC1	Sense	TACGAAGAACTACGGCTGGAC	21	61.2	NM_004536	829-849	180 bp
	Antisense	GGTGTGATCGTCTAATGGGTCA	22	61.5		1008-987	
BIRC2	Sense	GTTCAGTGGTTCTTACTCCAGC	22	60.6	NM_001166	444-465	91 bp
	Antisense	ACTGTAGGGGTTAGTCCTCGAT	22	61.4		534-513	
BIRC3	Sense	TTTCCGTGGCTCTTATTCAAACT	23	60.2	NM_001165	399-421	96 bp
	Antisense	GCACAGTGGTAGGAACTTCTCAT	23	61.9		494-472	
BIRC4	Sense	TATCAGACACCATATACCCGAGG	23	60.2	NM_001167	446-468	98 bp
	Antisense	TGGGGTTAGGTGAGCATAGTC	21	60.7		543-523	
BIRC5	Sense	AGGACCACCGCATCTCTACAT	21	62.5	NM_001012270	44-64	118 bp
	Antisense	AAGTCTGGCTCGTTCTCAGTG	21	61.7		161-141	
BIRC6	Sense	TGCACAGTTTCCTTGTACGGA	21	61.6	NM_016252	1128-1148	207 bp
	Antisense	GAGCTTGGGTCTCCTGATAGAA	22	60.6		1334-1313	
BIRC7	Sense	GCTCTGAGGAGTTGCGTCTG	20	62.5	NM_139317	254-273	245 bp
	Antisense	CACACTGTGGACAAAGTCTCTT	22	60.1		498-477	
BIRC8	Sense	GCGCTCAGAAAGACACTACAG	21	60.7	NM_033341	464-484	93 bp
	Antisense	CCTCTTGCAGACGCCTTAGC	20	62.9		556-537	
GAPDH	Sense	GGAGCGAGATCCCTCCAAAAT	21	61.6	NM_001256799	108-128	197 bp
	Antisense	GGCTGTTGTCATACTTCTCATGG	23	60.9		304-282	
BCL2	sense	GCCTTCTTTGAGTTCGGTGG	20	60.9	NM_000657	445-464	53 bp
	antisense	ATCTCCCGGTTGACGCTCT	19	62.7		497-479	
BAX	sense	CCCGAGAGGTCTTTTTCCGAG	21	62.1	NM_138763	116-136	155 bp
	antisense	CCAGCCCATGATGGTTCTGAT	21	61.9		270-250	
BCL-XL	sense	GACTGAATCGGAGATGGAGACC	22	61.6	NM_001191	120-141	179 bp
	antisense	GCAGTTCAAACTCGTCGCCT	20	63.0		298-279	
APAF1	sense	GTCACCATACATGGAATGGCA	21	60.1	NM_181868	448-468	177 bp
	antisense	CTGATCCAACCGTGTGCAAA	20	60.9		624-605	
CCND1	sense	CAATGACCCCGCACGATTTC	20	61.7	NM_053056	461-480	146 bp
	antisense	CATGGAGGGCGGATTGGAA	19	61.8		606-588	
PCNA	Forward	ACACTAAGGGCCGAAGATAACG	22	61.8	NM_002592	265-286	209 bp
	Reverse	ACAGCATCTCCAATATGGCTGA	22	61.2		473-452	
CASP3	sense	CATGGAAGCGAATCAATGGACT	22	60.7	NM_004346	64-85	139 bp
	antisense	CTGTACCAGACCGAGATGTCA	21	60.6		202-182	
CXCL1	Sense	AGGGAATTCACCCCAAGAAC	20	63.9	NM_001511	227-246	130 bp
	Antisense	ACTATGGGGGATGCAGGATT	20	64.3		356-347	
CXCL2	Sense	CTGCCAGTGCTTGCAGACC	19	67.3	NM_002089	201-219	137 bp
	Antisense	GGTTGAGACAAGCTTTCTGCC	21	65.0		337-317	
CCL2	Sense	CTCTGCCGCCCTTCTGTGCCTG	22	74.0	NM_002982	82-103	539 bp
(MCP-1)	Antisense	ACATCCCAGGGGTAGAACTCTGG	23	72.0		620-602	
CCL3	Sense	GGCTCTCTGCAACCAGTTCT	20	63.5	NM_002983	130-149	174 bp

	Antisense	CAGACCTGCCGGCTTCGCT	19	72.2		303-285	
CXCL8	Sense	GCAGCTCTGTGTGAAGGTGCA	21	69.1	NM_000584	202-222	186 bp
(IL-8)	Antisense	CAGACAGAGCTCTCTTCCAT	20	58.7		387-368	

BIRC, baculoviral inhibitors of apoptosis repeat containing (BIRC) protein; GAPDH, glyceraldehyde 3-phosphate dehydrogenase; Bcl-2, B-cell lymphoma 2; Bax, Bcl-2-associated X protein; BclXL, B-cell lymphoma-extra large. APAF-1, apoptotic protease activating factor 1; CCND1, cyclin D1; PCNA, proliferating cell nuclear antigen. CXCL, C-X-C motif chemokine ligand; CCL2, chemokine (C-C motif) ligand 2; MCP-1, monocyte chemoattractant protein-1; IL-8, interleukin-8.

Western blot images

Figure 1c

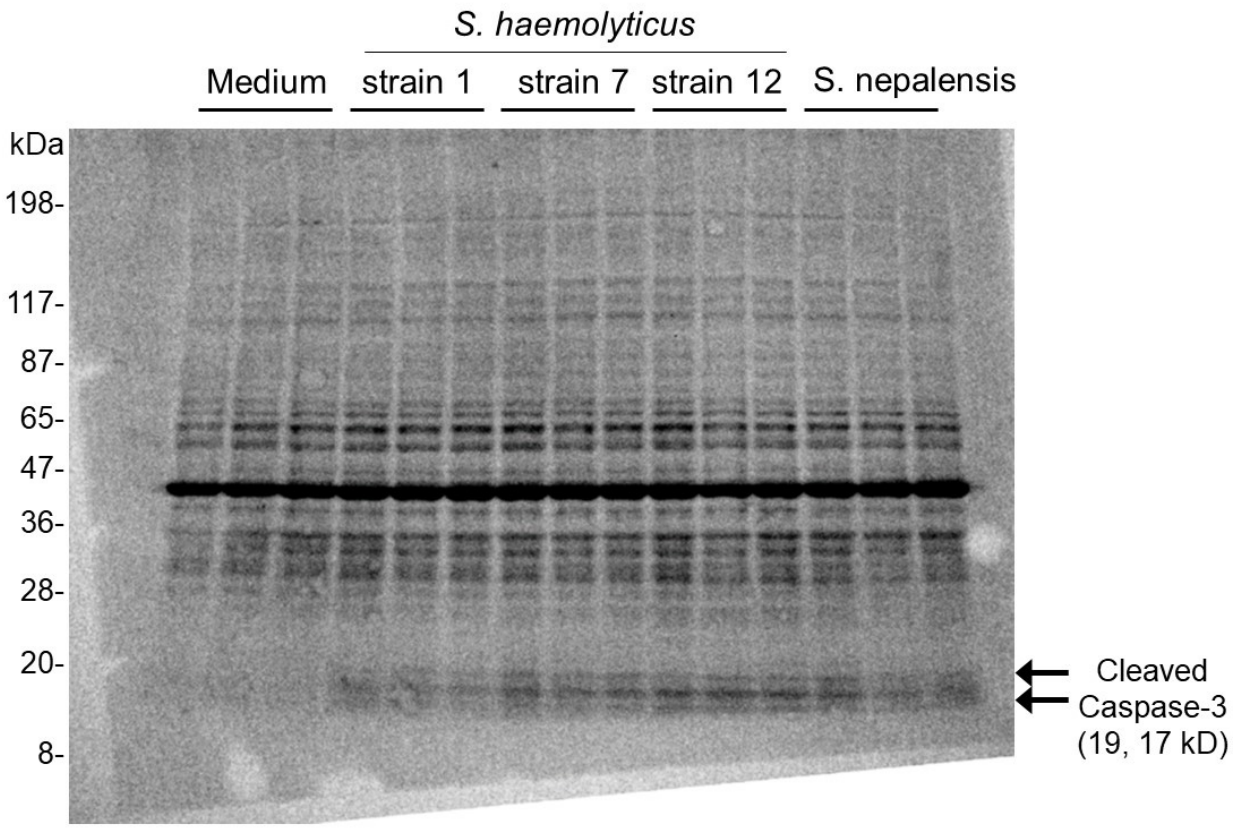

Figure 1c

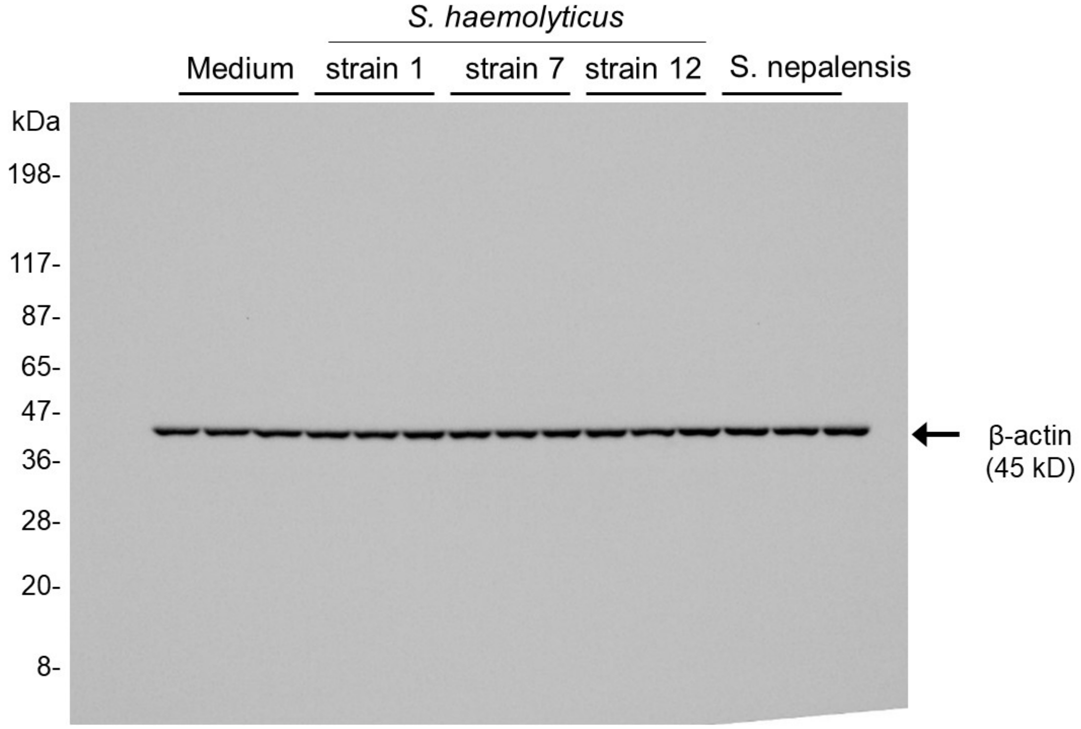

Figure 6d

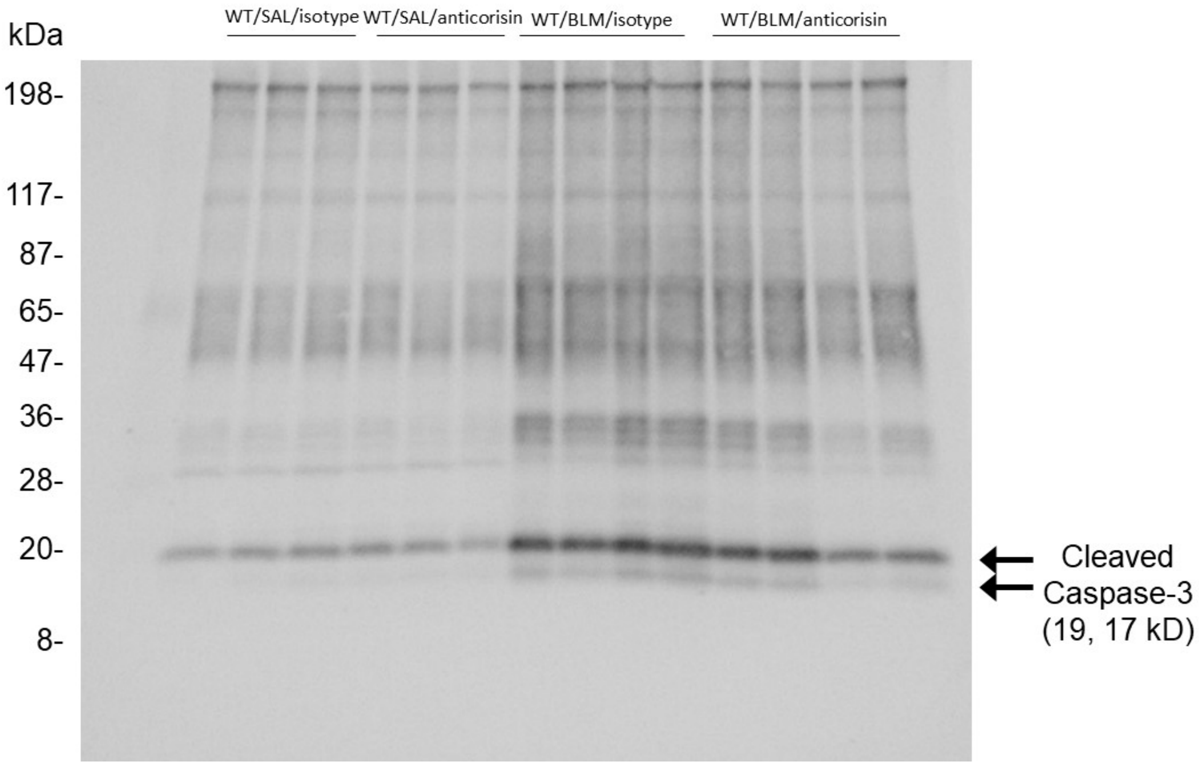

Figure 6d

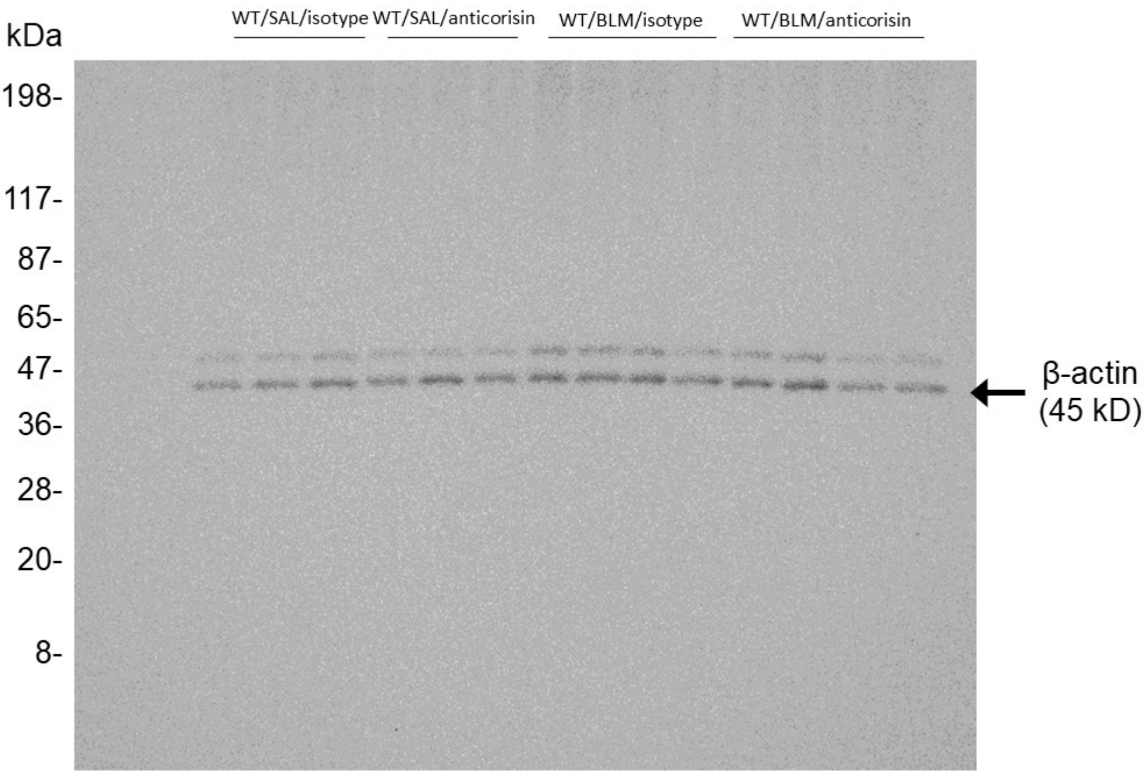

Supplementary Fig 5c

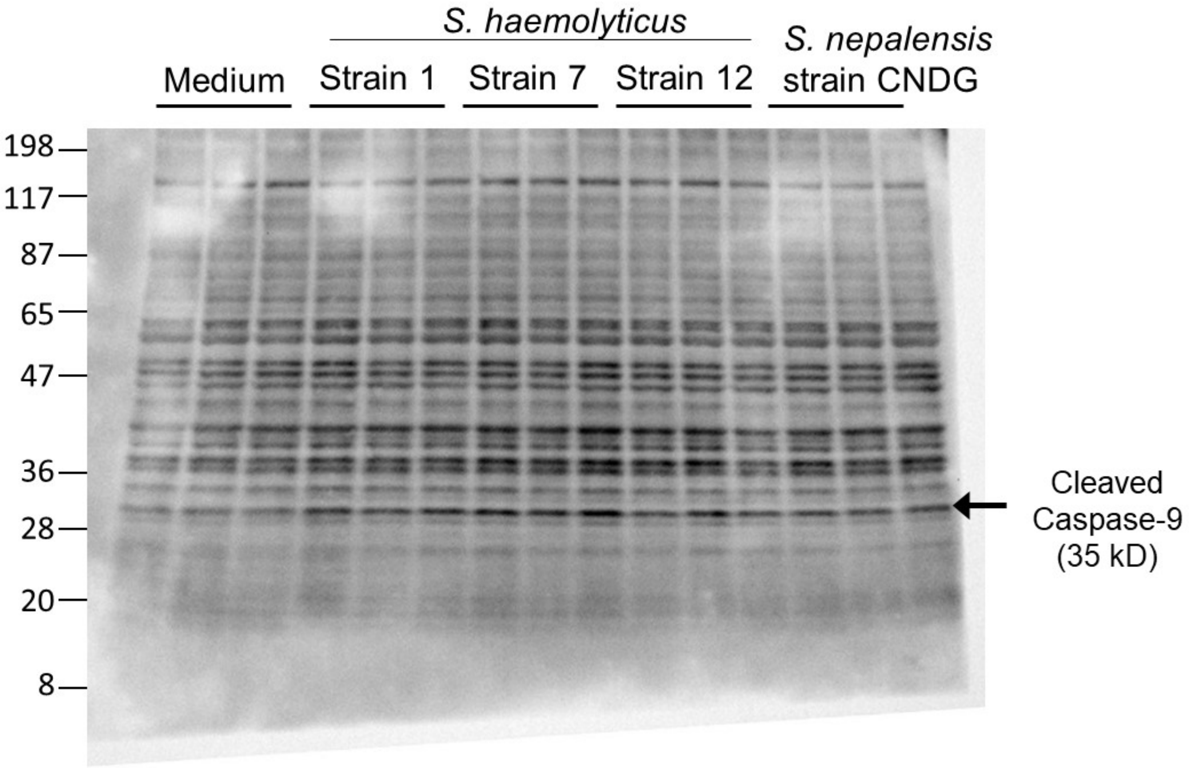

Supplementary Fig 5c

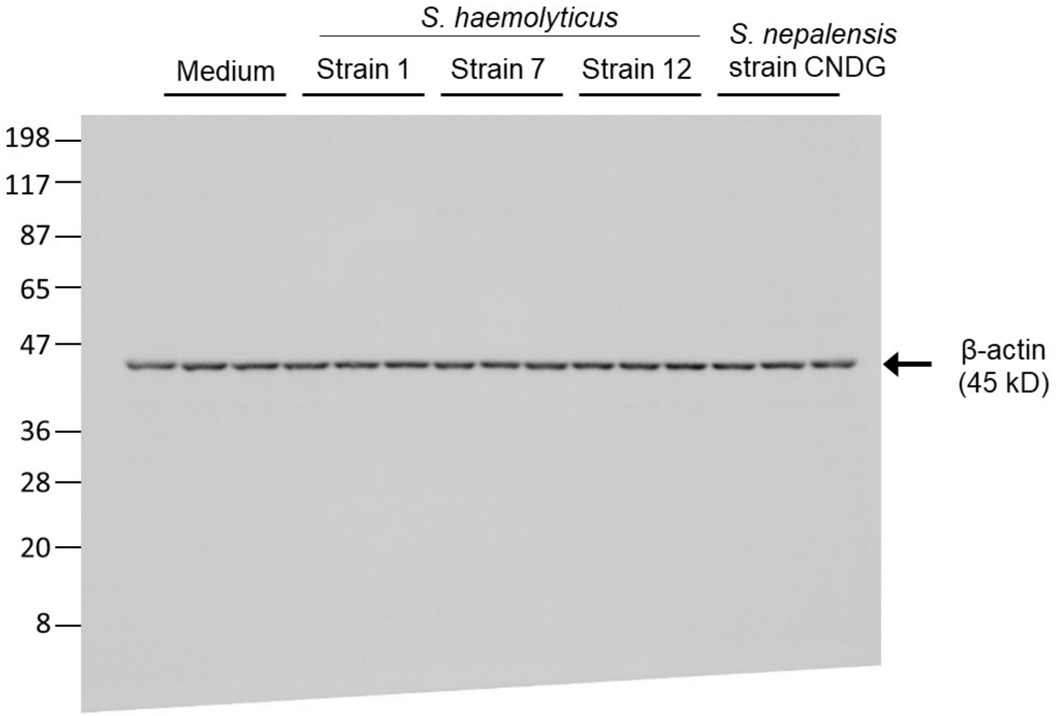

Supplementary Fig 12b

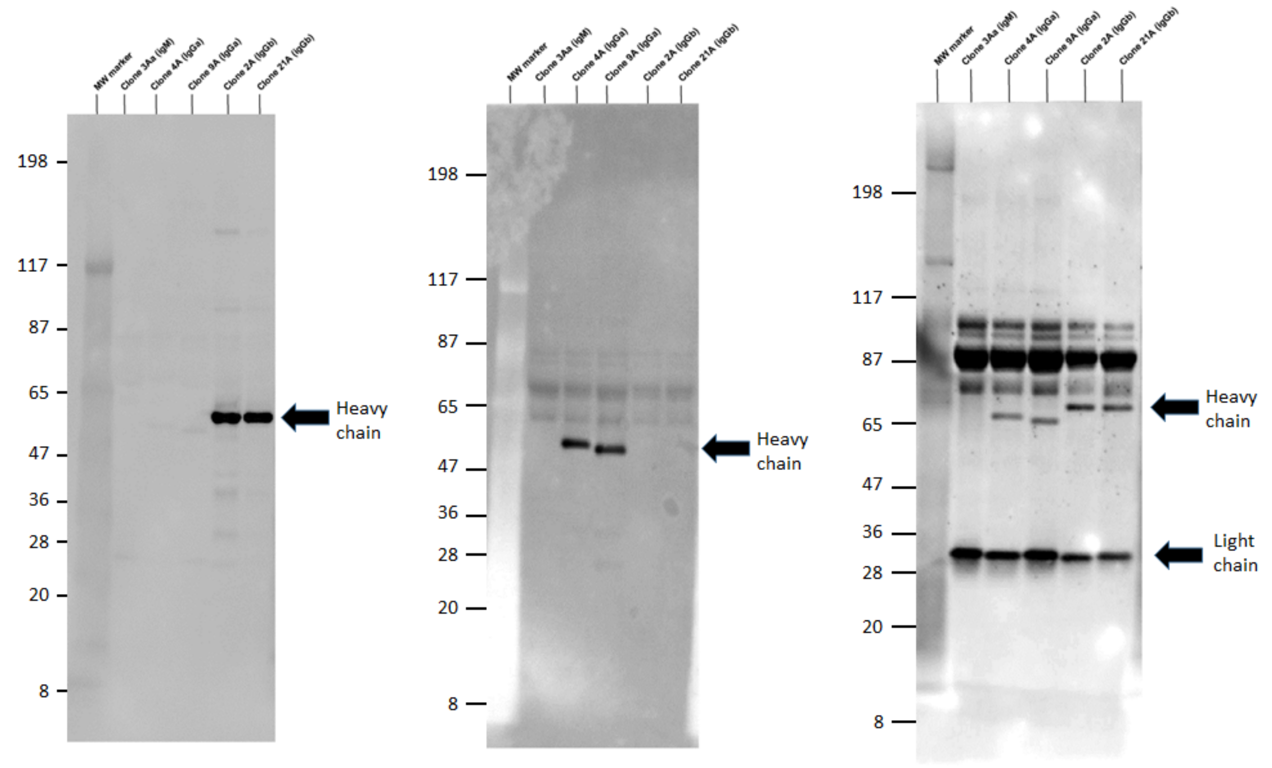

Supplementary Figure 22d

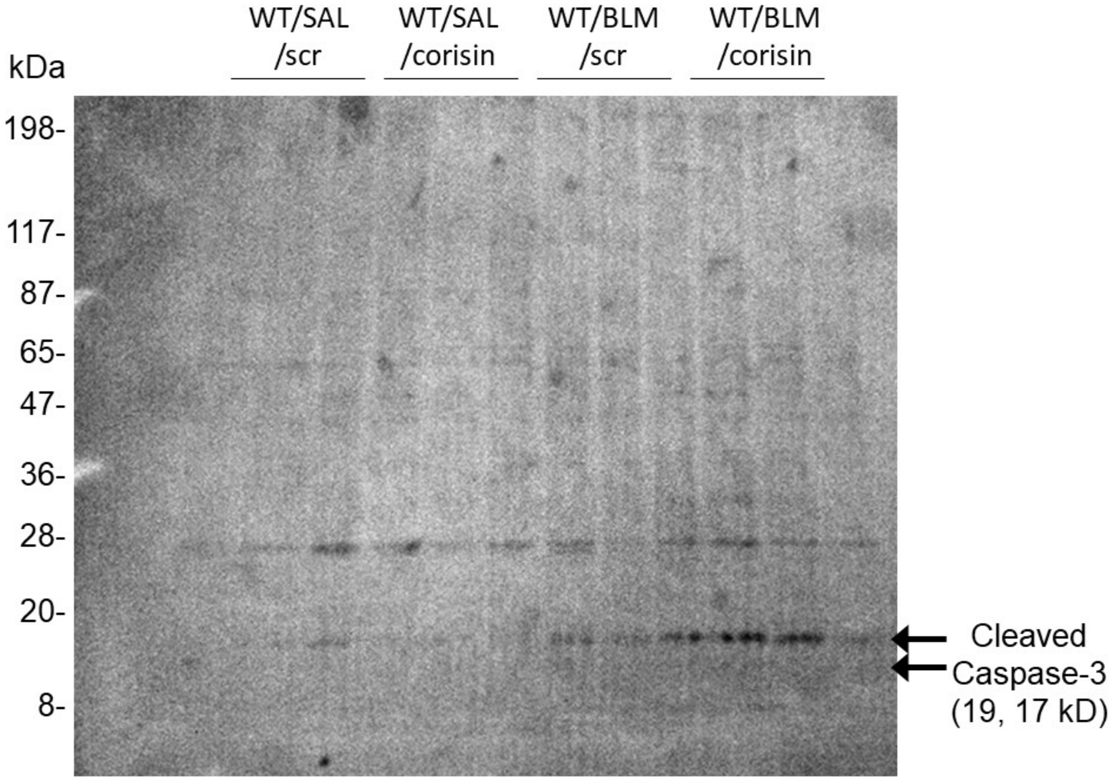

Supplementary Figure 22d

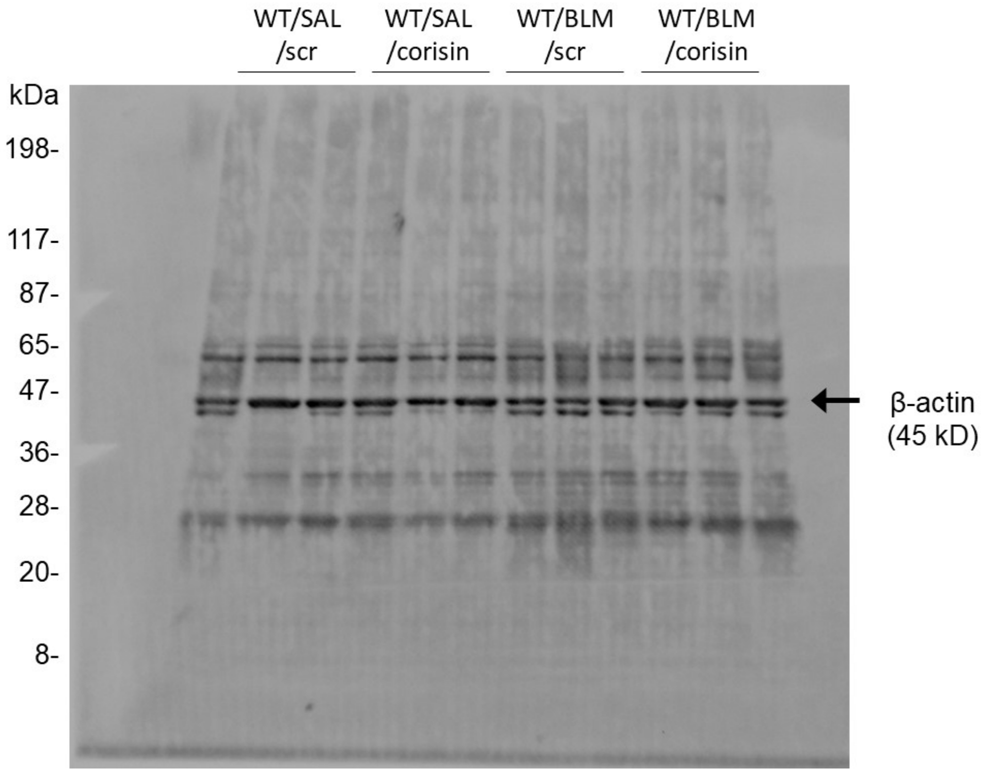

Supplementary Fig 27e

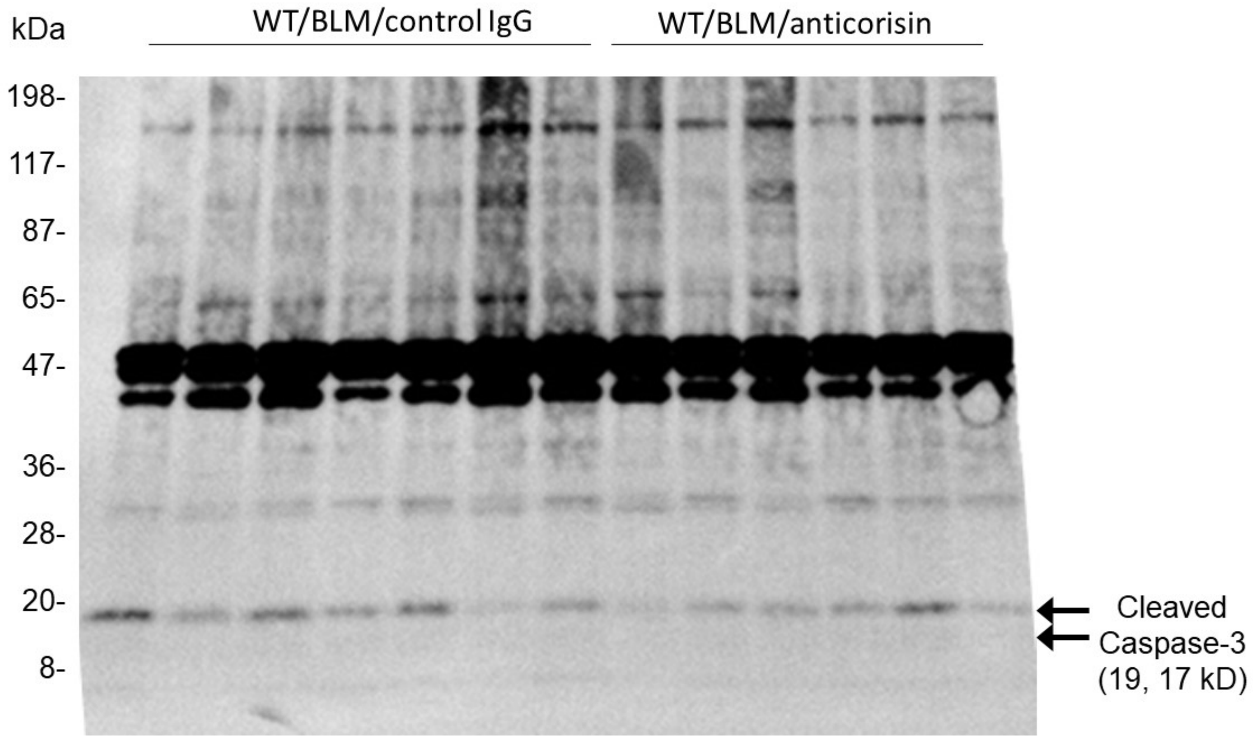

Supplementary Fig 27e

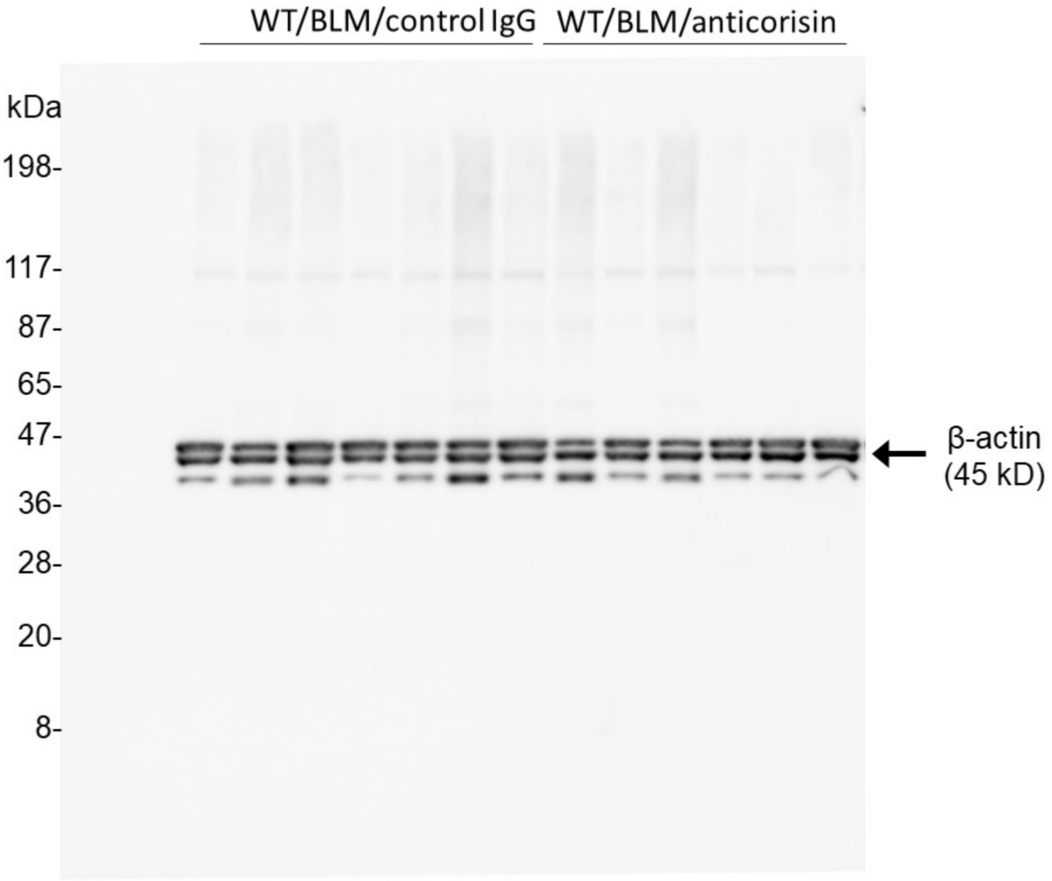

Supplementary Fig 32b

β-actin

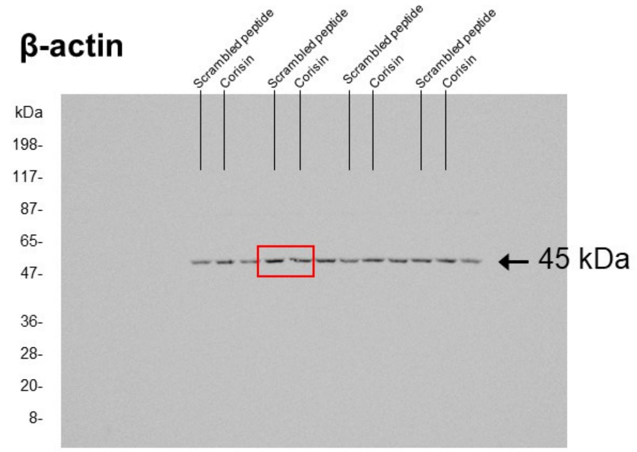

phospho-IκB-α

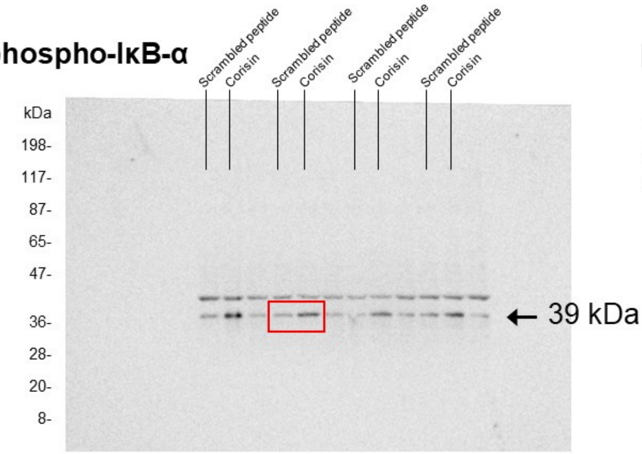

IκB-α

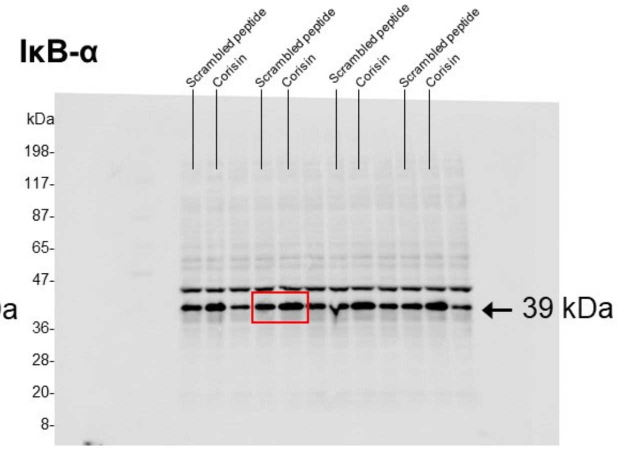

Supplementary Fig 32b

phospho-p65

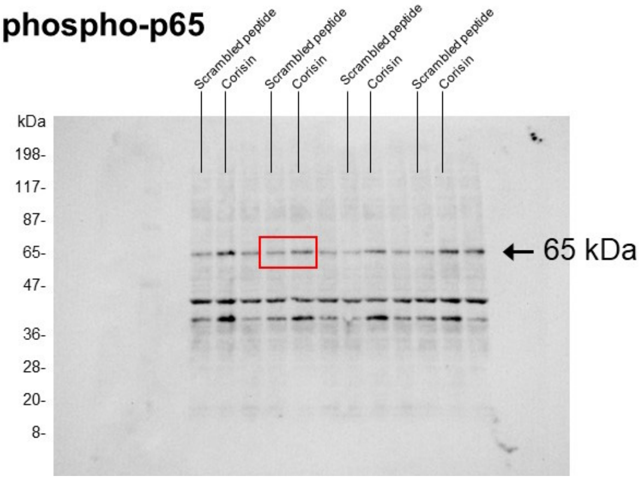

p65

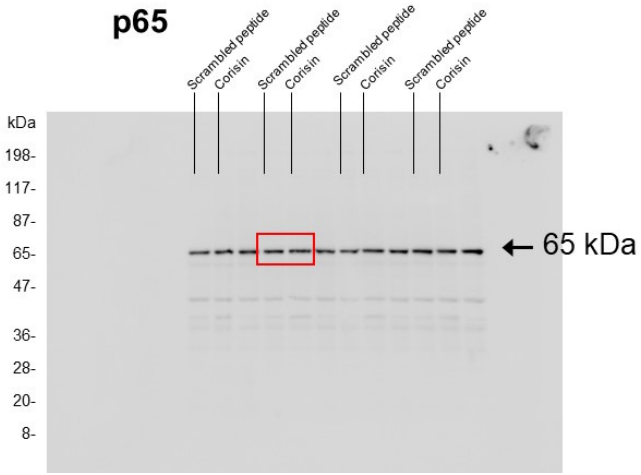

Supplement: Supplementary file 1 — Supplementary Information [file 41467_2022_29064_MOESM1_ESM.pdf]
