# Supplementary material for: Inhibition of lung microbiota-derived proapoptotic peptides ameliorates acute exacerbation of pulmonary fibrosis
Source: Nat Commun. 2022 Mar 23;13:1558. doi: 10.1038/s41467-022-29064-3 (PMC8943153; doi:10.1038/s41467-022-29064-3)
Supplement: Supplementary file 3 — Reporting Summary [file 41467_2022_29064_MOESM3_ESM.pdf]

nature research

Corresponding author(s): Esteban C. Gabazza and Isaac Cann

Last updated by author(s): Jan 14, 2022

Reporting Summary

Nature Research wishes to improve the reproducibility of the work that we publish. This form provides structure for consistency and transparency in reporting. For further information on Nature Research policies, see our Editorial Policies and the Editorial Policy Checklist.

Statistics

For all statistical analyses, confirm that the following items are present in the figure legend, table legend, main text, or Methods section.

n/a	Confirmed	
☐	☒	The exact sample size (*n*) for each experimental group/condition, given as a discrete number and unit of measurement
☐	☒	A statement on whether measurements were taken from distinct samples or whether the same sample was measured repeatedly
☐	☒	The statistical test(s) used AND whether they are one- or two-sided<br>*Only common tests should be described solely by name; describe more complex techniques in the Methods section.*
☐	☒	A description of all covariates tested
☐	☒	A description of any assumptions or corrections, such as tests of normality and adjustment for multiple comparisons
☐	☒	A full description of the statistical parameters including central tendency (e.g. means) or other basic estimates (e.g. regression coefficient) AND variation (e.g. standard deviation) or associated estimates of uncertainty (e.g. confidence intervals)
☐	☒	For null hypothesis testing, the test statistic (e.g. *F*, *t*, *r*) with confidence intervals, effect sizes, degrees of freedom and *P* value noted<br>*Give P values as exact values whenever suitable.*
☒	☐	For Bayesian analysis, information on the choice of priors and Markov chain Monte Carlo settings
☒	☐	For hierarchical and complex designs, identification of the appropriate level for tests and full reporting of outcomes
☐	☒	Estimates of effect sizes (e.g. Cohen's *d*, Pearson's *r*), indicating how they were calculated

Our web collection on statistics for biologists contains articles on many of the points above.

Software and code

Policy information about availability of computer code

Data collection	The WinRoof software version 6.5 (Mitani Corporation, Fukui, Japan)<br>Olympus BX50 microscope with an OlympusDP70 digital camera was used for data collection<br>The Prokka software tool was used for genome annotations, and the Pilon tool for correction and calling sequence variants.<br>The the public domain NIH ImageJ vs. 1.53e program was used.
Data analysis	We used GraphPad Prism vs 7 (GraphPad Software, Inc., San Diego, CA) to perform the statistical analysis.<br>The public domain NIH ImageJ program was used for image analysis.

For manuscripts utilizing custom algorithms or software that are central to the research but not yet described in published literature, software must be made available to editors and reviewers. We strongly encourage code deposition in a community repository (e.g. GitHub). See the Nature Research guidelines for submitting code & software for further information.

Data

Policy information about availability of data

All manuscripts must include a data availability statement. This statement should provide the following information, where applicable:

- Accession codes, unique identifiers, or web links for publicly available datasets
- A list of figures that have associated raw data
- A description of any restrictions on data availability

The authors declare that all data supporting this study's findings are available within this manuscript and its supplementary files. The complete genome sequences of the bacteria designated S. haemolyticus strain 1 (accession No: CP071512-CP071515), S. haemolyticus strain 7 (accession No: CP071508-CP071511) and S. haemolyticus strain 12 (accession number: CP071505-CP071507) have been deposited at the Genbank database (https://www.ncbi.nlm.nih.gov/genbank/). All raw data and sequences are also available at the open-access repository Zenodo (https://zenodo.org/record/5803063#.YcWY-WDP2ck). All datasets generated during

and/or analyzed during the current study are also available from the corresponding authors on reasonable request. Source data are provided with this paper.

Field-specific reporting

Please select the one below that is the best fit for your research. If you are not sure, read the appropriate sections before making your selection.

☒ Life sciences ☐ Behavioural & social sciences ☐ Ecological, evolutionary & environmental sciences

For a reference copy of the document with all sections, see nature.com/documents/nr-reporting-summary-flat.pdf

Life sciences study design

All studies must disclose on these points even when the disclosure is negative.

Sample size	The sample size was determined using the freely downloadable software G Power. (Faul, F., Erdfelder E, Lang A-G & Buchner A. G*Power: A flexible statistical power analysis program for the social, behavioral, and biomedical sciences. Behavioral Research Methods 39: 1775-191, (2007)
Data exclusions	No exclusion of data was done.
Replication	All in vitro experiments were performed in replicates. Most of the in vivo experiments were repeated at least two times. All measurements were reproducible and all attempts of replication were successful.
Randomization	The experimental animals were randomized for their allocation in groups. In the experiments for bleomycin-induced lung fibrosis the mice were age- and sex-matched (only female mice) and they were randomly allocated in different groups and cages before starting the experiments. In experiments using the transforming growth factorbeta1 transgenic (TGFbeta1 TG) mice with lung fibrosis, the mice were also age-matched and sex-matched (only males or only females in all groups). Lung fibrosis was scored in all TGFBeta1 TG mice by chest computed tomography (CT) and the mice with equal CT scores (grade of lung fibrosis) were allocated randomly into each treatment group before starting the experiments. For evaluation of acute exacerbation of lung fibrosis using the bleomycin model, chest CT was performed in all mice and mice with equal CT score of lung fibrosis were randomly allocated into each treatment group before the induction of acute exacerbation or starting the experiments. In some experiments randomization was not done because it was not relevant (e.g., measurement of longitudinal changes of corisin in mice after treatment with bleomycin, or mice used for measuring the half-life of the monoclonal antibodies after intraperitoneal injection).
Blinding	Some sets of data such as radiological findings, scoring of lung fibrosis and area of collagen deposition were analyzed in a double-blind approach. The researchers that did the chest radiological study, the researchers that scored the grade of lung fibrosis, and the researchers that randomly allocated the age- and sex-matched mice (with similar CT score) into each treatment were all different. The authors that measured parameters in the samples were unaware of the treatment groups. The researchers that performed PCR or immune assays in samples taken from mice are not involved in randomization, allocation or treatment of mice.

Reporting for specific materials, systems and methods

We require information from authors about some types of materials, experimental systems and methods used in many studies. Here, indicate whether each material, system or method listed is relevant to your study. If you are not sure if a list item applies to your research, read the appropriate section before selecting a response.

Materials & experimental systems

n/a	Involved in the study	
☐	☒	Antibodies
☐	☒	Eukaryotic cell lines
☒	☐	Palaeontology and archaeology
☐	☒	Animals and other organisms
☐	☒	Human research participants
☒	☐	Clinical data
☒	☐	Dual use research of concern

Methods

n/a	Involved in the study	
☐	☒	ChIP-seq
☐	☒	Flow cytometry
☒	☐	MRI-based neuroimaging

Antibodies

Antibodies used	The following antibodies were used: anti-cleaved caspase-3 (dilution 1:1000; cat. 9664S; clone D175 (5AIE); lot. 13), anti-cleaved caspase-9 (dilution 1:1000; cat. 20750S; clone Asp315 (D8I9E); lot. 1), anti-total IB (dilution 1:000; cat. 4812S; clone D44D4; lot. 6) or anti-phosphorylated IB (dilution 1:000; cat. 2859S; clone 14D4; lot. 8), anti-total p65 (dilution 1:000; cat. 8242S; clone D14E12XP(R); lot. 0004) or anti-phosphorylated p65 (dilution 1:1000; cat. 3033S; clone S536 (93H1); lot. 0014), or anti-βactin antibody (dilution 1:2000; cat. 4970T; clone 13E5; lot. 18) from Cell Signaling (Danvers, MA), or mouse monoclonal anti-rat IgG2b (dilution 1:1000; cat. 106750; clone KT98 (HRP); lot. GR3324821-11) (abcam, Cambridge, UK), anti-rat IgG2a (dilution 1:1000; cat. A110; clone 109P; lot. 34) (Bethyl Laboratories, Montgomery, TX) or anti-rat IgG, IgA, IgM (dilution 1:1000; cat.800-656-7625; clone p/m612-103-130; lot. 36609) (Rockland Immunochemicals, Gilbertsville, PA), antibody

Validation	Antibodies were validated by the manufacturers: Cell Signaling Technology: https://www.cellsignal.com/about-us/our-approach-process/cst-antibody-validation-principles); abcam: https://www.abcam.com/resources?keywords=validation%20antibodies; Bethy laboratories: https://everonlife.com/our-products/bethyl-laboratories-inc/; Rockland Immunochemicals: https://www.rockland.com/.

Eukaryotic cell lines

Policy information about cell lines

Cell line source(s)	The A549 cell lines from the American Type Culture Collection (Manassas, VA) and the normal human bronchial epithelial (NHBE) cells from Lonza (Walkersville, MD). were used in the experiments.
Authentication	The A549 cell lines and the normal human bronchial epithelial (NHBE) cells were authenticated by their morphology and the expression of specific genes (surfactant protein-C: positive expression in A549 cells and negative expression in NHBE cells) by PCR using specific primers.
Mycoplasma contamination	There was no Mycoplasma contamination
Commonly misidentified lines (See ICLAC register)	Commonly misidentified cell lines were not used in the study.

Animals and other organisms

Policy information about studies involving animals; ARRIVE guidelines recommended for reporting animal research

Laboratory animals	Mice (males and females) aging between 8 and 12 weeks with a C57BL/6 background were used in the experiments. The mice were bred in a specific pathogen-free environment at a temperature of 21°C, relative humidity of 50-70% and under a constant 12-h light/dark cycle in the facility for experimental animals of Mie University. The plastic cage of the mice was supplied with wood-wool nesting material, and mice had access to water and food (standard bait, CE-7 pellet, CLEA Japan Incorporation) ad libitum. Female Wistar rats (6-weeks of age) were bred and used at Eurofins Genomics Incorporation (Tokyo, Japan) for antibody production.
Wild animals	Wild animals were not used in the study.
Field-collected samples	Field-collected samples were not used in the study.
Ethics oversight	The Recombinant DNA Experiment Safety Committee (approval No: I-614 (henko1); date: 2013/15/12; approval No: I-708, date: 13/02/2019) and the Committee for Animal Investigation of Mie University approved the experimental protocols (approval No: 25-20-hen1-sai1, date: 23/07/2015; approval No: 29-23, date: 15/-01/2019). We performed all experimental procedures following internationally approved laboratory animal care principles published by the National Institute of Health (https://olaw.nih.gov/)

Note that full information on the approval of the study protocol must also be provided in the manuscript.

Human research participants

Policy information about studies involving human research participants

Population characteristics	This study comprised 36 Japanese patients (males, 34; females, 2; age average 66 years-old) with IPF and 6 Japanese male healthy volunteers
Recruitment	All consecutive IPF patients consulted or were referred to the health care institutions (Tosei General Hospital, Aichi, Japan or the National Hospital Organization Kinki-Chuo Chest Medical Center, Osaka, Japan) for breathlessness. These two health care institutions where the patients were treated are specialized hospitals for treating IPF patients.
Ethics oversight	All subjects participating in the clinical investigation provided written informed consent and the study protocol was approved by the Ethical Committees for Clinical Investigation of Mie University (approval No: H2019064, date: 25/04/2019), Matsusaka Municipal Hospital (approval date: 11/06/2014) and conducted following the Principles of the Declaration of Helsinki

Note that full information on the approval of the study protocol must also be provided in the manuscript.

ChIP-seq

Data deposition

- [x] Confirm that both raw and final processed data have been deposited in a public database such as GEO.
- [x] Confirm that you have deposited or provided access to graph files (e.g. BED files) for the called peaks.

Data access links *May remain private before publication.*	The complete genome sequences of the bacteria designated S. haemolyticus strain 1 (accession No: CP071512-CP071515), S. haemolyticus strain 7 (accession No: CP071508-CP071511) and S. haemolyticus strain 12 (accession number: CP071505-CP071507) have been deposited at the Genbank database (https://www.ncbi.nlm.nih.gov/genbank/).
Files in database submission	The whole DNA sequence of cultured bacteria

Genome browser session (e.g. UCSC)

not applicable

Methodology

Replicates	All replicates were reproducible
Sequencing depth	The majority of reads were 6 kb to 30 kb, although reads as long as 94 were also obtained.
Antibodies	*Describe the antibodies used for the ChIP-seq experiments; as applicable, provide supplier name, catalog number, clone name, and lot number.*
Peak calling parameters	Genomic DNA from the bacterial strain was converted into a nanopore library with the Rapid Barcoding Library kit SQK-RAD004. The library was sequenced on a SpotON R9.4.1FLO-MIN106 flowcell for 48h on a GridON sequencer. Basecalling was performed with Guppy 1.4.3, and demultiplexing was done with Porechops 0.2.3
Data quality	A workflow was developed to perform four assemblies as follows: primarily to assess quality using different assembly strategies to find the best overall assembly. Initial assembly of the Oxford Nanopore data was carried out using Camu, followed by polishing using Nanopolish amd Pilon (utilizaing the Illumina MiSeq reads), and finally the genome was re-oriented using Circlator
Software	The Illumina Miseq sequencing was carried out by preparing shotgun genomic libraries with the Hyper Library construction kit from Kapa Biosystems (Roche). The library was quantitated by qPCR and sequenced on one MiSeq Nano flowcell for 251 demultiplexed with the bcl2fastq v2.20 Conversion Software (Illumina)

Flow Cytometry

Plots

Confirm that:

- [x] The axis labels state the marker and fluorochrome used (e.g. CD4-FITC).
- [x] The axis scales are clearly visible. Include numbers along axes only for bottom left plot of group (a 'group' is an analysis of identical markers).
- [x] All plots are contour plots with outliers or pseudocolor plots.
- [x] A numerical value for number of cells or percentage (with statistics) is provided.

Methodology

Sample preparation	A549 and normal human bronchial epithelial cel ls were cultured in DMEM supplemented with 10% fetal calf serum and antibiotics at 5% CO2 atmosphere at 37 °C. The cells were washed and th en treated with propidium iodide and annexin V to evalu ate apoptosis or with the specific antibody to evaluate specific cell population.
Instrument	We used a flow cytometer (FACScan) from BD Biosciences
Software	Cellquest software from 8D Biosciences was used for data analysis
Cell population abundance	Sufficient number of cells was used in each experiment (500,000 to 1,500,000 cells per experiment).
Gating strategy	Gates were placed around cell populations with common characteristics after determining forward scatter, side scatter and marker expression.

- [x] Tick this box to confirm that a figure exemplifying the gating strategy is provided in the Supplementary Information.
